# Targeting epigenetic and posttranslational modifications regulating ferroptosis for the treatment of diseases

**DOI:** 10.1038/s41392-023-01720-0

**Published:** 2023-12-10

**Authors:** Yumin Wang, Jing Hu, Shuang Wu, Joshua S. Fleishman, Yulin Li, Yinshi Xu, Wailong Zou, Jinhua Wang, Yukuan Feng, Jichao Chen, Hongquan Wang

**Affiliations:** 1 Department of Respiratory and Critical Care Medicine, Aerospace Center Hospital, Peking University Aerospace School of Clinical Medicine, Beijing, 100049 PR China; 2 Department of Pathogen Biology, School of Basic Medical Sciences, Tianjin Medical University, Tianjin, 300060 PR China; 3 Department of Neurology, Zhongnan Hospital of Wuhan University, Wuhan, 430000 PR China; 4 Department of Pharmaceutical Sciences, College of Pharmacy and Health Sciences, St. John’s University, Queens, NY 11439 USA; 5 Department of Outpatient, Aerospace Center Hospital, Peking University Aerospace School of Clinical Medicine, Beijing, 100049 PR China; 6 Beijing Key Laboratory of Drug Target and Screening Research, Institute of Materia Medica, Chinese Academy of Medical Sciences and Peking Union Medical College, Beijing, 100050 PR China; 7 Department of Pancreatic Cancer, Tianjin Medical University Cancer Institute and Hospital, National Clinical Research Center for Cancer, Tianjin’s Clinical Research Center for Cancer, Key Laboratory of Cancer Prevention and Therapy, Tianjin, 300060 PR China

**Keywords:** Drug development, Drug development

## Abstract

Ferroptosis, a unique modality of cell death with mechanistic and morphological differences from other cell death modes, plays a pivotal role in regulating tumorigenesis and offers a new opportunity for modulating anticancer drug resistance. Aberrant epigenetic modifications and posttranslational modifications (PTMs) promote anticancer drug resistance, cancer progression, and metastasis. Accumulating studies indicate that epigenetic modifications can transcriptionally and translationally determine cancer cell vulnerability to ferroptosis and that ferroptosis functions as a driver in nervous system diseases (NSDs), cardiovascular diseases (CVDs), liver diseases, lung diseases, and kidney diseases. In this review, we first summarize the core molecular mechanisms of ferroptosis. Then, the roles of epigenetic processes, including histone PTMs, DNA methylation, and noncoding RNA regulation and PTMs, such as phosphorylation, ubiquitination, SUMOylation, acetylation, methylation, and ADP-ribosylation, are concisely discussed. The roles of epigenetic modifications and PTMs in ferroptosis regulation in the genesis of diseases, including cancers, NSD, CVDs, liver diseases, lung diseases, and kidney diseases, as well as the application of epigenetic and PTM modulators in the therapy of these diseases, are then discussed in detail. Elucidating the mechanisms of ferroptosis regulation mediated by epigenetic modifications and PTMs in cancer and other diseases will facilitate the development of promising combination therapeutic regimens containing epigenetic or PTM-targeting agents and ferroptosis inducers that can be used to overcome chemotherapeutic resistance in cancer and could be used to prevent other diseases. In addition, these mechanisms highlight potential therapeutic approaches to overcome chemoresistance in cancer or halt the genesis of other diseases.

## Introduction

Ferroptosis, a new form of regulated cell death (RCD), is driven by iron-dependent lipid peroxidation (LPO) of polyunsaturated fatty acid-containing phospholipids (PUFA-PLs) in cellular membranes.^1–4^ Ferroptosis was first reported in 2012 and was found to be induced by erastin, an oncogenic RAS-selective lethal chemical.^4^ Ferroptosis was officially identified as a non-apoptotic RCD triggered by intracellular iron perturbations and oxidative stress.^5^ A imbalance between ferroptosis defense and systems dictates the execution and induction of ferroptosis.^6^ Many metabolic pathways and degradation pathways orchestrate the complex response to ferroptosis by indirectly or directly regulating LPO or iron accumulation.^7^ The metabolic pathways include pathways related to lipid, iron, and amino acid metabolism, and the degradation pathways include pathways such as the ubiquitin–proteasome system (UPS) and macroautophagy/autophagy.

Accumulating evidence suggests that ferroptosis is precisely regulated at multiple levels that include protein posttranslational modifications (PTMs) and epigenetic modifications.^8^ Epigenetic modification that includes DNA methylation, histone modification, and noncoding RNA (ncRNA) regulation is a dynamic and reversible process, which regulates gene expression without changing the DNA sequence.^9–11^ PTMs covalently or enzymaticly modify or introduce functional groups to dynamically modulate protein localization, activity, and molecular interactions.^12,13^ PTMs include phosphorylation, ubiquitination, SUMOylation, acetylation, among others.^13^ Aberrant epigenetic modifications and PTMs dynamically drive abnormal transcription or translation processes to promote anticancer drug resistance, cancer progression, metastasis, etc. The epigenetic modifications regulate the expression levels of ferroptosis-related genes, consequently determining the vulnerability of cancer cells to ferroptosis at both the transcriptional and translational levels.^8,14–16^ Moreover, emerging evidence has revealed the roles of epigenetic modifications and PTMs in the regulation of ferroptosis in NSDs, CVDs, liver diseases, lung diseases, and kidney diseases.^17^

Dysregulated ferroptosis is increasingly recognized as a significant contributor to the pathogenesis of diseases, including cancers,^18^ nervous system diseases (NSDs),^19–23^ cardiovascular diseases (CVDs),^24–28^ liver diseases,^29,30^ lung diseases,^31–34^, and kidney diseases.^35^ Recently, ferroptosis has been recognized to play an important role in halting tumor growth.^36^ In the last decade, accumulating evidence has revealed the role of ferroptosis in tumor growth suppression and shown that ferroptosis induction partially mediates the tumor-suppressive effects of chemotherapy.^3,37^ Ferroptosis determines the efficacy of chemotherapy, immunotherapy, and radiotherapy, and thus, combination treatments with ferroptosis inducers could boost the efficacy of those therapies.^38,39^ Accumulating studies have shown that pharmacologically modulating ferroptosis may be a therapeutic approach for NSDs, CVDs, liver diseases, lung diseases, and kidney diseases.^19,25,40–45^

In this review, we first summarize the core molecular mechanisms of ferroptosis. Then, the role of epigenetic processes, including histone PTMs, DNA methylation, and ncRNA regulation, are concisely discussed. This discussion is followed by a detailed description of the roles of epigenetic regulation of ferroptosis in the genesis of diseases, including NSD, CVD, liver diseases, lung diseases, and kidney diseases, as well as the application of epigenetic modulators in the treatment of these diseases. Elucidating the epigenetic regulatory mechanisms of ferroptosis in cancer and other diseases will accelerate the development of promising combination therapeutic regimens containing epigenetic agents and ferroptosis inducers that can be used to overcome chemotherapeutic resistance in cancer and could be used to prevent other diseases. In addition, these mechanisms and highlight promising therapeutic approaches that may be used to overcome chemotherapy drug resistance in cancer or halt the genesis of other diseases.

## Molecular mechanisms of ferroptosis

**Fig. 1:**
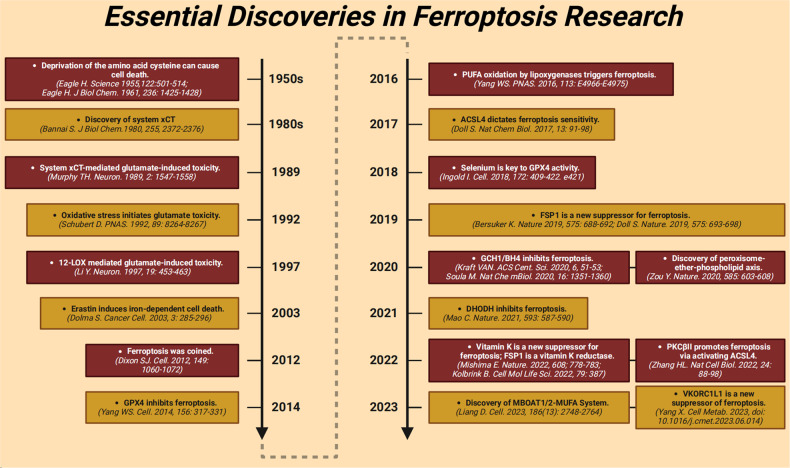
The diagram depicting key milestones in the field of ferroptosis research

**Fig. 2:**
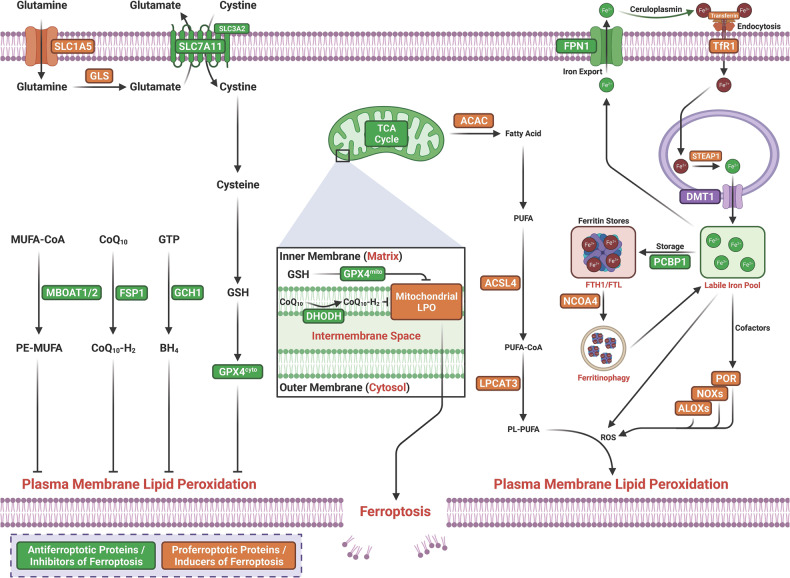
Core mechanisms of ferroptosis. The core of ferroptosis initiation is iron-dependent lipid peroxidation of polyunsaturated fatty acid (PUFA)-containing phospholipids (PUFA-PLs). When the ferroptosis-promoting factors (or Ferroptosis prerequisites) exceeding the buffering capability of cellular antioxidant systems (or ferroptosis defense systems), lethal accumulation of lipid peroxides on cellular membranes lead to membrane rupture, resulting in ferroptosis-related cell death. The ferroptosis-promoting factors consist of PUFA-PL synthesis and peroxidation, iron metabolism among others. Cells have evolved at least four ferroptosis defense systems,which includes GPX4/xCT system, the FSP1/CoQH_2_ system, the DHODH /CoQH_2_ system, and the GCH1/BH_4_ system, with different subcellular localizations to detoxify lipid peroxides and thus protect cells against ferroptosis. The cytosolic GPX4 (GPX4^cyto^) cooperates with FSP1 on the plasma membrane (and other non-mitochondrial membranes) and mitochondrial GPX4 (GPX4^mito^) cooperates with DHODH in the mitochondria to neutralize lipid peroxides. ACSL4 and LPCAT3 mediate the synthesis of PUFA-PLs, which are susceptible to LPO through both non-enzymatic and enzymatic mechanisms. Iron initiates the non-enzymatic Fenton reaction and acts as an essential cofactor for ALOXs and POR, which promote LPO. When ferroptosis-promoting factors significantly exceed the detoxification capabilities of ferroptosis defense systems, an excessive and lethal accumulation of lipid peroxides on cellular membranes result in membrane rupture and trigger ferroptosis-mediated cell death

The term ferroptosis was coined by the Stockwell laboratory in 2012 based on the three major research areas that provided a foundational understanding of ferroptosis.: the control of ROS,^46,47^ the mechanisms of amino acid and lipid metabolism,^48–50^ and the regulation of iron^2^ (Fig. 1). Iron accumulation and LPO trigger ferroptosis, resulting in plasma membrane rupture.^51^ The initiation of ferroptosis requires two key signals, namely, the accumulation of free iron and the inhibition of defense systems, mainly the solute carrier family seven members 11–glutathione–glutathione peroxidase 4 (SLC7A11–GSH–GPX4) system.^52^ The activation of ferroptosis indicates a delicate imbalance between ferroptosis-promoting factors and defense systems. When the former factors significantly override the latter antioxidant defense systems, lethal accumulation of lipid peroxides on cellular membranes leads to membrane rupture and results in ferroptosis-related cell death^3,6^ (Fig. 2). Currently, the main ferroptosis defense systems constitute the SLC7A11–GSH–GPX4 system,^6,53^ the GTP cyclohydrolase 1–tetrahydrobiopterin (GCH1–BH_4_) system,^54,55^ the ferroptosis suppressor protein 1–ubiquinol (FSP1–CoQH_2_) system,^56,57^ the dihydroorotate dehydrogenase–dihydroubiquinone (DHODH–CoQH_2_) system,^58^ and the O-acyltransferase domain containing 1/2–monounsaturated fatty acids (MBOAT1/2–MUFA) system.^59^ Many key components of the ferroptosis pathway, e.g., the principal proteins and enzymes engaged in the induction and inhibition of ferroptosis, are transcriptionally controlled by NF-E2 p45-related factor 2 (Nrf2), the transcription factor encoded by NFE2L2.^60–64^ Nrf2 and Kelch-like ECH-associated protein 1 (KEAP1), which is the principal negative regulator of Nrf2 and an E3 ligase adaptor, are critical for maintaining metabolic, redox and protein homeostasis.^65,66^ Nrf2 is involved in regulating the transcription of enzymes responsible for GSH biosynthesis, such as glutamate-cysteine ligase catalytic subunit (GCLC), glutamate-cysteine ligase modifier subunit, glutathione disulfide reductase, and GSH synthetase (GSS), which support GPX4-mediated suppression of ferroptosis by increasing and maintaining the GSH level. Other downstream targets of Nrf2 include SLC7A11, GPX4, and NAD(P)H-quinone oxidoreductase 1, as well as iron metabolism proteins, such as ferritin light chain (FTL), ferritin heavy chain 1 (FTH1), ferroportin-1 (FPN1) and heme oxygenase-1 (HO-1), all of which are directly relevant to ferroptosis.^60–64,67–69^

### Ferroptosis-promoting factors

#### PUFA-PL synthesis and peroxidation

The core mechanism of ferroptosis is membrane LPO, a radical-induced chain reaction consisting of a series of chemical reactions between molecular oxygen (O_2_), oxidizable lipids, and iron, leading to the incorporation of O_2_ into lipids.^70,71^ Because of their susceptibility to peroxidation, PUFA-PLs are the substrates for LPO during ferroptosis.^70,72^ The critical mediators of the synthesis of PUFA-PLs include acyl-coenzyme A (CoA) synthetase long-chain family member 4 (ACSL4) and lysophosphatidylcholine acyltransferase 3 (LPCAT3).^73,74^ ACSL4 ligates the free long-chain PUFAs adrenic acid (AdA) and arachidonic acid (AA) to CoA to generate AA-CoA and ADA-CoA (i.e., PUFA-CoAs), respectively.^74,75^ Subsequently, these PUFA-CoAs are re-esterified and incorporated into PLs by LPCAT3 to produce PUFA-PLs (such as AA-phosphatidylethanolamine [PE] and ADA-PE).^73,75,76^ PKCβII-mediated phosphorylation of ACSL4 can further activate ACSL4.^77^ Acetyl-CoA carboxylase mediates the synthesis of PUFAs from the basic building block acetyl-CoA.^72^ Nonenzymatic autoxidation through the iron-mediated Fenton reaction is the primary driver of LPO of PUFA-PLs.^70,78,79^ The enzymatic reactions mediated by arachidonate lipoxygenase (ALOX) or Cytochrome P450 oxidoreductase are also involved in facilitating LPO.^80–84^ final step of ferroptosis is the formation of pores in plasma or organelle membranes driven by LPO or its secondary products (4-hydroxynonenal and malondialdehyde), which eventually results in cell death. During recent decades, the involvement of ferroptosis in diseases has attracted great interest, not only in the cancer research community.^85,86^

#### Iron homeostasis

Free iron is involved in the core mechanism of ferroptosis in at least two different ways.: inducing accumulation of lethal lipid peroxides and initiating ferroptosis by catalyzing the nonenzymatic Fenton reaction for direct peroxidation of PUFA-PLs^70,79^ or functioning as an essential cofactor for POR and ALOX, both of which are enzymes that participate in LPO.^52,80,81,87^ Mammalian cells contain a relatively stable pool of intracellular iron—the labile iron pool (LIP)—and maintain this pool by orchestrating the regulation of iron uptake, utilization, storage (ferritin: FTH1/FTL) and export (FPN, the iron export transporter).^88,89^ Deregulation of iron metabolism processes can suppress or promote ferroptosis as a result of a decrease or increase in intracellular LIP, respectively. Intracellular iron is mostly stored in the ferritin protein complex, which is composed of 24 subunits of FTL and FTH1.^90^ Poly rC-binding protein 1 binds to and incorporates ferrous iron (Fe^2+^) into ferritin, and this Fe^2+^ is further oxidized to ferric iron (Fe^3+^) by FTH1 inside the ferritin cage, resulting in inert deposits of Fe^3+^ that are unavailable for intracellular use or ROS production.^91^ Ferritinophagy, a nuclear receptor coactivator 4 (NCOA4)-mediated autophagy-like process, can degrade ferritin, release iron stored in ferritin into the LIP and facilitate the availability of iron in cells, thereby boosting LPO-driven ferroptosis.^72,92–95^ Through its function as a selective autophagy receptor, NCOA4 transports intracellular ferritin to autophagic lysosomes and releases free iron through binding FTH1.^96^ Inhibition of cytosolic glutamate oxaloacetate transaminase 1, which enhances ferritinophagy, can increase the LIP and promote ferroptosis.^97^ Conversely, inhibition of ferritinophagy mediated by NCOA4 decreases the LIP and suppresses ferroptosis.^93,94^

### Ferroptosis defense systems

#### SLC7A11–GSH–GPX4 axis

There are four antiferroptosis defense systems (or cellular antioxidant systems) that directly neutralize lipid peroxides with distinctive subcellular localizations. Related to the metabolism of amino acids, the SLC7A11–GSH–GPX4 axis is a well-defined and major antiferroptosis defense system.^6,53^ SLC7A11 (also named xCT) and solute carrier family 3 member 2 constitute system Xc^−^.^98,99^ xCT is a transporter subunit and mediates the antiporter activity of system Xc^−^ through the import of extracellular cystine and export of intracellular glutamate.^98,100^ Through a reduction reaction mediated by nicotinamide adenine dinucleotide phosphate (NADPH) consumption in the cytosol, extracellular cystine taken up via SLC7A11 is rapidly reduced to cysteine, which then serves as the rate-limiting precursor for GSH biosynthesis.^99^ GSH is the major cofactor for GPX4-mediated detoxification of lipid peroxides.^99^ Inhibition of SLC7A11 activity or depletion of cystine promotes ferroptosis in various cancer cells.^99^ GPX4 is a member of the GPX protein family with enzymatic lipid repair activity,^101,102^ and it has been identified as a key ferroptosis inhibitor.^4,103,104^ GPX4 can promote the production of nontoxic lipid PL alcohols from PL hydroperoxides (L-OOH) and simultaneously oxidize two GSH molecules to yield oxidized GSH (GSSG).^46,105^ Accumulating studies have revealed the critical role of GPX4 in inhibiting ferroptosis through its genetic or pharmacological manipulation.^106,107^ GPX4 is regulated by epigenetic modifications and PTMs.^108^ Small molecule inhibitors of GPX4 could be optimized for use as anticancer agents.^109^

#### The FSP1–CoQH_2_ system

Several nonperoxidase mechanisms function in parallel with GPX4 to inhibit LPO and ferroptosis. FSP1, localized on the plasma membrane, is also known as apoptosis-inducing factor mitochondria-associated 2. In 2019, FSP1 was identified as the second main protein inhibiting ferroptosis independent of GPX4 through the production of coenzyme Q_10_ (CoQ_10_, also known as ubiquinone), reduced forms of endogenous electron carriers and vitamin K, all of which possess significant antioxidant (RTA) activity.^56,57,110^ FSP1 functions as an NAD(P)H-dependent oxidoreductase to reduce CoQ_10_ to regenerate CoQ_10_-H_2_, the reduced form of CoQ_10_, which can trap lipid peroxyl radicals to hinder LPO and halt ferroptosis.^56,57^ FSP1 also inhibits ferroptosis independently of its oxidoreductase function by activating ESCRT-III-dependent membrane repair, which halts ferroptosis.^111,112^ Small molecule inhibitors of FSP1 could also be optimized as anticancer agents.^57^

#### The GCH1–BH_4_ system

A study in 2020 revealed that the GCH1–BH4 system is another critical GPX4-independent inhibitor of ferroptosis that acts by suppressing LPO.^54,55^ GCH1, which mediates the rate-limiting reaction generating the endogenous metabolite BH_4_, was discovered as a suppressor by Kraft^54^ and as an enhancer of ferroptosis by Birsoy in 2020.^55^ As a different RTA, BH4 can be regenerated following its RTA reactions by dihydrofolate reductase (DHFR). Inhibition of DHFR synergizes with inhibition of GPX4 to induce ferroptosis.^55^ Moreover, BH4 enhances CoQ_10_ synthesis by converting phenylalanine into tyrosine, which can be further converted to 4-OH-benzoate, the precursor of CoQ_10_.^54^ GCH1-mediated BH4 synthesis reprograms lipid metabolism and inhibits ferroptosis by selectively preventing two polyunsaturated fatty acyl tails from depleting PLs.^54^

#### The DHODH–CoQH_2_ system

A newly identified GPX4-independent mitochondria-localized ferroptosis defense system, the DHODH–CoQH_2_ system can compensate for GPX4 loss and detoxify mitochondrial lipid peroxides.^58^ DHODH is localized to the inner mitochondrial membrane, where it catalyzes de novo pyrimidine synthesis through which CoQ10 can be reduced to CoQH_2_ at the rate-limiting fourth step. The function of CoQH_2_ is analogous to that of FSP1 in extra-mitochondrial membranes.^58^ After acute inactivation of GPX4, DHODH-mediated flux is significantly increased, leading to increased generation of CoQH_2_, which neutralizes LPO and inhibits ferroptosis in mitochondria.^58^ Inactivation of both mitochondrial DHODH and GPX4 causes robust ferroptosis by unleashing potent LPO reactions in mitochondria.^6^ Low expression of DHODH or high expression of GCH1 renders cells more sensitive or resistant to ferroptosis, respectively.

#### MBOAT1/2–MUFA system

The MBOAT1/2-PE-MUFA system is a newly identified ferroptosis defense mechanism independent of GPX4 and FSP1.^59^ Jiang and colleagues identified new PL-modifying enzymes, MBOAT1 and MBOAT2, which function as ferroptosis suppressors.^59^ By functioning as a lyso-PL acyltransferase (LPLAT), membrane-bound MBOAT2 inhibits ferroptosis by selectively incorporating MUFAs into lysophosphatidylethanolamine (lyso-PE), thereby correspondingly increasing the abundance of cellular PE-MUFAs and decreasing the abundance of cellular PE-PUFAs. PE-PUFAs are the preferred substrate for LPO and determine ferroptosis sensitivity.^73,74^ The sex hormone receptors, i.e., the estrogen receptor (ER) and androgen receptor (AR), directly transcriptionally upregulate MBOAT1 and MBOAT2, respectively. AR or ER antagonists boost the antitumor activity of ferroptosis inducers in AR^+^ prostate cancers and ER^+^ breast cancers with or without resistance to single-agent hormone therapies.^59^

## Epigenetic and posttranslational modifications

Epigenetic modification, a dynamic and reversible process, regulates gene expression without changing the DNA sequence.^9,10^ There are four major mechanisms of epigenetic regulation: DNA methylation, chromatin structure regulation, histone PTM, and ncRNA regulation.^9,10,113^ The common well-studied epigenetic regulatory mechanisms are DNA methylation, histone modification, and ncRNA regulation.^11^ The histone subunit in the nucleosome contains a characteristic tail possessing specific amino acids for covalent PTMs, such as ubiquitination, phosphorylation, methylation, acetylation, SUMOylation, acylation, glycosylation, hydroxylation, serotonylation, glycation, and ADP-ribosylation.^114–117^ Epigenetic regulation of gene expression is mediated by various classes of proteins, most of which have enzymatic activities. Four classes of epigenetic regulators, i.e., “writers”, “erasers”, “readers”, and “remodelers”, constitute the molecular component of the epigenetic regulators of DNA and histone modifications and chromatin structure.^113,118^ The writers and erasers add and remove epigenetic marks, respectively. The readers recognize specific epigenetic marks to mediate downstream effects, while the remodelers modulate the chromatin state.^10^ Approximately 1000 epigenetic regulators form one of the largest protein groups in mammals. Cancer develops as a result of progressive accumulation of cell-intrinsic genetic and epigenetic changes, which are key characteristics of most cancers.^119,120^ Epigenetic mechanisms regulate cancer biology in multiple ways, including driving tumorigenesis and invasion and modulating the immune response. Furthermore, modulation of the epigenome exposes cancer cells to immune-mediated attack, increasing cancer cell sensitivity to immunotherapy in various solid tumors.^121,122^ As covalent or enzymatic modifications of synthesized proteins, PTMs modify or introduce functional groups, such as phosphoryl, methyl, acetyl and glycosyl groups, to dynamically modulate protein localization, activity and molecular interactions.^12,13^ PTMs include phosphorylation, ubiquitination, SUMOylation, acetylation, methylation, ADP-ribosylation, palmitoylation, neddylation, glycosylation, prenylation, cholesterylation, myristoylation, glutathionylation, sulfhydration, citrullination, S-nitrosylation, and several novel PTMs.^13^ PTMs are usually reversible. Epigenetic modifications and PTMs are strongly correlated with the occurrence and genesis of many diseases. Epigenetic modifications and PTMs transcriptionally and posttranscriptionally regulate gene expression, respectively, and posttranscriptionally modulate protein activity, function and degradation.^123^ These modifications are required for the maintenance of tissue-specific expression of genes and proteins and for normal cellular development. Dysregulation of epigenetic modifications and PTMs causes aberrant expression of gene and protein signatures and transformation into malignant phenotypes, which induces disease onset and progression.^123–125^ Accumulating evidence has revealed that dysregulated epigenetic regulation contributes to tumor drug resistance, NSDs, CVDs, liver diseases, lung diseases, and kidney diseases.

## Epigenetic and posttranslational modifications regulating ferroptosis in diseases

### Epigenetic and posttranslational modifications regulating ferroptosis in cancer

#### Ubiquitination-mediated regulation of ferroptosis in cancer

**Fig. 3:**
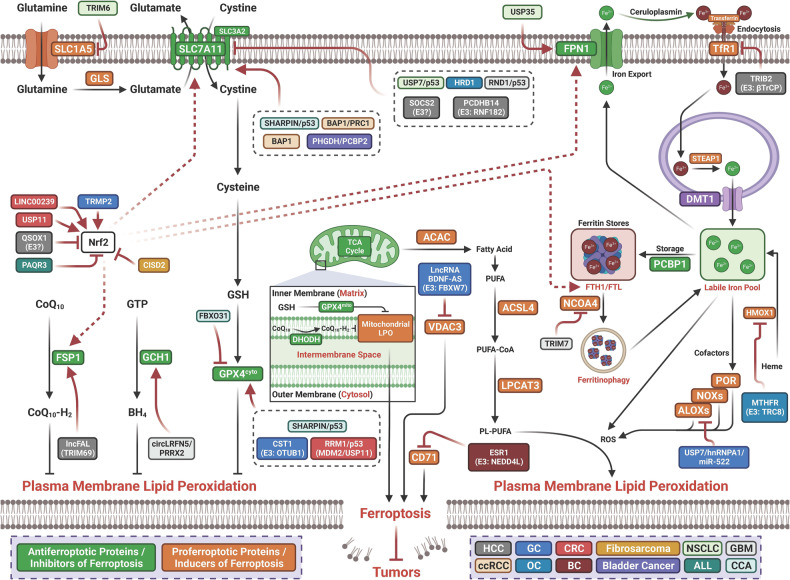
Posttranslational modification of ferroptosis by ubiquitination in cancer. ALL acute lymphoblastic leukemia; BAP1 tumor suppressor BRCA1-associated protein 1, BC breast cancer, CCA cholangiocarcinoma, CISD2 CDGSH iron sulfur domain 2, ccRCC clear cell renal cell carcinoma, CRC colorectal cancer, ESCC esophageal squamous cell carcinoma, GBC gallbladder cancer, GBM glioblastoma, GC gastric cancer, HCC hepatocellular carcinoma, HNRNPA2B1 heterogeneous nuclear ribonucleoprotein A2/B1, OC ovarian cancer, QSOX1 quiescin sulfhydryl oxidase 1, TEAD4 TEA domain family member 4, USP ubiquitin specific peptidase, RND1 Rho family GTPase 1, SOCS2 suppressor of cytokine signaling 2, βTrCP beta-transducin repeat containing E3 ubiqutin protein ligase

Ubiquitination is a key and highly conserved PTM and plays a vital role in modulating the degradation of various protein substrates.^126,127^ Deubiquitinases (DUBs) can remove ubiquitin chains to reverse ubiquitination, leading to termination of ubiquitination and preservation of substrate protein expression.^127^ The interaction between ubiquitination by ubiquitinases and DUBs plays an important role in controlling almost all aspects of biological activities. Emerging studies have revealed that deubiquitination/ubiquitination are involved in regulating ferroptosis in cancer. Specific regulators can modulate ferroptosis by regulating the ubiquitination of ferroptosis-related factors (Table 1 and Fig. 3).

**Table 1:** Posttranslational modification of ferroptosis by ubiquitination in cancer

Cancer	Modification	Targets	E3s	DUBs	Biological functions	Ref
HCC	Ubiquitination	SLC7A11	-	-	SOCS2-enhanced ubiquitination of SLC7A11 promotes ferroptosis and radiosensitization in HCC.	^128^
HCC	Ubiquitination	SLC7A11	RNF182	-	p53-induced increase of PCDHB14 downregulates the expression of SLC7A11 thereby promoting ferroptosis and is a novel tumor suppressor in HCC. PCDHB14 promoting E3 ubiquitin ligase RNF182-mediated ubiquitination of p65 to block p65 binding to the promoter of SLC7A11.	^129^
HCC	Ubiquitination	EGFR/Nrf2	-		QSOX1 promotes sorafenib-induced ferroptosis in HCC by driving ubiquitination-mediated degradation of EGFR, leading to suppression of Nrf2 activation.	^130^
HCC	Ubiquitination	TfRC	βTrCP	-	TRIB2 inhibit ferroptosis via βTrCP-mediated TfRC ubiquitiantion in liver cancer cells	^132^
HCC	Ubiquitination	FSP1	TRIM69		HDLBP-stabilized lncFAL inhibits ferroptosis vulnerability by diminishing Trim69-dependent FSP1 degradation in HCC.	^133^
GC	Ubiquitination	Nrf2	TRPM2	-	Silencing TRPM2 enhanced ferroptosis in gastric cancer cells through destabilizing HIF-1α and Nrf2 proteins.	^134^
GC	Ubiquitination	VDAC3	FBXW7	-	LncRNA BDNF-AS inhibit ferroptosis through recruiting WDR5 to transcriptionly upregulate FBXW7, thereby mediating ubiquitiantion-dependent degradation of VDAC3 and promoted the progression of GC.	^135^
GC	Ubiquitination	GPX4	OTUB1	-	CST1 promotes gastric cancer metastasis by inhibits ferroptosis through inhibiting OTUB1-mediated GPX4 ubiquitination and degradation.	^136^
GC	Ubiquitination	ALOX15	-	USP7	Cisplatin and paclitaxel promote miR-522 secretion from CAFs by activating USP7/hnRNPA1 axis, leading to ALOX15 suppression and ferroptosis in cancer cells, and ultimately result in decreased chemosensitivity.	^137^
CRC	Ubiquitination	Nrf2	-	-	LINC00239 inhibits ferroptosis in CRC by binding to Keap1 to stabilize Nrf2.	^138^
CRC	Ubiquitination	P53/GPX4	MDM2	USP11	RRM1 deficiency impairs the stability of p53 and sensitizes different types of cancer cells to ferroptosis by reducing GPX4 expression. Knockdown of RRM1 stimulates the binding of the MDM2 and p53 while inhibiting the binding of USP11 to p53, thereby increasing the ubiquitination of p53. The instability of p53 results in lower expression of p21, which causes ferroptosis and a decrease in cell survival time by inhibiting GPX4.	^139^
CCA	Ubiquitination	GPX4	-	-	FBXO31 as a tumor suppressor sensitizes CSC-like cells to CDDP by promoting ferroptosis and facilitating the proteasomal degradation of GPX4. functions as a tumor.	^140^
CCA	Ubiquitination	p53/SLC7A11/GPX4	-	-	SHARPIN promotes cell proliferation through inhibiting ferroptosis via promoting the ubiquitination and degradation of p53, and upregulating SLC7A11/GPX4.	^141^
NSCLC	Ubiquitination	SLC7A11	-	USP7	Erastin induce ferroptosis through decreasing the levels of H2Bub1 that epigenetically activates the expression of SLC7A11. p53 negatively regulates H2Bub1 levels by promoting the nuclear translocation of the deubiquitinase USP7. p53 decreases H2Bub1 occupancy on the SLC7A11 gene regulatory region and represses the expression of SLC7A11 during erastin treatment.	^142^
NSCLC	Ubiquitination	SLC1A5	TRIM6	-	TRIM6 directly interacted with SLC1A5 to promote its ubiquitination and degradation, thereby inhibiting glutamine import, glutaminolysis, lipid peroxidation, and ferroptosis.	^143^
NSCLC	Ubiquitination	FPN	-	USP35	USP35 directly interacted with ferroportin (FPN) and functioned as a deubiquitinase to maintain its protein stability. USP35 knockdown sensitized lung cancer cells to cisplatin and paclitaxel chemotherapy.	^144^
NSCLC	Ubiquitination	Nrf2	-	USP11	Elevated USP11 promote NSCLC cancer cell proliferation through inhibiting ferroptosis via deubiquitinates and stabilizes Nrf2.	^145^
GBM	Ubiquitination	NCOA4	TRIM7	-	Elevated expression of TRIM7 in human glioblastoma. Silenced TRIM7 suppressed growth through inducing ferroptosis, while TRIM7overexpression inhibited ferroptosis. TRIM7 directly bound to and ubiquitinated nuclear receptor coactivator 4 (NCOA4), thereby reducing NCOA4-mediated ferritinophagy and ferroptosis of human glioblastoma cells. Moreover, we found that TRIM7 deletion sensitized human glioblastoma cells to temozolomide therapy.	^146^
GBM	Ubiquitination	p53/SLC7A11	-	-	RND1 induce ferroptosis through interacting and de-ubiquitinating p53, thereby inhibiting SLC7A11 in GBM.	^147^
GBM	Ubiquitination	PRRX2/GCH1	-	-	Downregulated circLRFN5 promote malignancy through inhibiting ferroptosis in GBM. CircLRFN5 binds to PRRX2 protein and promotes its degradation via a ubiquitin-mediated proteasomal pathway, thereby transcriptionally upregulating GCH1 expression in GSCs, which is a ferroptosis suppressor	^148^
ccRCC	Ubiquitination	SLC7A11	BAP1	-	BAP1 decreases H2Aub occupancy on the SLC7A11 promoter and represses SLC7A11 expression in a deubiquitinating-dependent manner, and that BAP1 inhibits cystine uptake by repressing SLC7A11 expression, leading to elevated lipid peroxidation and ferroptosis.	^149^
ccRCC	Ubiquitination	SLC7A11	BAP1/PRC1		BAP1 promotes erastin-induced ferroptosis through repressing SLC7A11 expression. BAP1 decreases whereas PRC1 (a major H2Aub ubiquitin ligase) increases H2Aub binding on the SLC7A11 promoter, both BAP1 and PRC1 represses SLC7A11 expression.	^628^
OC	Ubiquitination	HMOX1	TRC8	-	MTHFR inhibits TRC8-mediated HMOX1 ubiquitination thereby blocking ferroptosis and promote the tumor cells growth.	^151^
OC	Ubiquitination	SLC7A11	HRD1	-	HRD1 functions as a tumor suppressor by facilitating ubiquitination-dependent SLC7A11 degradation in ovarian cancer.	^152^
BC	Ubiquitination	CD71	NEDD4L	-	Estrogen receptor 1 (ESR1) promote cancer through inhibiting ferroptosis in breast cancer cells via the NEDD4L-mediated ubiquitination and degradation of CD71.	^150^
Bladder Cancer	Ubiquitination	SLC7A11	-	-	PHGDH interact with PCBP2 to inhibit its ubiquitination degradation, upregulates SLC7A11 and thereby inhibits ferroptosis and promotes malignant progression.	^153^
ALL	Ubiquitination	Nrf2	-	-	PAQR3 inhibits proliferation and aggravates ferroptosis in acute lymphoblastic leukemia through increasing Nrf2 degradation.	^154^
ALL	Ubiquitination	VDAC3	FBXW7	-	Autophagy activation sensitized ALL cells to erastin-induced ferroptosis through inhibiting FBXW7-mediated ubiquitiantion-dependant degradation of VDAC3.	^155^
Melanoma	Ubiquitination	VDAC2/3	Nedd4	-	Nedd4 ubiquitylates VDAC2/3 to suppress erastin-induced ferroptosis in melanoma.	^156^
Fibrosarcoma	Ubiquitination	Nrf2	-	-	CISD2 knockdown promoted the degradation of autophagy adaptor p62 and resulted in an increased Keap1-mediated Nrf2 ubiquitination and subsequent degradation.	^157^

*ALL* acute lymphoblastic leukemia, *BAP1* tumor suppressor BRCA1-associated protein 1, *BC* breast cancer, CCA cholangiocarcinoma, *CISD2* CDGSH iron sulfur domain 2, *ccRCC* clear cell renal cell carcinoma, *CRC* colorectal cancer, *DUBs* deubiquitinases, *ESCC* esophageal squamous cell carcinoma, *GBC* gallbladder cancer, *GBM* glioblastoma, *GC* gastric cancer, *HCC* hepatocellular carcinoma, *HNRNPA2B1* heterogeneous nuclear ribonucleoprotein A2/B1, *OC* ovarian cancer, *QSOX1* quiescin sulfhydryl oxidase 1, *TEAD4* TEA domain family member 4, *USP* ubiquitin specific peptidase, *RND1* Rho family GTPase 1, *SOCS2* suppressor of cytokine signaling 2, *βTrCP* beta-transducin repeat containing E3 ubiqutin protein ligase

Hepatocellular carcinoma (HCC): Suppressor of cytokine signaling 2-mediated ubiquitination of SLC7A11 enhances ferroptosis and radiosensitization in HCC.^128^ PCDHB14 functions as a novel tumor suppressor by enhancing ferroptosis through promotion of RNF182-dependent ubiquitination of p65 and blockade of its binding to the promoter of SLC7A11, thereby downregulating SLC7A11 in HCC.^129^ P53 binds to PCDHB14 to induce its expression.^129^ Quiescin sulfhydryl oxidase 1 (QSOX1) promotes ferroptosis induced by sorafenib in HCC by driving ubiquitin-mediated degradation of EGFR, leading to suppression of Nrf2 activation, suggesting that QSOX1 is an inducer of ferroptosis.^130^ Tribbles homolog 2 (TRIB2) functions as one of the key molecules that stabilizes GPX4 and attenuates oxidative stress-induced cell damage.^131^ A recent study revealed that TRIB2 inhibits and desensitizes ferroptosis through βTrCP-mediated ubiquitination of TfRC, thereby leading to a decline in the LIP in liver cancer cells.^132^ The largest RNA-binding protein, high-density lipoprotein-binding protein (HDLBP), is an important transporter that protects cells against cholesterol overaccumulation. Elevated expression of HDLBP stabilizes lncFAL to decrease ferroptosis vulnerability by diminishing TRIM69-mediated FSP1 degradation in HCC cells.^133^ Inhibition of FSP1 enhances the antitumor activity of ferroptosis inducers, supporting the potential utility of targeting FSP1 as a therapeutic approach for HCC patients with high HDLBP or lncFAL expression.^133^

Gastric cancer (GC): Erastin and RSL3 upregulate the cation channel transient receptor potential melastatin-2 (TRPM2) in GC cell lines. TRPM2 knockdown induces ferroptosis in GC cells, as evidenced by the reductions in the GSH content and GPX activity and increased concentrations of Fe^2+^, ROS and lipid peroxides. Silencing TRPM2 increases the sensitivity of GC cells to RSL3- and erastin-induced ferroptosis by destabilizing the HIF-1α and Nrf2 proteins, suggesting that TRPM2 functions as a negative regulator of erastin- and RSL3-induced ferroptosis^134^ and indicating that the combination of TRPM2-targeted drugs with chemotherapeutics potentiates the effectiveness of treatment and improves the outcome of patients. Increased expression of the long noncoding RNA (lncRNA) BDNF-AS inhibits ferroptosis by recruiting WDR5 to transcriptionally upregulate FBXW7, thereby mediating the ubiquitin-dependent degradation of VDAC3 and promoting the progression of GC.^135^ Increased expression of CST1 promotes the progression and metastasis of GC via inhibition of ferroptosis by reducing GPX4 ubiquitination and degradation by recruiting OTUB1.^136^ Paclitaxel and cisplatin (CDDP) increase the secretion of cancer-associated fibroblast-derived miR-522 by activating the USP7/hnRNPA1 axis, thereby suppressing ALOX15 expression and ferroptosis and ultimately leading to reduced chemosensitivity.^137^

Colorectal cancer (CRC): Increased expression of LINC00239 is associated with poorer prognosis in patients with CRC.^138^ Overexpression of LINC00239 inhibits erastin- and RSL3-mediated antitumor activity by inhibiting ferroptosis. LINC00239 interacts with and binds to the Kelch domain of Keap1, thereby inhibiting the ubiquitination of Nrf2 to stabilize it, suggesting that LINC00239 functions as an oncogene by inhibiting ferroptosis by binding to Keap1 and stabilizing Nrf2 in CRC cells.^138^ Loss of ribonucleotide reductase subunit M1 (RRM1) destabilizes p53 and increases the sensitivity of different types of cancer cells to ferroptosis by inhibiting the expression of GPX4. Silencing RRM1 promotes the interaction of MDM2 and P53 while decreasing the binding of USP11 to p53, thereby stimulating the ubiquitination of p53, in turn leading to decreased expression of p21, which eventually induces ferroptosis through inhibition of GPX4 and results in cancer cell death.^139^

Cholangiocarcinoma (CCA): FBXO31 functions as a tumor suppressor, and its expression increases the sensitivity of stem cell-like cells to cisplatin by enhancing ferroptosis through promotion of the proteasomal degradation of GPX4 in CCA cells.^140^ The expression of a component of the linear ubiquitin chain activation complex, shank-associated RH domain interacting protein (SHARPIN), was found to be increased, promoting cell proliferation through ferroptosis inhibition mediated by promoting the ubiquitination and degradation of P53, thereby upregulating SLC7A11/GPX in CCA cells.^141^ Blocking SHARPIN-mediated inhibition of ferroptosis via the P53/SLC7A11/GPX4 axis and targeting SHARPIN might be promising treatment approaches for CCA.

Non-small cell lung carcinoma (NSCLC): Inducing ferroptosis is a good treatment approach for LUAD patients with late-stage and/or therapy-resistant tumors. Erastin promotes the nuclear translocation of USP7 by increasing its interaction with p53, which erases the monoubiquitination of lysine 120 on histone H2B (H2Bub1) in the SLC7A11 gene regulatory region and inactivates SCL7A11 expression, eventually leading to ferroptosis in NSCLC cells.^142^ TRIM6 functions as an oncogene by promoting SLC1A5 ubiquitination and degradation, thereby inhibiting glutamine import, glutaminolysis, LPO, and ferroptosis.^143^ USP35 is upregulated in NSCLC. USP35 knockdown promotes ferroptosis and increases the sensitivity of lung cancer cells to paclitaxel and cisplatin.^144^ Conversely, overexpression of USP35 facilitates lung cancer cell growth and tumor progression by reducing Erastin/RSL3-triggered ferroptosis. USP35 directly interacts with the FPN protein to maintain its stability, suggesting that USP35 functions as an oncogene by inhibiting ferroptosis through stabilization of FPN.^144^ USP11 deubiquitinates and stabilizes Nrf2. Elevated USP11 expression promotes cancer cell proliferation by inhibiting ferroptosis through deubiquitination and stabilization of Nrf2 in NSCLC cells.^145^

Glioblastoma: Overexpression of TRIM7 inhibits NCOA4-mediated ferritinophagy and ferroptosis through directly binding and ubiquitinating NCOA4 in human glioblastoma cells. Ablation of TRIM7 increases the sensitivity of human glioblastoma cells to temozolomide, suggesting that TRIM7 functions as a negative regulator of ferroptosis.^146^ Downregulated expression of Rho family GTPase 1 (RND1) predicts a better prognosis in patients with glioblastoma multiforme (GBM). RND1 induces ferroptosis by interacting with and deubiquitinating P53, thereby inhibiting SLC7A11 in GBM cells.^147^ Circular RNAs (circRNAs) regulate ferroptosis through several mechanisms in GBM. Downregulation of circLRFN5 promotes malignancy by inhibiting ferroptosis in GBM cells. CircLRFN5 binds to the paired related homeobox 2 (PRRX2) protein and promotes its ubiquitin-mediated degradation, thereby transcriptionally upregulating the expression of the ferroptosis suppressor GCH1 in glioma stem cells (GSCs), leading to ferroptosis induction.^148^

Clear cell renal cell carcinoma (ccRCC): As a tumor suppressor, the H2A DUB BRCA1-associated protein 1 (BAP1) suppresses tumorigenesis by inducing ferroptosis through suppression of SLC7A11 in ccRCC. BAP1, which encodes a nuclear DUB to reduce histone 2A ubiquitination (H2Aub) on chromatin, reduces the H2Aub level in the SLC7A11 promoter and suppresses SLC7A11 expression in a deubiquitination-dependent manner, leading to inhibition of cystine uptake, LPO and ferroptosis.^149^ BAP1 inhibits the progression of tumors partially by inducing ferroptosis through suppression of SLC7A11 expression, and cancer-associated BAP1 mutants lose their ability to suppress SLC7A11 and promote ferroptosis. BAP1 promotes Erastin-induced ferroptosis by repressing SLC7A11 expression. BAP1 and PRC1 (a major H2Aub ligase) coordinately suppress SLC7A11 expression by regulating the level of H2Aub in the SLC7A11 promoter.^150^

Gynecologic neoplasms: An increased expression level of estrogen receptor 1 (ESR1) promotes cancer by inhibiting ferroptosis in breast cancer cells through NEDD4L-mediated ubiquitination and degradation of CD71.^150^ Silencing ESR1 significantly promotes ionizing radiation-mediated ferroptosis and increases the CD71 protein level.^150^ The results suggest that in breast cancer, ESR1 is an inhibitor of ferroptosis, while CD71 is an inducer of ferroptosis.^150^ Methylenetetrahydrofolate reductase (MTHFR), a key enzyme for folic acid metabolism, inhibits TRC8-mediated HMOX1 ubiquitination, thereby blocking ferroptosis and promoting the growth of ovarian cancer (OC) cells.^151^ The E3 ubiquitin ligase 3-hydroxy-3-methylglutaryl reductase (HRD1) exhibits decreased degradation in ovarian cancer tissues and functions as a tumor suppressor. HRD1 was found to inhibit the proliferation and colony formation of ovarian cancer cells by inducing ferroptosis through facilitation of ubiquitination-dependent SLC7A11 degradation. This finding suggests that HRD1 exerts antitumor effects by promoting ferroptosis in ovarian cancer cells by increasing SLC7A11 degradation.^152^

Other tumors: In bladder cancer (BCa), an important serine metabolism enzyme, phosphoglycerate dehydrogenase (PHGDH), is highly expressed. PHGDH interacts with the RNA-binding protein poly(rC)-binding Protein 2 and inhibits its ubiquitin-mediated degradation, which in turn upregulates SLC7A11 and inhibits ferroptosis, thereby promoting malignant progression.^153^ The PHGDH inhibitor NCT-502 enhances ferroptosis and halts tumor progression in BCa.^153^ This finding indicates that inhibition of PHGDH could be a therapeutic strategy for BCa. PAQR3, a member of the Progestin and AdipoQ Receptor (PAQR) family, is a newly discovered tumor suppressor, and its expression is decreased in acute lymphoblastic leukemia (ALL). PAQR3 suppresses cell proliferation and aggravates ferroptosis by increasing ubiquitin-dependent degradation of Nrf2 in ALL cells.^154^ Activation of autophagy was found to sensitize ALL cells to Erastin-induced ferroptosis by inhibiting ubiquitination-dependent degradation of VDAC3 mediated by the E3 ligase FBXW7,^155^ indicating that autophagy activation combined with ferroptosis induction is a potential therapeutic strategy for ALL. FOXM1 and Nedd4 regulate VDAC2/3 during ferroptosis in melanoma cells.^156^ Erastin induces FOXM1 expression to activate the transcription of Nedd4, which degrades VDAC2/3 and suppresses ferroptosis. Ablation of Nedd4 inhibits the degradation of VDAC2/3 proteins, increasing the sensitivity of cancer cells to Erastin-induced ferroptosis.^156^ These results suggest that Nedd4 regulates ferroptosis and highlight Nedd4 as a target for overcoming Erastin-induced resistance in melanoma cells. Silencing CDGSH iron sulfur domain 2, an iron-sulfur protein with a [2Fe-2S] cluster that is critical for cell proliferation and iron homeostasis, increases the degradation of p62 (an autophagy adaptor), leading to increased Keap1-mediated ubiquitination of Nrf2 and its subsequent degradation, thereby promoting ferroptosis in fibrosarcoma cells.^157^

#### Phosphorylation-mediated regulation of ferroptosis in cancer

**Fig. 4:**
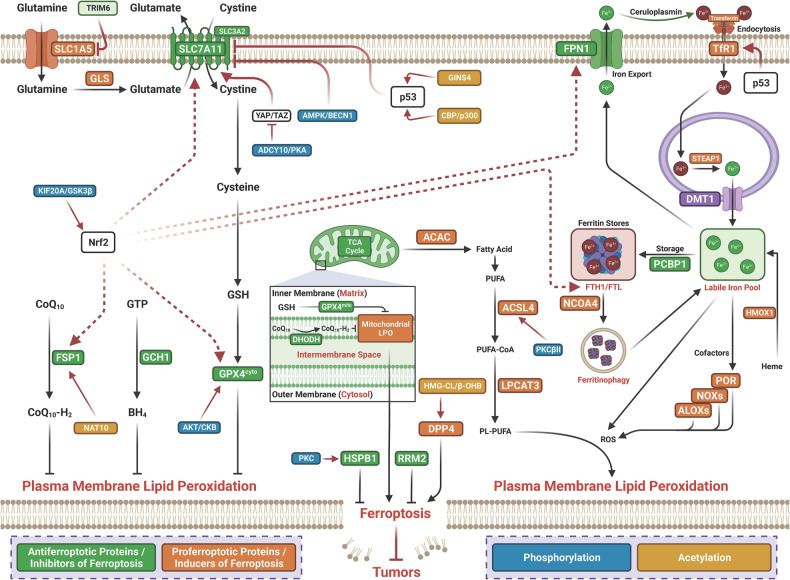
Posttranslational modification of ferroptosis by phosphorylation and acetylation in cancer. ATF4 Activating transcription factor 4, ADCY10 adenylyl cyclase 10; β-OHB β-hydroxy-butyric acid; βTrCP beta-transducin repeat containing E3 ubiqutin protein ligase, CBP/p300 histone acetyltransferases CBP and p300, CCA cholangiocarcinoma, CRC colorectal cancer, CHAC1 glutathione specific gamma-glutamylcyclotransferase 1, CISD2 CDGSH iron sulfur domain 2, CKB creatine kinase B, DDR2 discoidin domain receptor tyrosine kinase 2, eIF2α alpha subunit of eukaryotic initiation factor 2, FSP1 ferroptosis suppressor protein 1, GC gastric cancer, GCN2 general control nonderepressible 2, GSS glutathione synthetase, HCC hepatocellular carcinoma, HMGCL ketogenesis-related hydroxy-methyl-glutaryl-CoA lyase, HSPB1 heat shock protein beta-1, HSF1 heat shock factor 1, IGF1R insulin-like growth factor 1 receptor, LUAD lung adenocarcinoma, NAT10 N-acetyltransferase 10, PKC protein kinase C, MESH1 metazoan SpoT homolog 1, RRM2 ribonucleotide reductase regulatory subunit M2, RND1 Rho family GTPase 1, TNBC, triple-negative breast cancer, YAP Yes-associated protein

Histone phosphorylation is a histone modification whose modulation is catalyzed by many protein kinases and phosphatases, such as protein phosphatase 1, mitogen- and stress-activated kinases, and Aurora B, and is achieved through the addition of phosphate groups to threonine, serine or tyrosine residues in histone tails.^158^ Histone phosphorylation frequently occurs early after the formation of a DNA double-strand break and mediates the recruitment of DNA damage repair proteins.^159^ Histone phosphorylation has been revealed to be associated with transcriptional activation. As an important epigenetic PTM, phosphorylation is strongly associated with tumorigenesis.^160,161^ Emerging studies suggest that phosphorylation regulates ferroptosis in cancer (Table 2 and Fig. 4).

**Table 2:** Posttranslational modification of ferroptosis by phosphorylation in cancer

Cancer	Modification	Targets	Enzyme	Biological functions	Ref.
CRC	Phosphorylation	BECN1	AMPK	AMPK-mediated BECN1 phosphorylation promotes ferroptosis by directly blocking system Xc– activity.	^162^
CRC	Phosphorylation	Nrf2	GSK3β	KIF20A was highly expressed in the oxaliplatin-resistant cell lines. Silencing KIF20A enhanced cellular sensitivity to oxaliplatin, and suppressed NUAK1, thereby upregulating the expression of PP1β, down-regulating the phosphorylation of downstream GSK3β to suppressed activation of Nrf2 and the expression of GPX4, and blocked cellular resistance.	^163^
HCC	Phosphorylation	GPX4	AKT/CKB	IGF1R activated AKT phosphorylates CKB at T133, reduces metabolic activity of CKB and increases CKB binding to and phosphorylates GPX4 at S104, which prevents HSC70 binding to GPX4, thereby abrogating the GPX4 degradation regulated by chaperone-mediated autophagy, alleviating ferroptosis and promoting tumor growth in mice.	^164^
HCC	Phosphorylation	RRM2	-	Elevated RRM2 inhibited ferroptosis. Phosphorylation of RRM2 was maintained at normal levels to block the RRM2-GSS interaction and therefore protected RRM2 and GSS from further proteasome degradation. However, under ferroptotic stress, RRM2 was dephosphorylated at T33, thus the RRM2-GSS interaction was promoted. This resulted in the translocation of RRM2 and GSS to the proteasome for simultaneous degradation.	^165^
GC	Phosphorylation	eIF2α	-	MESH1 knockdown upregulate ATF3 and ATF4 protein, eIF2α phosphorylation, and induction of ATF3, XBPs, and CHOP mRNA. Concurrent ATF4 knockdown re-sensitizes MESH1-depleted RCC4 cells to ferroptosis. ATF3 induction is abolished by the concurrent knockdown of NADK, implicating a role of NADPH accumulation in the integrative stress response.	^167^
Breast cancer	Phosphorylation	ACSL4	PKCβII	PKCβII phosphorylates ACSL4 to amplify lipid peroxidation to induce ferroptosis.	^77^
Breast cancer	Phosphorylation	DDR2	-	Erastin treatment induces DDR2 upregulation and phosphorylation. EMT-driven DDR2 upregulation in recurrent tumors in maintaining growth advantage but activating YAP/TAZ-mediated ferroptosis susceptibility.	^168^
TNBC	Phosphorylation	eIF2α	-	Cystine starvation activate GCN2 to increase the phosphorylation of eIF2α, the protein expression of ATF4, and CHAC1. Knockdown of CHAC1 rescued the cystine-starvation-induced ferroptosis.	^169^
NSCLC	Phosphorylation	YAP	PKA	Inhibition of system XC^-^ increase endogenous glutamate accumulation, by which promotes Ca^2+^-dependent cAMP production by ADCY10 to stimulate PKA-associated phosphorylation and suppression of GFPT1. Subsequently, YAP is inevitably suppressed and fail to sustain ferritinophagy-triggered transcriptional compensatory of FTH1, leading to a varied labile iron elevation and ferroptosis sensitivity.	^170^
Osteosarcoma/prostate adenocarcinoma	Phosphorylation	HSPB1	PKC	Knockdown of HSF1 and HSPB1 enhances erastin-induced ferroptosis, whereas heat shock pretreatment and overexpression of HSPB1 inhibits erastin-induced ferroptosis. PKC-mediated HSPB1 phosphorylation confers protection against ferroptosis. Moreover, inhibition of the HSF1-HSPB1 pathway and HSPB1 phosphorylation increases the anticancer activity of erastin in human xenograft mouse tumor models.	^171^

*ATF4* Activating transcription factor 4, *ADCY10* adenylyl cyclase 10, *CCA* cholangiocarcinoma, *CRC* colorectal cancer, *CHAC1* glutathione specific gamma-glutamylcyclotransferase 1, *CISD2* CDGSH iron sulfur domain 2, *CKB* creatine kinase B, *DDR2* discoidin domain receptor tyrosine kinase 2, *eIF2α* alpha subunit of eukaryotic initiation factor 2, *GC* gastric cancer; *GCN2* general control nonderepressible 2, *GSS* glutathione synthetase, *HCC* hepatocellular carcinoma, *HSPB1* heat shock protein beta-1, *HSF1* heat shock factor 1, *IGF1R* insulin-like growth factor 1 receptor, *PKC* protein kinase C, *MESH1* metazoan SpoT homolog 1, *RRM2* ribonucleotide reductase regulatory subunit M2, *RND1* Rho family GTPase 1, *TNBC* triple negative breast cancer, *βTrCP* beta-transducin repeat containing E3 ubiqutin protein ligase, *YAP* Yes-associated protein

Colorectal cancer: AMP-activated protein kinase (AMPK)-mediated phosphorylation of BECN1 enhances ferroptosis through binding to SLC7A11 to directly block system Xc^–^.^162^ Silencing BECN1 inhibits ferroptosis induced by the system Xc^-^ inhibitors erastin-, sulfasalazine-, and sorafenib. Phosphorylation of BECN1 (Ser90/93/96) induced by AMPK is required for BECN1-SLC7A11 complex formation and LPO. Inhibition of PRKAA/AMPKα reduces Erastin-mediated BECN1 phosphorylation at S93/96, BECN1-SLC7A11 complex formation, and subsequent ferroptosis. Activation of the BECN1 pathway increases ferroptosis in CRC cells.^162^ KIF20A expression is increased in oxaliplatin-resistant CRC cell lines. Silencing KIF20A increases the sensitivity of cancer cells to oxaliplatin and suppresses NUAK1, thereby upregulating the expression of PP1β, which subsequently decreases the phosphorylation of downstream GSK3β to suppress the activation of Nrf2 and the expression of GPX4, abolishing oxaliplatin resistance in these cells.^163^

Hepatocellular carcinoma: AKT activated by insulin-like growth factor 1 receptor (IGF1R) signaling phosphorylates creatine kinase B (CKB) at T133, reducing its metabolic activity and increasing its binding to and phosphorylation of GPX4 at S104, which prevents HSC70 binding to GPX4, thereby abrogating degradation of GPX4 by chaperone-mediated autophagy, inhibiting ferroptosis and promoting tumor growth in mice.^164^ Elevated expression of ribonucleotide reductase regulatory subunit M2 (RRM2) inhibits ferroptosis in HCC cells. Ferroptotic stress induces phosphorylation of RRM2 at T33, thus promoting the RRM2-GSS interaction, which results in the translocation of RRM2 and GSS to the proteasome for simultaneous degradation.^165^

Gastric cancer: A recent study showed that Metazoan SpoT Homolog 1 (MESH1) is the first NADPH phosphatase regulating ferroptosis in the cytosol.^166^ Silencing MESH1 dramatically protects cells against ferroptosis. In GC, MESH1 knockdown upregulates the protein expression of ATF3 and ATF4, increases eIF2α phosphorylation, and induces the mRNA expression of XBPs, ATF3, and CHOP. Concurrent silencing of ATF4 restores the sensitivity of MESH1-depleted RCC4 cells to ferroptosis. Concurrent knockdown of NADK abolishes ATF3 induction.^167^

Breast cancer: PKCβII phosphorylates ACSL4 to boost LPO to induce ferroptosis.^77^ PKCβII was found to function as a critical contributor to ferroptosis by sensing initial lipid peroxides and amplifying ferroptosis-associated LPO by phosphorylating and activating ACSL4. Inhibiting the PKCβII-ACSL4 pathway attenuates ferroptosis in vitro and impedes immunotherapy-induced ferroptosis in vivo. Murine recurrent breast tumor cells are highly sensitive to ferroptosis. The receptor for collagen I, discoidin domain receptor tyrosine kinase 2 (DDR2), is upregulated in human mesenchymal breast cancer cells and ferroptosis-sensitive recurrent tumor cells. Upregulation of DDR2 increases the susceptibility of recurrent breast tumors to ferroptosis through the Hippo pathway.^168^ Erastin induces upregulation and phosphorylation of DDR2. Epithelial–mesenchymal transition (EMT)-driven DDR2 upregulation maintains a growth advantage but results in ferroptosis susceptibility mediated by YAP/TAZ in recurrent tumors.^168^ Silencing DDR2 reduces the clonogenic proliferation of recurrent tumor cells. These results reveal an important role of EMT-driven DDR2 upregulation in maintaining the growth advantage but endowing YAP/TAZ-mediated ferroptosis susceptibility in recurrent tumors, highlighting potential therapeutic strategies to eradicate recurrent breast cancer cells with mesenchymal features.^168^ Cystine starvation activates GCN2 to increase the phosphorylation of eIF2α and the expression of the ATF4 protein and its target gene CHAC1. Silencing CHAC1 rescues cystine depletion-induced ferroptosis in human triple-negative breast cancer (TNBC) cells.^169^

Non-small cell lung carcinoma: Inhibition of system XC^−^ increases endogenous glutamate accumulation, which enhances adenylyl cyclase (ADCY)-mediated Ca^2+^-dependent cAMP production to stimulate protein kinase A (PKA)-associated phosphorylation and suppress glutamine-fructose-6-phosphate transaminase (GFPT1), thereby suppressing YAP expression and failing to sustain ferritinophagy-triggered transcriptional compensation of FTH1, leading to increases in the LIP and ferroptosis sensitivity.^170^

Osteosarcoma/prostate adenocarcinoma: Heat shock protein beta-1 (HSPB1) functions as an inhibitor of ferroptosis in cancer. Erastin enhances heat shock factor 1 (HSF1)-dependent HSPB1 expression in cancer cells. Silencing HSPB1 and HSF1 promotes but HSPB1 overexpression inhibits erastin-induced ferroptosis. Protein kinase C (PKC)-mediated HSPB1 phosphorylation inhibits ferroptosis. Inhibition of HSPB1 phosphorylation and HSF1-HSPB1 signaling boosts the anticancer activity of erastin in vivo.^171^

#### Acetylation-mediated regulation of ferroptosis in cancer

Protein acetylation is required for key cellular processes related to physiology and diseases, such as transcriptional activity, protein stability, enzyme activity, protein‒protein interactions, subcellular localization, and protein‒DNA interactions.^172^ Histone acetylation was first found to modulate gene transcription as early as the 1960s.^173^ Since the first acetylation modification of a nonhistone protein, p53, was found in the 1980s, various nonhistone proteins have been identified as targets for acetylation.^172^ Emerging studies suggest that acetylation regulates ferroptosis in cancer (Table 3 and Fig. 4).

**Table 3:** Posttranslational modification of ferroptosis by acetylation in cancer

Cancer	Modification	Targets	Enzyme	Biological functions	Ref
NSCLC	Acetylation	p53	CBP/p300	Acetylation is crucial for p53-mediated ferroptosis and tumor suppression.	^175^
NSCLC	Acetylation	p53	-	p53 inhibits cystine uptake and sensitizes cells to ferroptosis by repressing expression of SLC7A11. Notably, p53,^3KR^ an acetylation-defective mutant that fails to induce cell-cycle arrest, senescence and apoptosis, fully retains the ability to regulate SLC7A11 expression and induce ferroptosis upon ROS-induced stress.	^174^
NSCLC	Acetylation	p53	-	GINS4 negatively regulate ferroptosis in LUAD. GINS4 suppressed p53-mediated ferroptosis through stabilizing p53 via activate snail that antagonized the acetylation of p53(K351).	^176^
HCC	Acetylation	β-OHB	-	HMGCL increased H3K9 acetylation through β-OHB and promoting the expression of DPP4 in a dose-dependent manner, leading to HCC cells vulnerability to erastin- and sorafenib-induced ferroptosis.	^177^
Glioma	Acetylation	STAT3	KAT6B	KAT6B contributes to glioma progression by repressing ferroptosis via epigenetically inducing STAT3.	^179^
Osteosarcoma	Acetylation	p53		mTOR inhibition acts as an unexpected checkpoint in p53-mediated tumor suppression.	^180^
CRC	Acetylation	FSP1	NAT10	NAT10 promotes colon cancer progression by inhibiting ferroptosis through N4-acetylation and stabilization of FSP1 mRNA.	^178^

*CBP/p300* histone acetyltransferases CBP and p300, *HMGCL* ketogenesis-related hydroxy-methyl-glutaryl-CoA lyase, *β-OHB* β-hydroxy-butyric acid, *NAT10* N-acetyltransferase 10, *FSP1* ferroptosis suppressor protein 1, *LUAD* lung adenocarcinoma

Non-small cell lung carcinoma: Regulation of ferroptosis by P53 was first reported in 2015, and the associated study revealed SLC7A11 as a direct target gene of P53 for suppression.^174^ The acetylation-defective mutant p53^3KR^ was found to be unable to induce cell cycle arrest, senescence and apoptosis but retained the full ability to inhibit SLC7A11 expression and induce ferroptosis. Gu and colleagues revealed the role of acetylation in modulating P53-mediated ferroptosis and tumor suppression in NSCLC.^175^ Expression of P53^3KR^ efficiently inhibited tumor growth, which was restored by the overexpression of SLC7A11 in vivo, suggesting the important role of SLC7A11 inhibition in p53-mediated tumor suppression. However, p53^4KR^ (K98R + 3KR) lost the ability to suppress SLC7A11, thus inducing ferroptosis and tumor suppression.^175^ These results revealed the role of acetylation in regulating p53-mediated ferroptosis and tumor suppression. Most recent studies have shown that GINS4, a regulator of initiation and elongation during DNA replication, negatively regulates ferroptosis in lung adenocarcinoma (LUAD). Ablation of GINS4 facilitates ferroptosis.^176^ GINS4 suppresses P53-mediated ferroptosis by stabilizing p53 via activation of snail, which antagonizes the acetylation of P53 at K351. These results indicate that GINS4 is a potential oncogene that destabilizes p53 and then inhibits ferroptosis, thus constituting a potential therapeutic target for LUAD.^176^

Hepatocellular carcinoma: Dipeptidyl peptidase 4 (DPP4) is a key protein that maintains intracellular iron accumulation and LPO. Ketogenesis-related hydroxy-methyl-glutaryl-CoA lyase (HMGCL) negatively regulates cell proliferation and metastasis in HCC. HMGCL increases β-hydroxybutyric acid (β-OHB)-mediated acetylation of DPP4 at histone 3 lysine 9 (H3K9) and promotes its expression, leading to increased vulnerability of HCC cells to erastin- and sorafenib-induced ferroptosis.^177^ This observation suggests that HMGCL functions as a tumor suppressor by increasing ferroptosis susceptibility driven by β-OHB-mediated acetylation of DPP4.

Colorectal cancer: NAT10 negatively regulates tumorigenesis and metastasis in CRC. Upregulation of N-acetyltransferase 10 (NAT10) promotes cancer progression by inhibiting ferroptosis via N4 acetylation and stabilization of FSP1 mRNA in CRC cells.^178^ FSP1 mRNA undergoes N4-acetylcytidine (ac4C) modification, leading to inhibition of ferroptosis. This observation reveals that NAT10-mediated N4 acetylation of FSP1 mRNA terminates ferroptosis in colon cancer cells.

Glioma: KAT6B, a histone acetyltransferase, promotes glioma progression by inhibiting ferroptosis through epigenetic induction of STAT3.^179^ KAT6B expression is increased in glioma. KAT6B reverses erastin-induced ferroptosis in glioma cells, indicating that KAT6B functions as an inhibitor of ferroptosis. A mechanistic study showed that ablation of KAT6B represses the expression of STAT3. Silencing KAT6B inhibits the enrichment of RNA polymerase II (RNA pol II) and histone H3 lysine 23 acetylation (H3K23ac) on the STAT3 promoter, while loss of STAT3 reverses KAT6B-induced inhibition of ferroptosis in glioma cells.

Osteosarcoma: K139 has been identified as a novel acetylation site in human p53 accounting for p53-mediated mTOR suppression.^180^ The p53-4KR mutant retains the ability to inhibit mTOR activity, which is completely abolished in the p53-5KR (K136R + K98R + K117R + K161R + K162R) mutant. The 5KR mutation series in p53 abolishes its remaining tumor suppressor function. Treatment with an mTOR inhibitor was found to suppress early-onset tumor formation in P535KR/5KR mice, which was similar to that observed in p53-null mice. This finding reveals a role of p53-mediated mTOR regulation in tumor suppression.^180^

#### Methylation-mediated regulation of ferroptosis in cancer

**Fig. 5:**
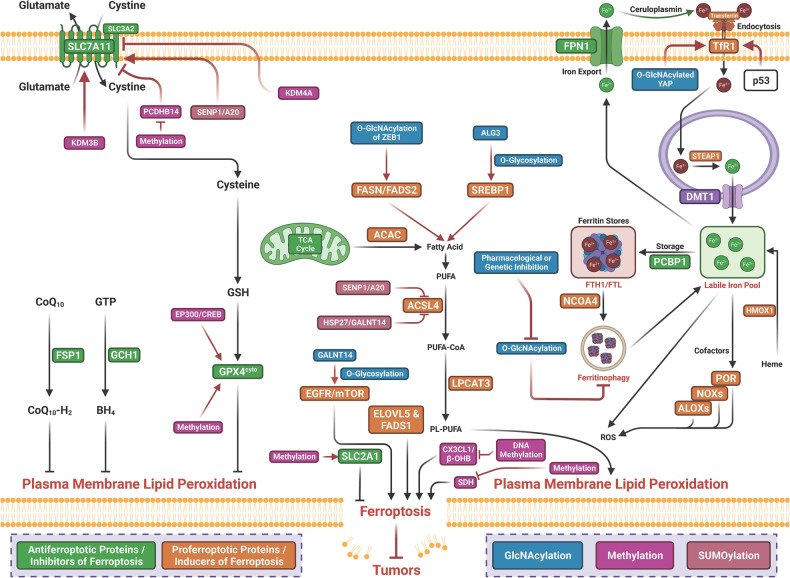
Epigenetic modification of ferroptosis by methylation, glcNAcylation, and SUMOylation in cancer. ALG3 Alpha 1,3-mannosyltransferase, CBS cystathionine -beta-synthase, CREB, cAMP response element-binding protein, CYP2E1 cytochrome P450 family two subfamily E member 1, EGFR epidermal growth factor receptor, ELOVL5 elongation of very long-chain fatty acid protein 5, EP300 E1A binding protein P300, FADS2 fatty acid desaturase 2, FASN fatty acid synthase, FADS1 fatty acid desaturase 1, FSP1 ferroptosis suppressor protein 1, FTH ferritin heavy chain, GALNT14 N-Acetylgalactosaminyltransferase-14, GBM glioblastoma, HMGCL ketogenesis-related hydroxy-methyl-glutaryl-CoA lyase, KDM3B histone lysine demethylase 3B, KDM4A histone lysine demethylase 4 A, β-OHB β-hydroxy-butyric acid, MM multiple myeloma, NAT10 N-acetyltransferase 10, PSAT1 phosphoserine aminotransferases 1, SDH succinate dehydrogenase, SENP1 small ubiquitin-like modifier (SUMO)-specific protease 1, TFRC transferrin receptor

First discovered in 1959, protein methylation is an important PTM that regulates the functions of both nonhistone and histone proteins.^181,182^ Histon methylation was identified in 1964.^183^ Currently, accumulating discoveries have revealed much of the biology of protein methylation.^184^ Protein methylation occurs mainly on side chains of arginine (Arg) and lysine (Lys) residues.^185^ The protein Arg methyltransferases (PRMTs) that use S-adenosylmethionine (SAM) as the methyl donor induce mono- or dimethylation of Arg on its side chains,^185,186^ whereas Lys residues may undergo mono-, di- or trimethylation (me1, me2 or me3, respectively) in a SAM-dependent manner.^187^ Circumstantial evidence has shown that dysregulation of protein methylation is involved in tumorigenesis.^188,189^ Emerging studies have suggested that methylation regulates ferroptosis in cancer (Table 4 and Fig. 5).

**Table 4:** Epigenetic modification of ferroptosis by methylation in cancer

Cancer	Modification	Targets	Enzyme	Biological functions	Ref
GC	Methylation	ELOVL5 and FADS1	-	The expression of elongation of ELOVL5 and FADS1 is up-regulated in mesenchymal-type gastric cancer cells (GCs), leading to ferroptosis sensitization. In contrast, these enzymes are silenced by DNA methylation in intestinal-type GCs, rendering cells resistant to ferroptosis. Intestinal-type GCs are unable to generate arachidonic acid (AA) and adrenic acid (AdA) from linoleic acid. AA supplementation of intestinal-type GCs restores their sensitivity to ferroptosis.	^190^
CRC	Methylation	SLC2A1	-	Increased methylation levels of SLC2A1 were greatly, inhibited autophagy and ferroptosisis correlated with the immunosuppression, resulting in a poor prognosis for patients.	^191^
HCC	Methylation	PCDHB14	-	PCDHB14 is inactivated by aberrant methylation of its promoter in HCC patients and that PCDHB14 functions as a tumor suppressor to promote cell cycle arrest, inhibit cell proliferation, and induce ferroptosis. PCDHB14, a novel gene induced by p53 activation, significantly enhances RNF182-mediated degradation of p65 to inhibit HCC progression and promote cell sensitivity to ferroptosis by suppressing SLC7A11.	^129^
NSCLC	Methylation	GPX4	-	Upstream of GPX4 there was low DNA methylation sites and enhanced level of H3K4me3 and H3K27ac lead to increase GPX4. Inhibition of tumor GPX4 induces ferroptosis in cancer cells and enhances anticancer effect of cisplatin.	^192^
NSCLC	Methylation	GPX4	EP300	Knockdown of CREB inhibited cell viability and growth by promoting ferroptosis. CREB suppressed ferroptosis by binding the promoter region of GPX4, and this binding could be enhanced by EP300.	^193^
ccRCC	Methylation	β-OHB	-	CX3CL1 overexpression inhibited tumor cell proliferation and metastasis and promoted tumor ferroptosis sensitivity in ccRCC.The expression of CX3CL1 in ccRCC is correlated with its DNA methylation level.	^195^
ccRCC	Methylation	SDH	-	Increased methylation and high SDH promoter mutation rates lead to deficiency of SDH, thereby promoting tumorigenesis through weakening of ferroptosis.	^196^
ALL	Methylation	FSP1	-	The promoter of the gene coding for FSP1 is hypermethylated in ALL, silencing the expression of FSP1 and creating a selective dependency on GSH-centered anti-ferroptosis defenses. In-trans expression of FSP1 increases the resistance of leukemic cells to compounds targeting the GSH-dependent anti-ferroptosis pathway. FSP1 over-expression promotes ALL-tumor growth.	^197^
MM	Methylation	ND	-	Ferroptosis induction leads to DNA methylation and histone modification changes associated with cellular senescence	^198^
Fibrosarcoma	Methylation	SLC7A11	KDM3B	Histone demethylase KDM3B results in decreased histone H3 lysine 9 methylation and protects against ferroptosis by upregulating SLC7A11 through cooperation with the transcription factor ATF4.	^199^
Osteosarcoma	Methylation	SLC7A11	KDM4A	Upregulated KDM4A was associated with poorer prognosis. KDM4A knockdown promoted ferroptosis through regulating SLC7A11 transcription by controlling H3K9me3 demethylation in the promoter region of SLC7A11.	^200^

*ALL* acute lymphoblastic leukemia, *CREB* cAMP response element-binding protein, *CYP2E1* cytochrome P450 family two subfamily E member 1, *ELOVL5* elongation of very long-chain fatty acid protein 5, *EP300* E1A binding protein P300, *FADS1* fatty acid desaturase 1, *FSP1* ferroptosis suppressor protein 1, *HMGCL* ketogenesis-related hydroxy-methyl-glutaryl-CoA lyase, *KDM3B* histone lysine demethylase 3B, *KDM4A* histone lysine demethylase 4A, *β-OHB* β-hydroxy-butyric acid, *NAT10* N-acetyltransferase 10, *MM* multiple myeloma, *SDH* succinate dehydrogenase

##### Cancers of the digestive system

Increased expression of ELOVL5 and FADS1 enhances the sensitivity of mesenchymal-type GC cells to ferroptosis. ELOVL5 and FADS1 are silenced by DNA methylation in intestinal-type GCs, rendering cells resistant to ferroptosis. AA and AdA derived from linoleic acid are not generated in intestinal-type GCs. AA supplementation restores the ferroptosis sensitivity of intestinal-type GCs.^190^ In CRC, increased methylation levels of solute carrier family 2 member 1 (SLC2A1) greatly inhibit autophagy and ferroptosis, resulting in immunosuppression and a poor prognosis in patients.^191^ Aberrant methylation of the promoter inactivates PCDHB14 in HCC patients. PCDHB14 functions as a tumor suppressor to enhance cell cycle arrest, inhibit cell proliferation, and induce ferroptosis. PCDHB14 inhibits HCC progression by enhancing RNF182-mediated degradation of p65 and promotes cell sensitivity to ferroptosis in HCC by suppressing SLC7A11.^129^

##### Non-small cell lung carcinoma

There are low levels of DNA methylation in upstream regions of GPX4 and an increased level of H3K4me3. H3K27ac leads to increased expression of GPX4. Inhibiting the expression of GPX4 induces ferroptosis in cancer cells and boosts the anticancer effect of cisplatin.^192^ cAMP response element-binding protein (CREB) is upregulated in LUAD. Silencing CREB decreases the viability and inhibits the growth of cancer cells by promoting ferroptosis. CREB suppresses ferroptosis by binding the promoter region of GPX4, and E1A binding protein P300 (EP300) enhances this binding.^193^ A lysine monomethylase, SET7, directly interacts with the DUB OTUB1 to catalyze its methylation at lysine 122, inhibiting the binding of OTUB1 to the E2 ubiquitin-conjugating enzyme UBC13. SET7-mediated methylation of OTUB1 promotes ferroptosis through relieving OTUB1-mediated suppression of ferroptosis, highlighting that SET7 inhibitor treatment might enhance OTUB1 function as a therapeutic approach.^194^

##### Clear cell renal cell carcinoma

CX3CL1 overexpression attenuates the proliferation and metastasis of ccRCC cells by increasing their sensitivity to ferroptosis. The expression of CX3CL1 is associated with its DNA methylation level in ccRCC.^195^ Decreased expression of succinate dehydrogenase (SDH), which is responsible for oxidative phosphorylation (OXPHOS) and flux through the tricarboxylic acid (TCA) cycle, is correlated with ccRCC progression. SDH deficiency enhances tumorigenesis by inhibiting ferroptosis in ccRCC cells. High mutation rates and increased methylation in the SDH promoter lead to SDH deficiency, thereby promoting tumorigenesis through inhibition of ferroptosis.^196^

The promoter of the gene coding for FSP1 is hypermethylated in ALL cells, silencing the expression of FSP1 and generating selective dependency on GSH-centered antiferroptosis defenses. Expression of FSP1 in trans increases the resistance of leukemic cells to compounds targeting the GSH-dependent antiferroptosis pathway. FSP1 overexpression promotes ALL tumor growth.

##### Acute lymphoblastic leukemia

ALL cells are selectively sensitive to compounds that block the GSH-dependent ferroptosis defense system. The promoter of *FSP1* has been found to be hypermethylated in ALL cell lines and patient biopsies. Silencing FSP1 produces selective dependency on the GSH-centered ferroptosis defense system. Overexpression of FSP1 enhances the resistance of leukemic cells to compounds that target the GSH-dependent antiferroptosis pathway, revealing metabolic vulnerability in ALL.^197^

Ferroptosis induction leads to changes in histone modification and DNA methylation associated with cellular senescence.

##### Multiple myeloma

RSL3-induced ferroptosis leads to changes in histone modification and DNA methylation related to cellular senescence.^198^ MM1 multiple myeloma cells are sensitive to ferroptosis induced by RSL3 and epigenetic reprogramming. Enrichment of CpG probes in genes associated with cell cycle progression and senescence was found in ferroptotic MM cells, suggesting that ferroptotic cell death is associated with an epigenomic stress response that might increase the therapeutic applicability of ferroptotic compounds.^198^

##### Fibrosarcoma

The H3K9 demethylase KDM3B functions as a potential epigenetic regulator of ferroptosis. KDM3B can inhibit erastin-triggered ferroptosis. Overexpression of KDM3B reduces H3K9 methylation and inhibits ferroptosis by upregulating SLC7A11 in cooperation with the transcription factor ATF4.^199^

##### Osteosarcoma

Upregulated KDM4A expression was found to be associated with poorer prognosis. Knockdown of KDM4A promotes ferroptosis through regulation of SLC7A11 transcription by controlling the demethylation of H3K9me3 in the SLC7A11 promoter.^200^

#### Glycosylation-mediated regulation of ferroptosis in cancer

O-GlcNAcylation, the attachment of O-linked N-acetylglucosamine (O-GlcNAc) moieties to threonine or serine residues of proteins in the nucleus, cytosol or mitochondria, is an important PTM that links nutrient flux to gene transcription in tumorigenesis.^201,202^ O-GlcNAcylation is dynamically and finely modulated by O-GlcNAcase (OGA) and O-GlcNAc transferase (OGT) proteins. Abnormal O-GlcNAcylation has been identified as a common characteristic in cancers due to deregulated cellular nutrient flux.^203,204^ Emerging studies suggest that GlcNAcylation regulates ferroptosis in cancer (Table 5 and Fig. 5).

**Table 5:** Posttranslational modification of Ferroptosis by GlcNAcylation in cancer

Cancer	Modification	Targets	Enzyme	Biological functions	Ref
HCC	O-GlcNAcylation	c-Jun	-	O-GlcNAcylated c-Jun antagonizes ferroptosis via inhibiting GSH synthesis in liver cancer. Erastin specifically inhibited c-Jun O-GlcNAcylation in liver cancer and further suppressed the related cancer-promoting function of c-Jun. Overexpression of O-GlcNAcylated c-Jun conversely repressed ferroptosis via stimulating GSH synthesis through boosting the transcription of PSAT1 and CBS.	^205^
HCC	O-GlcNAcylation	YAP	-	O-GlcNAcylated YAP mediates the ferroptosis sensitivity through transcriptional elevation of TFRC in HCC cells	^206^
Pancreatic cancer	O-GlcNAcylation	ZEB1	-	O-GlcNAcylation of ZEB1 facilitated mesenchymal pancreatic cancer cell ferroptosis. High glucose increased O-GlcNAcylation of ZEB1, transcriptionly inducing FASN and FADS2, thereby resulting in ferroptosisin mesenchymal pancreatic cancer cells.	^207^
OC	O-GlcNAcylation	EGFR	GALNT14	GALNT14 is significantly upregulated in ovarian cancer. Downregulation of GALNT14 significantly inhibits both apoptosis and ferroptosis of ovarian cancer cells. Downregulation of GALNT14 suppresses the activity of the mTOR pathway through modifying O-glycosylation of EGFR. Finally, an additive effect promoting cell death occurs with a combination of an mTOR inhibitor and cisplatin.	^208^
BC	N-GlcNAcylation	SREBP1	ALG3	Inhibition of ALG3 lead to N-linked glycosylation deficiency-mediated ferroptosis to boost anti-PD1 immunotherapy.	^209^
Osteosarcoma	O-GlcNAcylation	FTH	-	Inhibition of O-GlcNAcylation promoted ferritinophagy, resulting in the accumulation of labile iron and rendering the cell more sensitive to ferroptosis. de-O-GlcNAcylation of the FTH at S179 promoted its interaction with NCOA4, the ferritinophagy receptor, thereby accumulating labile iron for ferroptosis.	^210^

*ALG3* alpha 1,3-mannosyltransferase, *BC* breast cancer, *CBS* cystathionine-beta-synthase, *EGFR* epidermal growth factor receptor, *FADS2* fatty acid desaturase 2, *FASN* fatty acid synthase, *GALNT14* N-Acetylgalactosaminyltransferase-14, *FTH* ferritin heavy chain, *OC* Ovarian cancer, *PSAT1* phosphoserine aminotransferases 1, *TFRC* transferrin receptor

Cancers of the digestive system: GlcNAcylated c-Jun represses ferroptosis by antagonizing the synthesis of GSH in HCC. Erastin inhibits malignant phenotypes by inhibiting the O-GlcNAcylation, protein expression, transcriptional activity and nuclear accumulation of c-Jun in HCC. An overabundance of O-GlcNAcylated c-Jun conversely inhibits ferroptosis by increasing the synthesis of GSH via increased transcription of PSAT1 and CBS.^205^ This observation indicates that O-GlcNAcylated c-Jun is at the core of ferroptosis and that targeting c-Jun O-GlcNAcylation might be a potential therapeutic approach for HCC.^205^ O-GlcNAcylation stabilizes and enhances the expression of YAP, which plays a pivotal role in controlling ferroptosis. O-GlcNAcylated YAP mediates increased ferroptosis sensitivity through a transcriptional increase in TfRC expression in HCC cells.^206^ Knockdown or mutation of YAP abolishes the O-GlcNAcylation-mediated increase in sensitivity to ferroptosis. The related study provided the first evidence that O-GlcNAcylation can increase the sensitivity of HCC cells to ferroptosis via YAP/TFRC, highlighting new therapeutic strategies for HCC.^206^ In pancreatic cancer, O-GlcNAcylation of zinc finger E-box-binding homeobox 1 (ZEB1) enhances ferroptosis in mesenchymal pancreatic cancer cells. High glucose exposure increases the O-GlcNAcylation of ZEB1, transcriptionally inducing fatty acid synthase (FASN) and fatty acid desaturase 2 (FADS2) expression, thereby resulting in ferroptosis in mesenchymal pancreatic cancer cells.^207^ These results indicate that glycolipid metabolism and O-GlcNAcylation play a novel role in increasing ferroptosis susceptibility in mesenchymal cancer cells, which reveals a new molecular mechanism of ferroptosis and suggests a therapeutic strategy for refractory pancreatic cancers.

Gynecologic neoplasms: Increased expression of GALNT14 is found in ovarian cancer. Silencing GALNT14 eliminates ovarian cancer cells by promoting apoptosis and ferroptosis by reducing the O-glycosylation of EGFR and promoting its degradation, thereby suppressing mTOR pathway activity.^208^ Combination treatment with an mTOR inhibitor and cisplatin induced apoptosis and ferroptosis, suggesting that the combination of cisplatin with an mTOR inhibitor might be a promising strategy to combat cisplatin resistance in ovarian cancer.^208^ Alpha 1,3-mannosyltransferase (ALG3) is involved in protein glycosylation critical for the assembly of lipid-linked oligosaccharides and in N-linked glycosylation of proteins at the luminal side of the endoplasmic reticulum (ER). Inhibition of ALG3 leads to N-linked glycosylation deficiency-mediated ferroptosis to boost the efficacy of anti-PD1 immunotherapy.^209^ Ablation of ALG3 was found to attenuate tumor growth in a cytotoxic T-cell-dependent manner in mice.^209^ Moreover, ALG3 inhibition and treatment with tunicamycin (an N-linked glycosylation inhibitor) synergize with anti-PD1 therapy to inhibit tumor growth in mouse models.^209^ Inhibition of ALG3 induces impairment of posttranslational N-linked glycosylation, resulting in sterol-regulated element-binding protein (SREBP1)-dependent lipogenesis and excessive lipid accumulation, which induces immunogenic ferroptosis in cancer cells and leads to the formation of a proinflammatory microenvironment, thereby boosting antitumor immune responses.

Osteosarcoma: Protein O-GlcNAcylation orchestrates both mitophagy and ferritinophagy to support ferroptosis in osteosarcoma cells.^210^ RSL3 modulates ferroptosis by inducing a biphasic change in protein O-GlcNAcylation. Inhibition of O-GlcNAcylation enhances ferritinophagy, leading to an increased labile iron content, thus rendering the cell more sensitive to ferroptosis. De-O-GlcNAcylation of FTH at S179 facilitates its interaction with NCOA4, resulting in labile iron accumulation to support ferroptosis.^210^ These results reveal links between dynamic O-GlcNAcylation and both iron metabolism and ferroptosis initiation, highlighting a potential therapeutic regimen for cancers.

#### SUMOylation-mediated regulation of ferroptosis in cancer

A PTM involving conjugation of small ubiquitin-like modifier (SUMO) proteins to substrate proteins, SUMOylation is involved in various cellular processes and is a vital cellular mechanism in stress responses.^211^ SUMOylation is also a reversible and dynamic process. SUMOylation occurs through an enzymatic cascade that consists of a dimeric SUMO-activating E1 enzyme (SAE1 and SAE2/UBA2), a single E2 enzyme (ubiquitin-conjugating enzyme 9, UBC9), and a limited set of E3 ligases.^212^ SUMO-specific proteases (SENPs) cooperate with SUMO molecules to modulate the SUMOylation status of a substrate protein by specifically de-SUMOylating the substrate protein. Aberrantly overactivated SUMOylation has been detected in many cancers and is involved in EMT, tumorigenesis, metastasis, drug resistance, and antitumor immunity.^211,213^ Emerging studies suggest that SUMOylation regulates ferroptosis in cancer (Table 6 and Fig. 5). Aberrantly increased SENP1 expression predicts a poor prognosis in patients with lung cancer. Inhibition of SENP1 inhibits the proliferation and growth of lung cancer cells. Overexpression of SENP1 inhibits Erastin- or cisplatin-induced ferroptosis. Elevated SENP1 expression mediates A20 SUMOylation to reduce the expression of ACSL4 and induce the expression of SLC7A11, thereby inhibiting ferroptosis in lung cancers.^214^ SENP1 functions as a ferroptosis suppressor, as determined through a novel network analysis of how A20 SUMOylation links SLC7A11 and ACSL4 in lung cancer cells. Inhibition of SENP1 enhances ferroptosis. Elevated HSP27 expression inhibits ferroptosis by inducing SUMOylation of ACSL4 to reduce its stability in GBM cells.^215^

**Table 6:** Posttranslational modification of Ferroptosis by SUMOylation in cancer

Cancer	Modification	Targets	Enzyme	Biological functions	Ref
Lung cancer	SUMOylation	A20	SENP1	Elevated SENP1 mediated A20 SUMOylation lead to decreased ACSL4 and increased SLC7A11, thereby inhibiting ferroptosis.	^214^
GBM	SUMOylation	ACSL4	GALNT14	Elevated HSP27 inhibit ferroptosis through inducing SUMOylation of ACSL4 to reduce its stability in GBM.	^215^

*GBM* glioblastoma, *SENP1* Small ubiquitin-like modifier (SUMO)-specific protease 1

#### N_6_-methyladenosine (m^6^A) modification-mediated regulation of ferroptosis in cancer

**Fig. 6:**
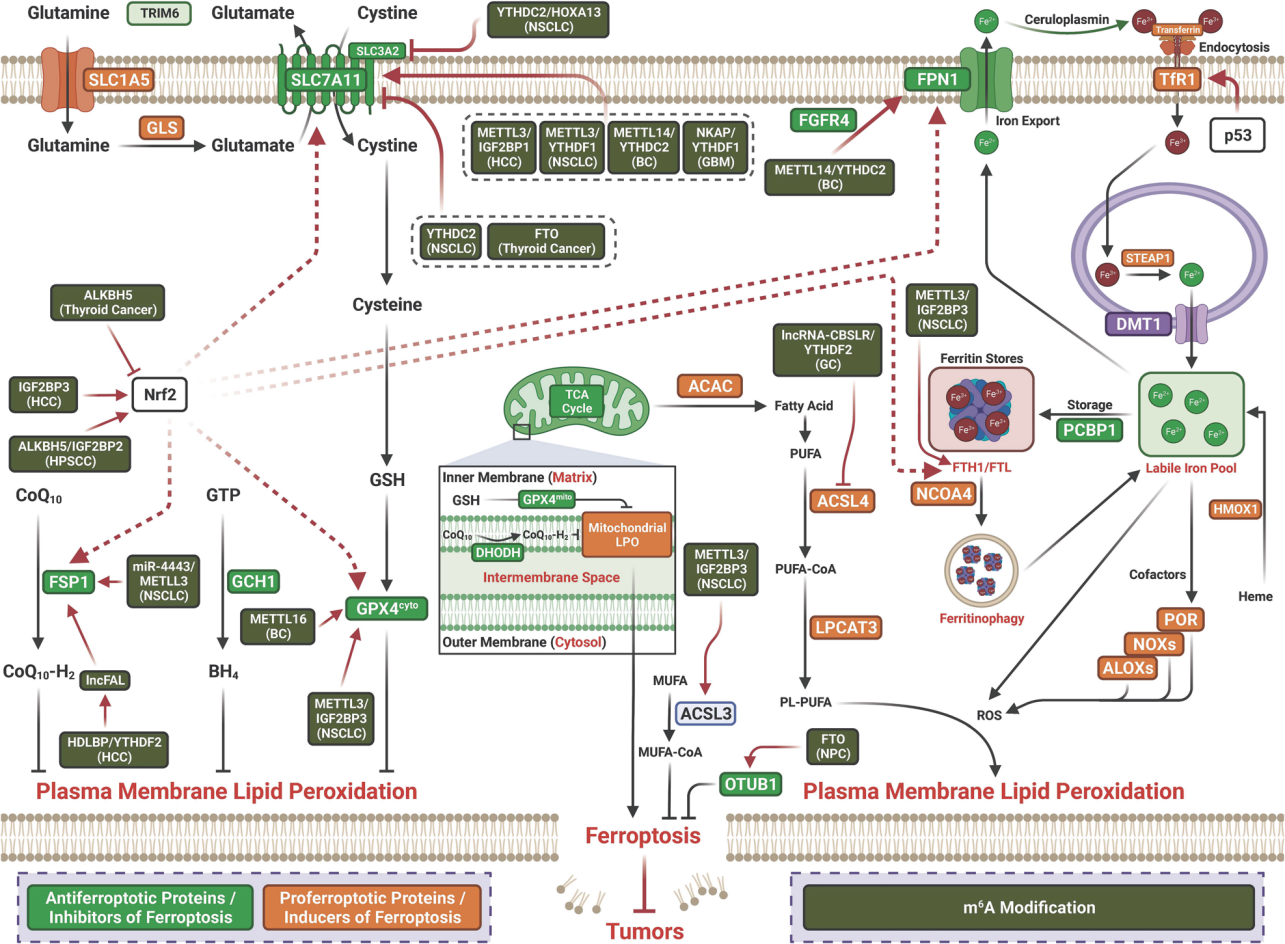
Epigenetic modification of ferroptosis by m^6^A in cancer. ALKBH5 AlkB homolog 5, ACSL3 acyl-CoA synthetase long-chain family member 3, BC breast cancer, CBS cystathionine-beta-synthase, FSP1 ferroptosis suppressor protein 1, FTH1 ferritin heavy chain 1, FTO fat mass and obesity-associated protein, GBM glioblastoma, GC gastric cancer, HCC hepatocellular carcinoma, GPX4 glutathione peroxidase 4, HDLBP high-density lipoprotein-binding protein, HPSCC hypopharyngeal squamous cell carcinoma, IGF2BP1 Insulin-like growth factor 2 mRNA binding protein 1, IGF2BP2 Insulin-like growth factor 2 mRNA binding protein 2, METTL3 methyltransferase-like protein 3, METTL14 methyltransferase -like 14, METTL16 methyltransferase-like 16, NKAP NF-κB activating protein, NPC nasopharyngeal carcinoma, NSCLC non-small cell lung carcinoma, SFPQ splicing factor proline and glutamine-rich, SLC3A2 solute carrier family 3 member 2, YTHDC2 YTH domain containing 2, YTHDF1 YTH N6-methyladenosine RNA binding protein 1, YTHDF2 YTH N6-methyladenosine RNA binding protein 2, YTHDC2 YTH domain containing 2

Accumulating studies have shown that m^6^A modification plays a vital role in epigenetic regulation in organisms and in the pathogenesis of malignant diseases. Aberrant m^6^A modification, a dynamic and reversible posttranscriptional RNA modification mediated by methyltransferases (writers), demethylases (erasers), and m^6^A binding proteins (readers), is associated with the development, progression, occurrence, and prognosis of cancer. Emerging studies suggest that m^6^A modification regulates ferroptosis in cancer (Table 7 and Fig. 6).

**Table 7:** Epigenetic modification of Ferroptosis by m^6^A in cancer

Cancer	Modification	Targets	Writer	Eraser	Reader	Biological functions	Ref
NSCLC	m^6^A	FSP1	METLL3	-	-	Exosomal miR-4443 promotes cisplatin resistance by enhancing METLL3-mediated m^6^A modification of FSP1, thereby inhibiting ferroptosis	^216^
NSCLC	m^6^A	SLC7A11	-	-	YTHDC2	YTHDC2 inhibit LUAD tumorigenesis by suppressing SLC7A11 mRNA and promoting its decay in an m^6^A-dependent manner	^217^
NSCLC	m^6^A	HOXA13			YTHDC2	YTHDC2 promote m^6^A methylation and subsequent destabilization of HOXA13 mRNA to suppress SLC3A2, thereby inducing ferroptosis and inhibiting LUAD tumorigenesis	^218^
NSCLC	m^6^A	GPX4;SLC3A2;FTH1;ACSL3	METTL3	-	IGF2BP3	Upregulated IGF2BP3 recognizes and binds target mRNAs encoding anti-ferroptosis factors that can be m^6^A-methylated by METTL3, leading to suppress ferroptosis and stimulate tumorigenesis.	^219^
NSCLC	m^6^A	SLC7A11	METTL3	-	YTHDF1	METTL3 promotes lung adenocarcinoma tumor growth and inhibits ferroptosis by stabilizing SLC7A11 m^6^A modifcation	^220^
HCC	m^6^A	FSP1	-	-	YTHDF2	Elevated HDLBP inhibited the ferroptosis. HDLBP bound to and stabilized the lncFAL. The splicing of lncFAL was increased by YTHDF2 in a m^6^A-dependent manner. lncFAL reduced ferroptosis vulnerability by directly binding to FSP1 and competitively abolishing Trim69-dependent FSP1 polyubiquitination degradation.	^133^
HCC	m^6^A	Nrf2	-	-	IGF2BP3	Upregulation of IGF2BP3 inhibit sorafenib-induced ferroptosisis through promoting Nrf2 mRNA stability in an m^6^A-dependent manner	^221^
HCC	m^6^A	SLC7A11	METTL3	-	IGF2BP1	METTL3-mediated SLC7A11 m^6^A modification enhances HB ferroptosis resistance. The METTL3/IGF2BP1/m^6^A modification promotes SLC7A11 mRNA stability and upregulates its expression by inhibiting the deadenylation process.	^222^
Thyroid cancer	m^6^A	TIAM1/Nrf2	-	ALKBH5	-	ALKBH5 inhibits thyroid cancer progression by promoting ferroptosis through inactivating Nrf2 by decreasing the m^6^A level of TIAM1 expression through m^6^A modification.	^223^
Thyroid cancer	m^6^A	SLC7A11	-	FTO	-	FTO prevents thyroid cancer progression by downregulating SLC7A11 by m^6^A methylation in a ferroptosis-dependent manner	^224^
BC	m^6^A	GPX4	METTL16	-	-	METTL16 epigenetically enhances GPX4 expression via m^6^A modification to promote breast cancer progression by inhibiting ferroptosis	^225^
BC	m^6^A	FGFR4	METTL14		YTHDC2	m^6^A modification level is reduced due to the downregulation of METTL14 in anti-HER2 resistant breast cancer. Decrease of m^6^A level prevents the YTHDC2 mediated FGFR4 mRNA degradation, therefore, lead to the accumulation of FGFR4 in resistant breast cancer. FGFR4 phosphorylates GSK-3β and activates β-catenin/TCF4 signaling to increase the transcription of the SLC7A11 and FPN1 gene. Upregulated SLC7A11 and FPN1 accelerates glutathione synthesis and Fe^2+^ efflux, which confer anti-HER2 resistance by attenuating ferroptosis in breast cancer. Roblitinib, a highly selective inhibitor of FGFR4, overcomes anti-HER2 resistance by triggering ferroptosis in recalcitrant HER2-positive breast cancer.	^226^
GBM	m^6^A	SLC7A11	METTL3	-	-	NKAP inhibit ferroptosis by recruiting SFPQ to promote SLC7A11 mRNA splicing in an METTL3-mediated m^6^A-dependent manner.	^227^
NPC	m^6^A	OTUB1	-	FTO	-	Upregulated FTO enhances radioresistance by repressing radiation-induced ferroptosis in NPC. FTO acts as an m^6^A demethylase to erase the m^6^A modification of the OTUB1 transcript and promote the expression of OTUB1, thereby inhibiting the ferroptosis.	^228^
HPSCC	m^6^A	Nrf2	-	ALKBH5	IGF2BP2	ALKBH5 inhibits ferroptosis by posttranscriptionally activating Nrf2 in an m^6^A-IGF2BP2-dependent manner.	^229^
GC	m^6^A	CBS	-	-	YTHDF2	HIF-1α induces lncRNA-CBSLR to recruit YTHDF2 protein and CBS mRNA to form CBSLR/ YTHDF2/CBS complex, which in turn decreases CBS mRNA stability in an m^6^A dependent manner, leading to reduce methylation of ACSL4 protein, thus, the protein is degraded via the ubiquitination-proteasome pathway, thereby inhibit ferroptosis.	^230^

*ALKBH5* AlkB homolog 5, *ACSL3* acyl-CoA synthetase long-chain family member 3, *BC* breast cancer, *CBS* cystathionine-beta-synthase, *FSP1* ferroptosis suppressor protein 1, *FTH1* ferritin heavy chain 1, *FTO* Fat mass and obesity-associated protein, *GBM* glioblastoma, *GC* gastric cancer, *HCC* hepatocellular carcinoma, *GPX4* glutathione peroxidase 4, *HDLBP* High-density lipoprotein-binding protein, *HPSCC* hypopharyngeal squamous cell carcinoma, *IGF2BP1* Insulin-like growth factor 2 mRNA binding protein 1, *IGF2BP2* Insulin-like growth factor 2 mRNA binding protein 2, *METTL3* methyltransferase-like protein 3, *METTL14* methyltransferase-like 14, *METTL16* methyltransferase-like 16, *NKAP* NF-κB activating protein, *NPC* nasopharyngeal carcinoma, *NSCLC* Non-small cell lung carcinoma SFPQ splicing factor proline and glutamine-rich, *SLC3A2* solute carrier family 3 member 2, *YTHDC2* YTH domain containing 2, *YTHDF1* YTH N6-methyladenosine RNA binding protein 1, *YTHDF2* YTH N6-methyladenosine RNA binding protein 2, *YTHDC2* YTH domain containing 2

Non-small cell lung carcinoma: Exosomal miR-4443 promotes cisplatin resistance by enhancing METLL3-mediated m^6^A modification of FSP1, thereby inhibiting ferroptosis.^216^ Downregulation of the m^6^A reader YT521-B homology domain containing 2 (YTHDC2) is related to poor clinical outcomes in patients with LUAD. YTHDC2 was found to inhibit tumorigenesis in a mouse model of spontaneous LUAD. YTHDC2 inhibits cystine uptake and blocks the downstream antioxidant system. Lung tumorigenesis is rescued by supplementation with downstream cystine antioxidants in mice with pulmonary YTHDC2 overexpression. YTHDC2 inhibits LUAD tumorigenesis by suppressing the transcription of SLC7A11 mRNA and promoting its decay in an m^6^A-dependent manner,^217^ suggesting that increased cystine uptake through suppression of YTHDC2 is critical for LUAD tumorigenesis and that blocking this process may be a therapeutic approach. This observation was corroborated by other studies, which showed that YTHDC2 promoted m^6^A methylation and subsequently destabilized HOXA13 mRNA to suppress SLC3A2 expression, thereby inducing ferroptosis and inhibiting LUAD tumorigenesis.^218^ The upregulated reader protein IGF2BP3 recognizes and binds target mRNAs encoding antiferroptotic factors that can undergo METTL3-mediated m^6^A methylation, leading to suppression of ferroptosis and stimulation of tumorigenesis.^219^ The writer METTL3 promotes tumor growth and inhibits ferroptosis by stabilizing the m^6^A modification of SLC7A11 in LUAD.^220^

Hepatocellular carcinoma: Elevated expression of HDLBP, the largest RNA-binding protein, inhibits ferroptosis in HCC cells. HDLBP binds to and stabilizes lncFAL. The splicing of lncFAL is enhanced by YTHDF2 in an m^6^A-dependent manner. lncFAL reduces vulnerability to ferroptosis by directly binding to FSP1 and competitively abolishing Trim69-dependent polyubiquitination and degradation of FSP1.^133^ Upregulation of IGF2BP3 is strongly associated with early recurrence, tumor invasion, and poor prognosis in HCC. Silencing IGF2BP3 significantly promotes sorafenib-induced ferroptosis in HCC cells. Moreover, Nrf2 mRNA was identified as an important target of IGF2BP3, which stabilizes Nrf2 mRNA via m^6^A modification. Upregulation of IGF2BP3 inhibits sorafenib-induced ferroptosis by promoting Nrf2 mRNA stability in an m^6^A-dependent manner,^221^ suggesting a new anticancer strategy aimed at improving the efficacy of sorafenib by inhibiting IGF2BP3. METTL3-mediated m^6^A modification of SLC7A11 promotes ferroptosis resistance in hepatoblastoma cells. METTL3/IGF2BP1-mediated m^6^A modification stabilizes SLC7A11 mRNA and upregulates SLC7A11 expression by inhibiting the deadenylation process in hepatoblastoma cells.^222^

Thyroid cancer: ALKBH5 inhibits the progression of thyroid cancer by enhancing ferroptosis through Nrf2 inactivation mediated by decreasing the m^6^A level in TIAM1.^223^ FTO inhibits the progression of thyroid cancer by inducing ferroptosis through m^6^A-mediated downregulation of SLC7A11.^224^

Breast cancer: METTL16 promotes the progression of breast cancer by inhibiting ferroptosis by epigenetically increasing GPX4 expression via m^6^A modification.^225^ The m^6^A modification level is reduced due to downregulation of METTL14 in anti-HER2 therapy-resistant breast cancer. This decrease in the m6A level prevents YTHDC2-mediated degradation of FGFR4 mRNA, thus leading to the accumulation of FGFR4 in resistant breast cancer cells. FGFR4 phosphorylates GSK-3β and activates β-catenin/TCF4 signaling to increase the transcription of the SLC7A11 and FPN1 genes. Upregulation of SLC7A11 and FPN1 accelerates GSH synthesis and Fe^2+^ efflux, which confer anti-HER2 resistance by attenuating ferroptosis in breast cancer cells. Roblitinib, a highly selective inhibitor of FGFR4, combats anti-HER2 resistance by inducing ferroptosis in refractory HER2-positive breast cancer.^226^

Other tumors: In GBM, NKAP inhibits ferroptosis by recruiting SFPQ to promote SLC7A11 mRNA splicing in a manner dependent on METTL3-mediated m^6^A modification.^227^ Upregulation of FTO promotes radioresistance by repressing ferroptosis through promotion of OTUB1 expression in nasopharyngeal carcinoma (NPC).^228^ ALKBH5 inhibits ferroptosis by activating Nrf2 in an m^6^A-IGF2BP2-dependent manner in hypopharyngeal squamous cell carcinoma (HPSCC).^229^ HIF-1α mediates lncRNA-CBSLR expression to recruit the YTHDF2 protein and CBS mRNA to form the CBSLR/YTHDF2/CBS complex, resulting in m6A-dependent destabilization of CBS mRNA and leading to decreased methylation of the ACSL4 protein and an increase in its ubiquitin–proteasome-dependent degradation, thereby inhibiting ferroptosis in GC cells.^230^

#### Noncoding RNA-induced modulation of ferroptosis in cancer

ncRNAs, encompassing microRNAs (miRNAs), lncRNAs, and circRNAs, do not encode proteins. However, ncRNAs are considered master regulators of various cellular processes, particularly in cancers, where they are implicated in all hallmarks of cancer. Recent studies have demonstrated that ncRNAs, particularly miRNAs, lncRNAs, and circRNAs, are involved in the biological process of ferroptosis by regulating the molecular mechanism of ferroptosis in tumor cells. We refer readers to some recent excellent reviews for a detailed discussion of the roles of ncRNAs in regulating ferroptosis in cancer.^231–234^

### Epigenetic and posttranslational modifications regulating ferroptosis in central nervous system (CNS) diseases

**Fig. 7:**
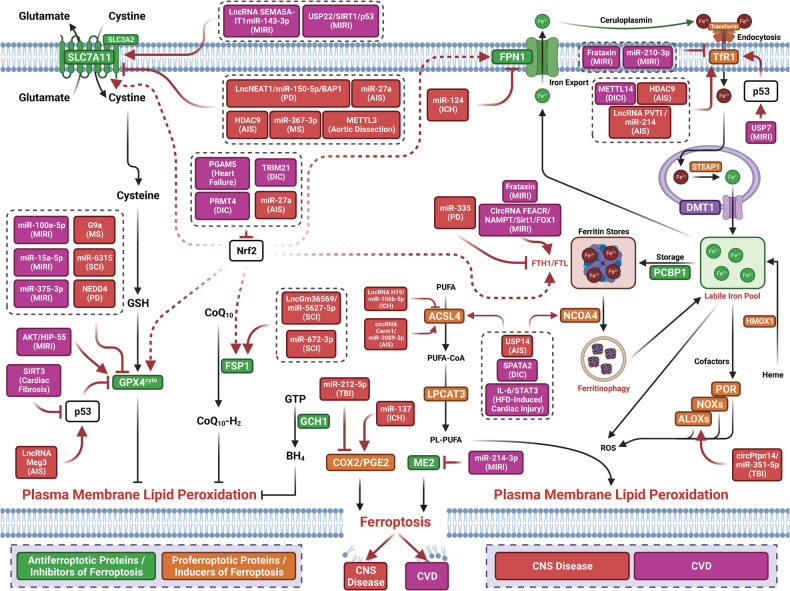
Epigenetic and posttranslational modification of ferroptosis in CNS disease and CVD. AIS acute ischemic stroke, BAP1 BRCA1-associated protein 1, BMSC-Exos bone marrow mesenchymal stem cells (BMSCs)-derived exosomes, CVD cardiovascular diseases, DIC doxorubicin-induced cardiomyopathy, HFD High-fat diet, Egr-1 transcription factor early growth response-1, FSP1 ferroptosis suppressor protein 1, FTH1 ferritin heavy chain 1, METTL14 methyltransferase-like 14, ME2 malic enzyme 2, MIRI myocardial ischemia/reperfusion injury, MPP^+^ 1-methyl-4-phenylpyridinium, MS multiple sclerosis, MSCs-exo mesenchymal stem cells-derived exosomes, NAMPT nicotinamide phosphoribosyltransferase, NHLRC1 NHL repeat-containing 1, Ptgs2 prostaglandin-endoperoxide synthase-2, TBI traumatic brain injury, SIC sepsis-induced cardiomyopathy, SPATA2 Spermatogenesis-associated protein 2, TfR1 transferrin receptor 1, TMEM43 transmembrane protein 43

Accumulating evidence supports neuronal ferroptosis as a critical factor in traumatic brain injury (TBI), multiple sclerosis (MS), spinal cord injury (SCI), neurodegenerative diseases such as Alzheimer’s disease (AD) and Parkinson’s disease (PD), and stroke, including spontaneous intracerebral hemorrhage (ICH), acute ischemic stroke (AIS), and subarachnoid hemorrhage (SAH); in addition, pharmacological inhibition of ferroptosis is supported as a therapeutic strategy for these diseases.^19^ Emerging evidence has revealed the role of epigenetic modifications and PTMs in regulating ferroptosis in CNS diseases. Below, we summarize the epigenetic modifications and PTMs regulating ferroptosis in CNS diseases (Table 8 and Fig. 7).

**Table 8:** Epigenetic and posttranslational modification of Ferroptosis in neurological diseases

Diseases	Modification	Targets	Biological functions	Ref
PD	ncRNA	SLC7A11	Upregulated LncNEAT1 promotes MPP^+^-induced ferroptosis via regulating LncNEAT1/miR-150-5p/BAP1/SLC7A11 pathway in SK-N-SH cells.	^41^
PD	ncRNA	FTH1	miR-335 promotes ferroptosis through the degrading of FTH1 in in vivo and in vitro 6-OHDA-stimulated models of PD.	^263^
PD	ncRNA	GPX4	Midbrain dopamine oxidation promotes ferroptosis of dopaminergic neurons through facilating NEDD4-mediated ubiquitination of GPX4.	^629^
AIS	ncRNA	ACSL4	Upregulated circular RNA Carm1 inhibit ferroptosis through binding circular RNA Carm1microRNA-3098-3p to downregulate ACSL4 in OGD/R-treated HT22 cells.	^293^
AIS	ncRNA	TFR1	Upregulated LncRNA PVT1 promotes ferroptosis through downregulating miR-214-mediated TFR1 and p53.	^294^
AIS	ncRNA	Nrf2	miR-27a promotes ferroptosis through inhibiting Nrf2 during ischemic stroke.	^295^
AIS	ncRNA	SLC7A11	miR-27a promotes ferroptosis through to aggravate cerebral ischemia-reperfusion injury through inhibiting SLC7A11.	^296^
AIS	ncRNA	GPX4	miR-760-3p in ADSC-Exo contributed to their function in inhibiting ferroptosis by targeting CHAC1 in neurons.	^297^
AIS	ncRNA	ATG7	GATA6 suppresses neuronal autophagy and ferroptosis through miR-193b/ATG7 axis-dependent Mechanism.	^298^
AIS	ncRNA	GPX4	LncRNA Meg3 promotes OGD hyperglycemic reperfusion-induced ferroptosis through upregulating p53, thereby inhibiting GPX4 in RBMVECs.	^299^
AIS	Methylation	PINK1	ELAVL1 suppresses ferroptosis-induced cerebral I/R and subsequent brain damage through inhibiting PINK1 expression via stabilizing DNMT3B mRNA.	^300^
AIS	Ubiquitination	NCOA4	USP14 stabilizes NCOA4 to promotes ferritinophagy-mediated ferroptosis in ischemic stroke.	^301^
AIS	Acetylation	TfR1/GPX4	HDAC9 promotes neuronal ferroptosis through increasing HIF-1 by deacetylation and deubiquitination, thus promoting the transcription of TfR1 and reducing Sp1 protein levels by deacetylation and ubiquitination, thus resulting in a down-regulation of GPX4 in in vitro and in vivo models of stroke.	^302^
ICH	ncRNA	ACSL4	LncRNA H19 protects against intracerebral hemorrhage injuries via regulating microRNA-106b-5p/ACSL4.	^329^
ICH	ncRNA	COX2/PGE2	miR-137 inhibit ferroptosis in oxyHb-treated SH-SY5Y cells via COX2/PGE2 pathway.	^330^
ICH	ncRNA	FPN	Downregulation of miR-124 enhances FPN expression and attenuates iron accumulation.	^331^
ICH	ncRNA	Nrf2/GPX4	Acupuncture may alleviate the neuronal cell death, inflammation, and ferroptosis after ICH by down-regulating miR-23a-3p.	^332^
TBI	ncRNA	5-LOX	Increased circPtpn14 (mmu_circ_0000130), which sponge miR-351-5p, thereby upregulate the expression of 5-ALOX.	^589^
TBI	ncRNA	Ptgs2	Decreased miR-212-5p was found in the TBI. Overexpression of miR-212-5p attenuates cell death through inhibiting ferroptosis via repressing Ptgs2.	^354^
SCI	ncRNA	FSP1	miR-672-3p enhances functional recovery with contusive SCI through inhibiting ferroptosis via upregulating FSP1.	^363^
SCI	ncRNA	FSP1	MSCs-exosomes lncGm36569 attenuates neuronal dysfunction through inhibiting ferroptosis via sponging miR-5627-5p to induce FSP1 upregulation.	^364^
SCI	ncRNA	GPX4	Silencing miR-6315 attenuates neuronal dysfunction through inhibiting ferroptosis via upregulating GPX4.	^365^
SCI	Ubiquitination	HMOX-1	Downregulated USP7 and upregulated HMOX-1 was found in SCI rat models. USP7 overexpression alleviates SCI through facilitating the expression of HMOX-1 through deubiquitination, thereby reducing ferroptosis.	^366^
MS	Methylation	GPX4	The histone methyltransferase G9a promotes neurodegeneration through inducing ferroptosis via catalyzing the repressive mark H3K9me2 that suppresses the expression of GCLC, CBS, and GPX4.	^378^
MS	ncRNA	EZH2/SLC7A11	BMSC-Exos containing miR-367-3p attenuates the severity of EAE through suppressing ferroptosis via restraining EZH2 expression, leading to the overexpression of SLC7A11.	^372^

*AIS* acute ischemic stroke, *BAP1* BRCA1-associated protein 1, *BMSC-Exos* bone marrow mesenchymal stem cells (BMSCs)-derived exosomes, *EAE* experimental autoimmune encephalomyelitis a typical animal model of MS, *EZH2* Enhancer of zeste homolog 2, *FSP1* ferroptosis suppressor protein 1, *FTH1* erritin heavy chain 1, *MPP*^*+*^ 1-methyl-4-phenylpyridinium, *MS* multiple sclerosis, *MSCs-exo* mesenchymal stem cells-derived exosomes, *Ptgs2* prostaglandin-endoperoxide synthase-2, *TBI* traumatic brain injury

#### Parkinson’s disease

PD is the second most common neurodegenerative disease and is a progressive condition pathologically characterized by the degeneration of nigrostriatal dopaminergic neurons located in the substantia nigra pars compacta (SNpc) of the brainstem and the presence of Lewy bodies, which consist mainly of misfolded α-synuclein, Parkin, ubiquitin, PTEN-induced kinase-1 (PINK1), and other proteins, in the surviving neurons.^235–238^ PD is characterized by motor and nonmotor symptoms and affects more than 2% of the population above 65 years of age.^237,239^ Accumulating evidence indicates that ferroptosis plays a role in the genesis of PD.^19,87,240–262^ However, data revealing the roles of epigenetic regulation of ferroptosis by ncRNAs in PD are limited. Upregulation of the lncRNA NEAT1 was observed in 1-methyl-4-phenylpyridinium (MPP^+^)-treated SK-N-SH cells. Silencing lncRNA NEAT1 was found to increase cell viability through ferroptosis inhibition by sponging and suppressing miR-150-5p.^41^ Overexpression of miR-150-5p upregulates SLC7A11 expression by directly binding to BAP1 to suppress ferroptosis. BAP1 overexpression or miR-150-5p inhibition mitigate sh-NEAT1-mediated inhibition of ferroptosis.^41^ Together, these observations indicate that upregulation of NEAT1 promotes MPP^+^-induced ferroptosis by regulating the miR-150-5p/BAP1/SLC7A11 pathway in SK-N-SH cells.^41^ miR-335 was found to promote ferroptosis through the degradation of FTH1 in vivo and in vitro in 6-hydroxydopamine (6-OHDA)-induced models of PD.^263^ Midbrain dopamine oxidation promotes ferroptosis in dopaminergic neurons by facilitating NEDD4-mediated ubiquitination of GPX4.^240^

#### Acute ischemic stroke

Caused by arterial occlusion, AIS is the most common type of stroke, represents a significant threat to human life and is among the most frequent causes of disability and death worldwide.^264,265^ Emerging evidence has shown that ferroptosis plays a role in the genesis of neuronal injury after ischemic stroke.^19,266–292^ Recently, accumulating evidence has revealed the roles of epigenetic regulation of ferroptosis via ncRNA expression, methylation, ubiquitination, and acetylation in AIS.^293–302^ Upregulation of the circRNA Carm1 inhibits ferroptosis through its binding to microRNA-3098-3p to downregulate ACSL4 in OGD/R-treated HT22 cells.^293^ Upregulation of LncRNA PVT1 promotes ferroptosis through miR-214-mediated suppression of TFR1 and P53 expression.^294^ miR-27a promotes ferroptosis by inhibiting Nrf2 expression during ischemic stroke.^295^ miR-27a aggravates cerebral ischemia/reperfusion injury (IRI) by promoting ferroptosis through inhibition of SLC7A11.^296^ miR-760-3p in exosomes from adipose-derived stem cells (ADSC-Exos) inhibits ferroptosis by targeting CHAC1 in neurons.^297^ GATA6 suppresses neuronal autophagy and ferroptosis through a miR-193b/ATG7 axis-dependent mechanism.^298^ LncRNA Meg3 promotes oxygen-glucose deprivation (OGD)-mediated hyperglycemic reperfusion-induced ferroptosis by upregulating p53, thereby inhibiting GPX4 expression in rat brain microvascular endothelial cells (RBMVECs).^299^ ELAVL1 suppresses ferroptosis-induced cerebral IRI and subsequent brain damage by inhibiting PINK1 expression through stabilization of DNMT3B mRNA.^300^ USP14 stabilizes NCOA4 to promote ferritinophagy-mediated ferroptosis in ischemic stroke.^301^ HDAC9 promotes neuronal ferroptosis through deacetylation and deubiquitination, dependently increasing HIF-1 expression and thus increasing the transcription of TfR1 and decreasing the Sp1 protein level by deacetylation and ubiquitination, leading to downregulation of GPX4 in in vitro and in vivo models of stroke.^302^

#### Intracerebral hemorrhage

ICHs are defined by brain injury resulting from acute extravasation of blood into the brain parenchyma from a ruptured cerebral blood vessel.^303^ Occurring spontaneously and accounting for 80% of hemorrhagic strokes and 10-15% of all strokes,^303^ ICH is an acute subtype of cerebral stroke. Emerging evidence has shown that ferroptosis plays a role in the genesis of secondary brain injury after ICH (SBI-ICH).^19,304–328^ Data revealing the roles of epigenetic regulation of ferroptosis by ncRNAs in ICH are limited. LncRNA H19 protects against ICH-related injuries by regulating the microRNA-106b-5p/ACSL4 axis.^329^ miR-137 inhibits ferroptosis in oxyhemoglobin (oxyHb)-treated SH-SY5Y cells via the COX2/PGE2 pathway.^330^ Downregulation of miR-124 induces FPN expression and attenuates iron accumulation.^331^ Acupuncture may alleviate neuronal cell death, inflammation, and ferroptosis after ICH by downregulating miR-23a-3p.^332^

#### Traumatic brain injury

Structural and physiological disruption of brain function caused by external forces results in TBI, which is a major cause of disability and death in patients worldwide and a key injury mechanism involving the death of nerve cells.^333–337^ Emerging evidence indicates that ferroptosis plays a vital role in secondary brain injury and worsens long-term outcomes after TBI.^338–353^ Emerging studies have revealed the roles of epigenetic regulation of ferroptosis by ncRNAs in TBI.^354^ Upregulated circPtpn14 (mmu_circ_0000130) promotes ferroptosis by sponging miR-351-5p, which targets the 5-LOX mRNA for degradation, thereby upregulating the expression of 5-LOX after TBI. Decreased miR-212-5p expression has been found in TBI. Overexpression of miR-212-5p attenuates cell death by inhibiting ferroptosis through repression of Ptgs2 after TBI.^354^

#### Spinal cord injury

SCI is a severely disabling neurological condition causing primary damage to the spinal cord via compression and laceration, followed by secondary damage consisting of inflammation and ischemia, culminating in substantial loss of tissue.^355–359^ Ferroptosis plays a key role in secondary SCI, and this role is closely related to inflammation, immunity, and chronic injuries.^339,340,360–362^ Emerging studies have revealed the roles of epigenetic regulation of ferroptosis by ncRNAs and ubiquitination in SCI.^354^ miR-672-3p enhances functional recovery by inhibiting ferroptosis via upregulation of FSP1 in contusive SCI.^363^Mesenchymal stem cell (MSC) exosomal lncGm36569 attenuates neuronal dysfunction through ferroptosis inhibition by sponging miR-5627-5p to induce FSP1 upregulation.^364^ Silencing miR-6315 attenuates neuronal dysfunction by inhibiting ferroptosis via upregulation of GPX4.^365^ Downregulated USP7 expression and upregulated HMOX-1 expression were found in SCI rat models. USP7 overexpression alleviates SCI through deubiquitination of HMOX-1 and promotion of its expression, thereby reducing ferroptosis.^366^

#### Multiple sclerosis

A CNS disorder characterized by inflammation, demyelination, gliosis and neuroaxonal degeneration, MS is highly heterogeneous and affects over 2.8 million people worldwide.^367–371^ Recently, ferroptosis was shown to play a key role in the genesis of MS, and inhibition of ferroptosis was found to attenuate disease progression in an experimental autoimmune encephalitis (EAE) mouse model.^372–377^ Emerging studies have revealed the roles of epigenetic regulation of ferroptosis by ncRNAs and methylation in MS. The histone methyltransferase G9a promotes neurodegeneration by inducing ferroptosis by catalyzing the writing of the repressive mark H3K9me2, thereby suppressing the expression of GCLC, CBS, and GPX4.^378^ In addition, bone marrow mesenchymal stem cell-derived exosomes (BMSC-Exos) containing miR-367-3p were found to attenuate the severity of EAE by inhibiting ferroptosis through repression of EZH2 expression, leading to overexpression of SLC7A11.^372^

### Epigenetic and posttranslational modifications regulating ferroptosis in cardiovascular diseases

Accumulating evidence supports ferroptosis as a critical factor in CVDs, including myocardial IRI (MIRI), doxorubicin (DOX)-induced cardiomyopathy (DIC), cardiac fibrosis (CF), heart failure, high-fat diet (HFD)-induced cardiac injury, cardiac hypertrophy, aortic dissection (AD), and septic cardiomyopathy (SCM). Below, we summarize the epigenetic regulation of ferroptosis in CVDs (Table 9 and Fig. 7).

**Table 9:** Epigenetic and posttranslational modification of ferroptosis in cardiovascular diseases

Diseases	Modification	Targets	Biological functions	Ref
MIRI	ncRNA	NAMPT/Sirt1/FOX1/FTH1	CircRNA FEACR alleviates MIRI through inhibiting ferroptosis by interacting with NAMPT, which increased NAMPT-dependent Sirtuin1 (Sirt1) expression, thereby promoting the transcriptional activity of FOXO1 by reducing FOXO1 acetylation levels, eventually upregulating the transcription of FTH1.	^396^
MIRI	ncRNA	SLC7A11	Upregulated LncRNA SEMA5A-IT1 inhibits cardiomyocytes against hypoxia/reoxygenation injury partly through inhibiting ferroptosis via upregulating SLC7A11 by sponging miR-143-3p.	^397^
MIRI	ncRNA	TfR1	miR-210-3p alleviate hypoxia/reoxygenation-induced myocardial cell injury through inhibiting ferroptosis via dowregulating TfR1.	^398^
MIRI	ncRNA	GLS2	miR-190a-5p promotes ferroptosis through inhibiting GLS2.	^399^
MIRI	ncRNA	GPX4	MiR-199a-5p promotes cardiomyocyte death in OGD/R-treated H9c2 cells through inducing ferroptosis via downregulation of GPX4 by inhibiting Akt/eNOS signaling pathway.	^400^
MIRI	ncRNA	ME2	Upregulated miR-214-3p promotes MIRI through inducing ferroptosis via suppressing ME2.	^401^
MIRI	ncRNA	GPX4	Egr-1-mediated upregulation of miR-15a-5p promotes MIRI through inducing ferroptosis via suppressing GPX4.	^402^
MIRI	Ubiquitination	Frataxin	Upregulated frataxin alleviates cardiomyocyte ferroptosis through upregulating FTH and downregulating TfR1. NHLRC1 mediates frataxin ubiquitination degradation.	^403^
MIRI	Ubiquitination	p53/TfR1	USP7 promotes MIRI through inducing ferroptosis via activation of the p53/TfR1 pathway.	^385^
MIRI	Ubiquitination	SIRT1/p53/SLC7A11	USP22 alleviates MIRI through inhibiting ferroptosis via the SIRT1-p53/SLC7A11.	^404^
Myocardial infarction	Phosphorylation	AKT/HIP-55	Upregulated HIP-55 alleviates cardiomyocyte ferroptosis and MI injury.HIP-55 was identified as a new AKT substrate. AKT phosphorylates HIP-55 at S269/T291 sites and further HIP-55 directs AKT signaling to negatively regulate the MAP4K1 pathway against MI injury in a site-specific manner. S269A/T291A-mutated HIP-55 (HIP-55AA), which is defective in AKT phosphorylation and significantly decreases the interaction between HIP-55 and MAP4K1, failed to inhibit the MAP4K1/GPX4 ferroptosis.	^405^
DIC	m^6^A	TFRC	METTL14 promotes ferroptosis in doxorubicin-induced cardiomyocyte through stabilizing KCNQ1OT1 in a IGF2BP1 m^6^A manner to sponge miR-7-5p, thereby increasing levels of transferrin receptor.	^410^
DIC	Methylation	Nrf2/GPX4	Upregulated PRMT4 aggravate DIC through promoting ferroptosis via interacting with Nrf2 to promote its enzymatic methylation, thereby suppressing GPX4.	^411^
DIC	Phosphorylation	AMPKα2	Activation of AMPKα2 attenuated doxorubicin-induced cardiotoxicity through inhibiting ferroptosis.	^412^
DIC	Ubiquitination	Keap1	TRIM21 ablation protects DIC through inhibiting ferroptosis via enhancing p62 sequestration of Keap1.	^413^
DIC	Ubiquitination	-	MITOL knockdown worsened vulnerability to DOX in cultured cardiomyocytes stressed with DOX.	^414^
DIC	Ubiquitination	NCOA4	Upregulated SPATA2 recruit CYLD promotes ferritinophagy through decreasing NCOA4 ubiquitination and ferritin, and ferroptosis through increasing ACSL4.	^415^
Cardiac Fibrosis	Acetylation	p53	Knockout of SIRT3 results in increased p53 acetylation and ferroptosis through downregulating GPX4 in the mouse hearts. SIRT3-mediated cardiac fibrosis was partly through a mechanism involving p53 acetylation-induced ferroptosis in myofibroblasts.	^416^
Cardiac Fibrosis	ncRNA	GPX4	miR-375-3p promotes cardiac fibrosis through accelerating the ferroptosis of cardiomyocytes via downregulating GPX4.	^417^
Heart Failure	ncRNA	GPX4	circSnx12 could act as an endogenous sponge to bind with miR-224-5p, and the 3’UTR region of FTH1 also had miRNA binding sites.	^418^
Heart Failure	Ubiquitination	Keap1/Nrf2	Downregulated PGAM5 promotes ferroptosis in models of heart failure through decreasing Keap1 protein ubiquitination, thereby reducing activation of Nrf2.	^419^
HFD-induced cardiac injury	Phosphorylation	STAT3/NCOA4	IL-6/STAT3 signaling promotes cardiac injury by upregulating NCOA4-mediated ferritinophagy and ferroptosis in high-fat-diet fed mice.	^420^
Cardiac Hypertrophy	Glycosylation	CD147	CD147 promotes pathological cardiac remodeling and dysfunction through promoting ferroptosis in a glycosylation-dependent manner through binding the adaptor protein TRAF2 and activating the downstream TRAF2-TAK1 signaling pathway.	^421^
Aortic dissection	m^6^A	SLC7A11	Upregulated METTL3 facilitates ferroptosis of HASMCs by promoting the mRNA degradation of SLC7A11 and FSP1.	^422^
SIC	-	SLC7A11/GPX4; P53 and ferritin	TMEM43 knockdown promotes LPS-induced mouse cardiac injury and dysfunction through inducing ferroptosis via upregulating the level of P53 and ferritin, while inhibiting the level of GPX4 and SLC7A11.	^425^

*CYLD* cylindromatosis a deubiquitinating enzyme, *Egr-1* transcription factor early growth response-1, *FTH1* erritin heavy chain 1, *DIC* doxorubicin-induced cardiomyopathy, *FOXO1* forkhead box protein O1, *FTH1* ferritin heavy chain 1, *HFD* high-fat diet, *METTL14* methyltransferase-like 14, ME2 malic enzyme 2, *MIRI* myocardial ischemia/reperfusion injury, *NAMPT* nicotinamide phosphoribosyltransferase, *NHLRC1* NHL repeat-containing 1, *OGD/R* oxygen-glucose deprivation/reperfusion, *SIC* sepsis-induced cardiomyopathy, *SPATA2* Spermatogenesis-associated protein 2, *TfR1* transferrin receptor 1, *TMEM43* transmembrane protein 43

#### Myocardial ischemia/reperfusion injury

Myocardial reperfusion strategies and reoxygenation are the preferred and effective treatments for acute myocardial infarction (AMI).^379,380^ However, reperfusion inevitably causes cardiomyocyte death, increases the infarct size, and aggravates the condition; these events are collectively referred to as MIRI.^381^ MIRI leads to energy metabolism disruption and oxidative stress, among other issues.^382^ A recent novel study revealed a role of ferroptosis in MIRI in murine models, establishing a correlation between cardiac cell death and ferroptosis in vivo.^383,384^ Thereafter, emerging studies have attempted to elucidate the pathophysiological role of ferroptosis in the genesis of MIRI.^92,382,383,385–395^ There are also emerging studies that have revealed the roles of epigenetic regulation of ferroptosis by ncRNAs, ubiquitination, and phosphorylation in MIRI^385,396–405^ (Table 9). CircRNA FEACR alleviates MIRI through inhibition of ferroptosis by interacting with nicotinamide phosphoribosyltransferase (NAMPT), which upregulates NAMPT-dependent sirtuin1 (Sirt1) expression, thereby reducing the forkhead box protein O1 (FOXO1) acetylation level to promote its transcriptional activity, eventually upregulating the transcription of FTH1.^396^ Upregulation of LncRNA SEMA5A-IT1 protects cardiomyocytes against hypoxia/reoxygenation (H/R) injury partially through inhibition of ferroptosis by sponging miR-143-3p to upregulate SLC7A11.^397^ miR-210-3p attenuates myocardial cell injury induced by H/R through inhibition of ferroptosis by downregulating TfR1.^398^ miR-190a-5p promotes ferroptosis through inhibition of GLS2 expression.^399^ MiR-199a-5p promotes cardiomyocyte death in OGD/reperfusion (OGD/R)-treated H9c2 cells by inducing ferroptosis via downregulation of GPX4 through inhibition of the Akt/eNOS signaling pathway.^400^ Upregulated expression of miR-214-3p promotes MIRI by inducing ferroptosis through suppression of ME2.^401^ Egr-1-mediated upregulation of miR-15a-5p promotes MIRI by inducing ferroptosis through suppression of GPX4.^402^ Upregulated expression of frataxin alleviates cardiomyocyte ferroptosis. NHL repeat-containing 1 (NHLRC1) is an E3 ligase that mediates ubiquitin-mediated degradation of frataxin.^403^ USP7 promotes MIRI by inducing ferroptosis via activation of the p53/TfR1 pathway.^385^ USP22 alleviates MIRI by inhibiting ferroptosis via the SIRT1-p53/SLC7A11 axis.^404^ The expression of SIRT1, USP22, and SLC7A11 is inhibited by IRI, whereas increased expression of p53 is found in affected myocardial tissues. Conversely, overexpression of USP22, SIRT1, or SLC7A11 inhibits IRI and increases the viability of cardiomyocytes by inhibiting ferroptosis.^404^ Upregulated expression of HIP-55 alleviates cardiomyocyte ferroptosis and MI injury.^405^ HIP-55 is phosphorylated by AKT at S269/T291, leading to negatively regulation of MAP4K1 by AKT to attenuate MI injury in a site-specific manner, as evidenced by the finding that S269A/T291A-mutated HIP-55 (HIP-55AA) decreased the interaction between HIP-55 and MAP4K1, resulting in loss of ferroptosis inhibition.^405^

#### Doxorubicin-induced cardiomyopathy

Anthracycline-based chemotherapy can lead to the progressive development of cardiomyopathy. Although DOX is an effective chemotherapeutic agent prescribed to treat breast, ovarian, and gastrointestinal cancers, it causes cardiotoxicity, resulting in DCM, which leads to congestive heart failure.^25,406^ The exact mechanisms of DOX-induced cardiotoxicity remain poorly understood. However, emerging studies have attempted to elucidate the pathophysiological role of ferroptosis in the genesis of DCM.^25,383,407–409^ In addition, emerging studies have revealed the roles of epigenetic regulation of ferroptosis by m^6^A modification, other methylation modifications, phosphorylation, and ubiquitination in DCM^410–415^ (Table 9). METTL14 promotes ferroptosis in DOX-induced cardiomyocytes by mediating IGF2BP1 m6A-dependent stabilization of KCNQ1OT1 to sponge miR-7-5p, thereby increasing the level of the transferrin receptor.^410^ Upregulated expression of PRMT4 aggravates DIC by promoting ferroptosis via an interaction with Nrf2 to promote its enzymatic methylation, thereby suppressing GPX4 expression.^411^ Activation of AMPK α2 attenuates DOX-induced cardiotoxicity by inhibiting ferroptosis.^412^ TRIM21 ablation protects against DIC through inhibition of ferroptosis by enhancing sequestration of Keap1 by P62.^413^ MITOL knockdown reduced susceptibility to DOX in cultured cardiomyocytes stressed with DOX.^414^ Overexpressed SPATA2 recruits CYLD to promote ferritinophagy by decreasing NCOA4 ubiquitination and ferritin expression and promote ferroptosis by increasing ACSL4 expression.^415^

#### Cardiac fibrosis

Knockout of SIRT3 results in increased p53 acetylation and ferroptosis through downregulation of GPX4 in mouse hearts. p53 acetylation-induced ferroptosis is partially involved in SIRT3-mediated CF in myofibroblasts.^416^ MiR-375-3p promotes CF by accelerating ferroptosis in cardiomyocytes by downregulating GPX4.^417^

#### Heart failure

circSnx12 functions as an endogenous sponge to bind miR-224-5p, and FTH1 has miRNA binding sites in its 3’UTR.^418^ Downregulated expression of PGAM5 promotes ferroptosis in models of heart failure by decreasing Keap1 ubiquitination, thereby reducing the activation of Nrf2.^419^

#### HFD-induced cardiac injury

The IL-6/STAT3 axis exacerbates cardiac injury by increasing NCOA4-mediated ferritinophagy and ferroptosis in HFD-fed mice.^420^

#### Cardiac hypertrophy

CD147 promotes cardiac remodeling and dysfunction by promoting ferroptosis in a glycosylation-dependent manner by binding to TRAF2 to activate the downstream TRAF2-TAK1 axis.^421^

#### Aortic dissection

Upregulated expression of METTL3 facilitates ferroptosis in human aortic smooth muscle cells by promoting the degradation of SLC7A11 and FSP1 mRNA.^422^

#### Septic cardiomyopathy

Seventy percent of patients with sepsis have SCM, which is the leading cause of sepsis-related mortality and morbidity.^423,424^ Ferroptosis was recently found to play a key role in the genesis of SCM, and inhibition of ferroptosis attenuates SCM progression.^425–429^ Thus, ferroptosis is involved in SCM. Emerging studies have revealed the roles of epigenetic regulation of ferroptosis in SCM^410–415^ (Table 9). TMEM43 knockdown promotes lipopolysaccharide (LPS)-induced cardiac injury and dysfunction in mice by inducing ferroptosis by increasing the levels of P53 and ferritin and decreasing the levels of GPX4 and SLC7A11.^425^

### Epigenetic and posttranslational modifications regulating ferroptosis in liver diseases

**Fig. 8:**
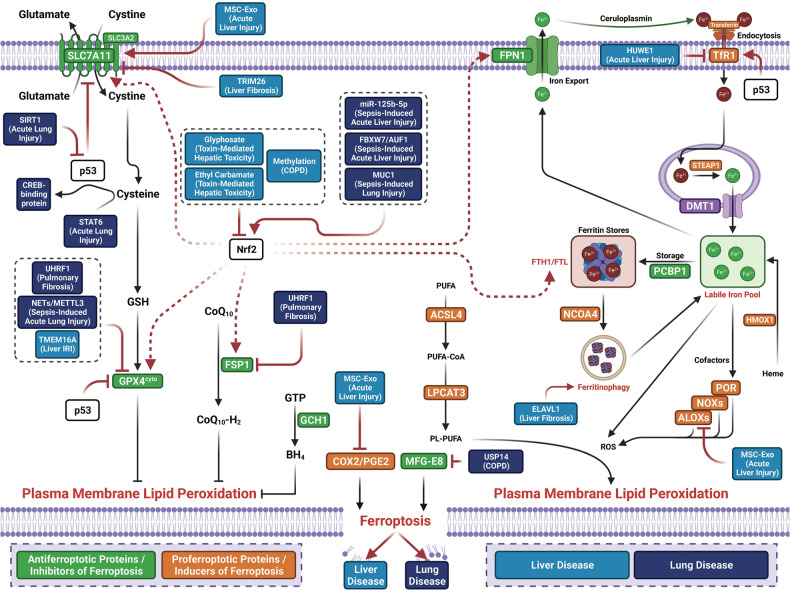
Epigenetic and posttranslational modification of ferroptosis in Liver diseases and Lung diseases. AUF1 AU-rich element (ARE)-binding factor 1, BRD7 bromodomain-containing protein 7, COPD chronic obstructive pulmonary disease, GCN5L1 mitochondrial general control of amino acid synthesis 5 like 1, IRI ischemia/reperfusion injury, LOXs lipoxygenases, NETs neutrophil extracellular traps, Ptgs2 prostaglandin-endoperoxide synthase 2, STAT3 signal transducer and activator of transcription 3, TfR1 transferrin receptor 1, TMEM16A transmembrane member 16A, TMEM16A transmembrane member 16A

Accumulating studies have shown that ferroptosis plays a role in the genesis of liver diseases,^29,30^ and pharmacological induction and inhibition of ferroptosis show significant potential utility for the treatment of hepatic disorders.^430–434^ Accumulating evidence indicates epigenetic modifications regulate ferroptosis in liver diseases, including acute liver injury (ALI), nonalcoholic fatty liver disease (NAFLD), liver fibrosis, hepatic ischemia/reperfusion injury, and toxin-mediated hepatic toxicity. In this section, we summarize the epigenetic regulation of ferroptosis by ubiquitination, phosphorylation, and acetylation in liver diseases (Table 10 and Fig. 8).

**Table 10:** Epigenetic and posttranslational modification of ferroptosis in liver diseases

Disease	Modification	Targets	Biological functions	Ref
Acute liver injury	Ubiquitination	SLC7A11	MSCs and MSC-derived exosomes (MSC-Exo) achieved pathological remission through inhibiting ferroptosis Via downregulating the mRNA level of Ptgs2 and LOXs while increasing protein level of SLC7A11 in CCl_4_-induced ALI. MSC-Exo-induced expression of SLC7A11 protein was accompanied by increasing of CD44 and OTUB1. The aberrant expression of ubiquitinated SLC7A11 triggered by CCl_4_ could be rescued with OTUB1-mediated deubiquitination, thus strengthening SLC7A11 stability and thereby leading to the activation of system X_C_^-^ to prevent CCl4-induced hepatocyte ferroptosis.	^445^
Acute liver injury	Ubiquitination	TFRC	Upregulated ubiquitin E3 ligase HUWE1 inhibit ferroptosis through targeting TfR1 for ubiquitination and proteasomal degradation degradating in CCl_4_-induced liver injury.	^446^
Liver fibrosis	Ubiquitination	ELAVL1	Upregulated ELAVL1 induces liver fibrosis through promoting ferritinophagy in HSCs.	^459^
Liver fibrosis	Phosphorylation	ZFP36	Downregulated ZFP36 by ubiquitin ligase FBXW7/CDC4 promotes ferritinophagy activation, and ferroptosis induction in human HSCs.	^458^
Liver fibrosis	Ubiquitination	p53	XC inhibition-, GPX4 inhibition-, and GSH depletion-mediated BRD7 upregulation triggers p53 mitochondrial translocation via direct binding with N-terminal transactivation domain, thus aggravating the accumulation of mitochondrial iron, the hyperfunction of electron transfer chain, lipid peroxidation, and eventually leading to iron-dependent ferroptosis.	^460^
Liver fibrosis	Ubiquitination	SLC7A11	TRIM26 promotes HSCs ferroptosis to suppress liver fibrosis through mediating the ubiquitination of SLC7A11.	^461^
NAFLD	Acetylation	CypD	GCN5L1 expression was increased in NASH patients and in NASH mice. GCN5L1 acetylated CypD and enhanced its binding with ATP5B, resulting in mitochondrial ROS into the cytoplasm, which promotes ferroptosis of hepatocytes and induces accumulation of high mobility group box 1 in the microenvironment, thereby inducing the generation of NETs.	^462^
Liver IRI	Ubiquitination	GPX4	TMEM16A interacts with GPX4 to induce its ubiquitination and degradation, thereby enhancing ferroptosis. TMEM16A deficiency alleviates hepatic IRI via suppressing GPX4-mediated ferroptosis.	^463^
Toxin -mediated Hepatic toxicity	Phosphorylation	Nrf2	Ethyl carbamate induces ferroptosis through inhibiting activation of Nrf2 through repressing its phosphorylation modification and nuclear translocation, thereby inhibiting SLC7A11, leading to GSH depletion.	^464^
Toxin -mediated Hepatic toxicity	Phosphorylation	Nrf2	Glyphosate triggers ferroptosis in hepatocyte through suppressing Nrf2 via blocking the phosphorylation and nuclear translocation of Nrf2, resulting in GSH depletion and inhibition of GPX4.	^465^

*BRD7* bromodomain-containing protein 7, *GCN5L1* mitochondrial general control of amino acid synthesis 5 like 1, *Ptgs2* prostaglandin-endoperoxide synthase 2, *LOXs* lipoxygenases, *TfR1* transferrin receptor 1, *NETs* neutrophil extracellular traps, *IRI* ischemia/reperfusion injury, *TMEM16A* transmembrane member 16A

#### Acute liver injury

ALI is the primary cause of liver diseases and is associated with high morbidity and mortality, causing 3.5% of deaths worldwide.^435^ Various hepatotoxic factors, including lipid deposition, viruses, and drugs, can induce ALI. Emerging studies have suggested that ferroptosis plays a role in the genesis of ALI^436–444^ and have revealed the roles of epigenetic regulation of ferroptosis by ubiquitination in ALI^410–415^ (Table 10). Mesenchymal stem cells (MSCs) and MSC-derived exosomes (MSC-Exos) alleviate injury through ferroptosis inhibition by decreasing the mRNA levels of Ptgs2 and LOXs while increasing the protein level of SLC7A11 in CCl_4_-induced ALI. MSC-Exo-induced expression of the SLC7A11 protein was found to be accompanied by increases in CD44 and OTUB1 expression. OTUB1-mediated deubiquitination rescued CCl_4_-triggered ubiquitination of SLC7A11, thus stabilizing SLC7A11 to activate system X_C_^−^ and thereby preventing hepatocyte ferroptosis.^445^ The overexpressed ubiquitin E3 ligase HUWE1 inhibits ferroptosis by targeting TfR1 for ubiquitination and proteasomal degradation in the context of CCl_4_-induced liver injury, suggesting that HUWE1 functions as an inhibitor to mitigate ALI by antagonizing both aberrant iron accumulation and ferroptosis.^446^

#### Liver fibrosis

Liver fibrosis is a pathological state and abnormal repair response to chronic liver injury that is characterized by diffuse and progressive excessive deposition of extracellular matrix (ECM) in the liver. Liver fibrosis typically results from chronic liver damage resulting from infection, toxin or drug exposure, NAFLD, cholestasis, or autoimmune insults.^447,448^ Liver fibrosis has become one of the leading causes of liver disease globally and often progresses to liver cirrhosis and HCC. Accumulating evidence has shown that ferroptosis is involved in the pathogenesis of liver fibrosis.^449–457^ Recent studies have also shown the roles of epigenetic regulation of ferroptosis by phosphorylation^458^ and ubiquitination^459–461^ in liver fibrosis. Upregulated expression of ELAVL1 induces liver fibrosis by promoting ferritinophagy in hepatic stellate cells (HSCs).^459^ Downregulation of ZFP36 mediated by the ubiquitin ligase FBXW7/CDC4 promotes activation of ferritinophagy and induction of ferroptosis in human HSCs.^458^ BRD7 upregulation induced by xCT inhibition, GPX4 inhibition, and GSH depletion triggers mitochondrial translocation of p53 via direct binding to the N-terminal transactivation domain, thus increasing mitochondrial iron and LPO and eventually causing ferroptosis.^460^ TRIM26 mediates the ubiquitination of SLC7A11 to promote ferroptosis in HSCs, thereby suppressing liver fibrosis.^461^

#### Nonalcoholic fatty liver disease

GCN5L1 expression has been found to be increased in human patients and mice with nonalcoholic steatohepatitis (NASH). GCN5L1 acetylates CypD to enhance its binding to ATP5B, resulting in the release of mitochondrial ROS (mtROS) into the cytoplasm, which promotes ferroptosis in hepatocytes and induces the accumulation of high mobility group box 1 in the microenvironment, thereby inducing the formation of neutrophil extracellular traps (NETs).^462^

#### Hepatic ischemia/reperfusion injury

TMEM16A ubiquitinates and degrades GPX4 to induce ferroptosis. TMEM16A deficiency alleviates hepatic IRI by suppressing GPX4-mediated ferroptosis.^463^

#### Toxin-mediated hepatic toxicity

Ethyl carbamate induces ferroptosis by inhibiting the activation of Nrf2 through suppression of its phosphorylation and nuclear translocation, thereby inhibiting SLC7A11 expression and leading to GSH depletion.^464^ Glyphosate triggers ferroptosis in hepatocytes by suppressing Nrf2 via blockade of Nrf2 phosphorylation and nuclear translocation, resulting in GSH depletion and inhibition of GPX4.^465^

### Epigenetic and posttranslational modifications regulating ferroptosis in lung diseases

Accumulating studies have shown epigenetic regulation of ferroptosis in the genesis of lung diseases, including chronic obstructive pulmonary disease (COPD), acute lung injury, pulmonary fibrosis, and sepsis-induced acute lung injury. In this section, we summarize the epigenetic regulation of ferroptosis in these lung diseases (Table 11 and Fig. 8).

**Table 11:** Epigenetic and posttranslational modification of ferroptosis in lung diseases

Disease	Modification	Targets	Biological functions	Ref
Acute lung injury	Acetylation	p53/SLC7A11	STAT6 alleviates acute lung injury through inhibiting ferroptosis via competitively binding with CREB-binding protein (a critical acetyltransferase of p53 acetylation), which inhibits p53 acetylation and transcriptionally restores SLC7A11 expression.	^471^
Acute lung injury	Acetylation	p53/SLC7A11/GPX4	Decreased SIRT1 trigger heat stress-induced lung epithelial cells injury through inducing ferroptosis via increasing acetylation of p53, which transcriptionally inhibits SLC7A11.	^472^
Acute lung injury	Phosphorylation	PERK	mtROS-initiated endoplasmic reticulum membrane (MAMs) dysfunction is partially implicated in arsenic-evoked ferroptosis and ALI.	^469^
Acute lung injury	Phosphorylation	STAT3	Nrf2 works together with and promotes phosphorylation of STAT3, through which collaborate to upregulate SLC7A11 to inhibit ferroptosis in intestinal ischemia/reperfusion-induced acute lung injury (IIR-ALI) model.	^470^
Sepsis-Induced Acute Lung Injury	m^6^A	GPX4	NETs induce ferroptosis through METTL3-induced m^6^A modification of GPX4 in the pathogenesis of sepsis-associated ALI.	^473^
Sepsis-Induced Acute Lung Injury	ncRNA	Nrf2/GPX4	miR-125b-5p in adipose derived stem cells exosomes alleviates the inflammation induced PMVECs ferroptosis in sepsis induced acute lung injury via regulating Keap1/Nrf2/GPX4 expression, hence improve the acute lung injury in sepsis.	^480^
Sepsis-Induced Acute Lung Injury	Phosphorylation	GSK3β/Nrf2/GPX4	Inhibition of MUC1 aggravates lung injury through triggering ferroptosis via increasing the expression level of Keap1, reducing the phosphorylation level of GSK3β, inhibiting the entry of Nrf2 into the nucleus, further inhibit the expression level of GPX4.	^474^
Sepsis-Induced Acute Lung Injury	Ubiquitination	AUF1/Nrf2/ATF3	FBXW7 mediates protein degradation of AUF1.AUF1 alleviate sepsis-induced acute lung injury through inhibiting ferroptosis by upregulating Nrf2 and down-regulating ATF3.	^475^
COPD	Methylation	RAP1A	NF-κB/RelA-mediated PRMT7 upregulaioned expression induces mono-methylation of histones at enhancers can regulate Rap1a expression, which is crucial for MAPK signaling downstream of G-protein coupled activation, integrin activation, and the subsequent adhesion and migration ability of monocytes Further, inflammatory macrophages via ALOX5-mediated release of LTB4 induced increased expression of ACSL4 in AT2 cells increasing susceptibility to cigarette smoke-induced ferroptosis and tissue injury.	^481^
COPD	Methylation	Nrf2/GPX4	Hypermethylation of the Nrf2 promoter-induced inhibition of Nrf2 induces ferroptosis through inhibiting GPX4 in cigarette smoke extract treated human bronchial epithelial (HBE) cells.	^482^
COPD	Ubiquitination	MFG-E8	Cigarette smoke-induced diminished USP14 expression leads to the proteasomal degradation of MFG-E8, which aggravates bronchial epithelial cell ferroptosis.	^483^
Pulmonary fibrosis	Methylation	GPX4 and FSP1	Upregulation de novo methylation regulator UHRF1 sensitively elevates CpG site methylation levels in promoters of both GPX4 and FSP1 genes and induces the epigenetic repression of both genes, subsequently leading to ferroptosis in chemically interfered AEC2 cells.	^630^
Pulmonary epithelial senescence	Acetylation	USP3/SIRT3/p53/SLC7A11	PM2.5 triggers pulmonary epithelial senescence and ferroptosis through decreasing USP3, by which leads to SIRT3 degradation via ubiquition proteasome pathway, thereby increasing p53 acetylation, which transcriptionally activates p21 and inhibits SLC7A11.	^598^

*AUF1* U-richelement(ARE)-binding factor1, *COPD* chronicobstructive pulmonary disease, *Ptgs2* prostaglandin-endoperoxide synthase 2, *LOXs* lipoxygenases, *TfR1* transferrin receptor 1, *NETs* neutrophil extracellular traps, *IRI* ischemia/reperfusion injury, *TMEM16A* transmembrane member 16A, *STAT3* signal transducer and activator of transcription 3

#### Acute lung injury

A common and critical illness caused by both pulmonary and extrapulmonary factors, acute lung injury (ALI) and its most severe form, acute respiratory distress syndrome (ARDS), result in high morbidity and mortality and have no effective treatments.^466,467^ Accumulating evidence has revealed that ferroptosis is involved in the pathogenesis of ALI.^42,468^ Recent studies have also shown the roles of epigenetic regulation of ferroptosis by phosphorylation^469,470^ and acetylation^471,472^ in acute lung injury. Signal transducer and activator of transcription 6 (STAT6) alleviates acute lung injury through ferroptosis inhibition by competitively binding the critical acetyltransferase for p53 acetylation, i.e., CREB-binding protein, to suppress p53 acetylation and transcriptionally restore the expression of SLC7A11.^471^ Decreased SIRT1 expression triggers heat stress-induced lung epithelial cell injury through ferroptosis induction by increasing the acetylation of P53, which transcriptionally inhibits SLC7A11.^472^ mtROS-initiated dysfunction of mitochondria-associated endoplasmic reticulum membranes (MAMs) is partially implicated in arsenic-induced ferroptosis and ALI.^469^ Nrf2 was found to activate and cooperate with STAT3 to upregulate SLC7A11 to inhibit ferroptosis in an intestinal ischemia/reperfusion-induced acute lung injury (IIR-ALI) model.^470^

#### Sepsis-associated acute lung injury (SALI)

Accumulating evidence has demonstrated that ferroptosis is involved in the pathogenesis of SALI.^43,473–479^ Recent studies have also shown the roles of epigenetic regulation of ferroptosis by phosphorylation and acetylation in SALI. NETs induce ferroptosis through METTL3-induced m^6^A modification of GPX4 in the pathogenesis of sepsis-associated ALI.^473^ miR-125b-5p in ADSC-Exos alleviates sepsis-induced ferroptosis in mouse pulmonary microvascular endothelial cells (MPVECs) by upregulating Keap1/Nrf2/GPX4 expression, hence ameliorating sepsis-induced acute lung injury.^480^ Inhibition of MUC1 aggravates lung injury by triggering ferroptosis via a reduction in the phosphorylation level of GSK3β and an increase in Keap1 expression, thereby inhibiting the activation of Nrf2 to decrease GPX4 expression.^474^ FBXW7 mediates the protein degradation of AU-rich element (ARE)-binding factor 1 (AUF1). AUF1 alleviates sepsis-induced acute lung injury through ferroptosis inhibition by upregulating Nrf2 and downregulating ATF3.^475^ ncRNAs are also involved in the modulation of SALI by regulating ferroptosis. Septic conditions were found to upregulate the expression of circEXOC5 in both in vivo and in vitro sepsis models. CircEXOC5 exacerbates SALI through ferroptosis induction by stabilizing ACSL4 mRNA via PTBP1 binding.^479^ Silencing circEXOC5 inhibits lung injury by alleviating ferroptosis, as evidenced by upregulated GPX4 protein expression, decreased ROS levels, and decreased ACSL4 expression.^479^

#### Chronic obstructive pulmonary disease

NF-κB/RelA-mediated PRMT7 upregulation induces monomethylation of histones at enhancers and can regulate Rap1a expression, which is crucial for MAPK signaling downstream of G-protein coupled receptor activation, integrin activation, and the consequent adhesion and migration abilities of monocytes. ALOX5-mediated release of LTB4 in inflammatory macrophages upregulates ACSL4 in AT2 cells, thereby promoting tissue injury through increasing susceptibility to cigarette smoke-induced ferroptosis.^481^ Nrf2 promoter hypermethylation-induced inhibition of Nrf2 expression induces ferroptosis by inhibiting GPX4 in human bronchial epithelial (HBE) cells challenged with cigarette smoke extract.^482^ Cigarette smoke-induced USP14 downregulation aggravates bronchial epithelial cell ferroptosis through proteasomal degradation of MFG-E8.^483^

### Epigenetic and posttranslational modifications regulating ferroptosis in kidney diseases

**Fig. 9:**
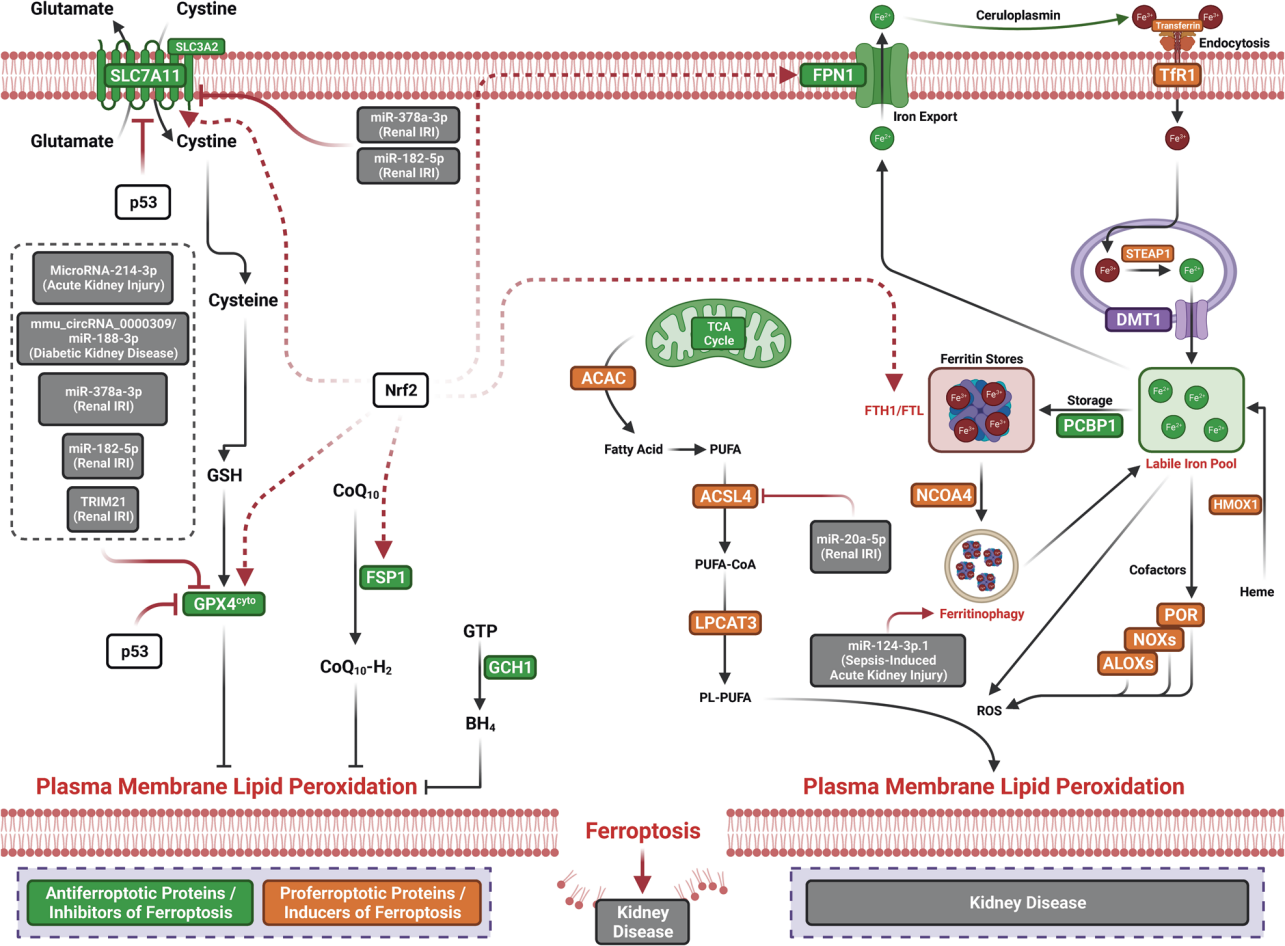
Epigenetic and posttranslational modification of ferroptosis in Kidney disease. AKI Acute kidney injury, ALK activin receptor-like kinase, CHAC1 Cation transport regulator-like protein 1, DNMT1 DNA methyltransferase 1, FMR1 FMRP translational regulator 1, IRI ischemia/reperfusion injury, SAKI sepsis-induced acute kidney injury, TBK1 TANK-binding kinase 1, TIGAR TP53-induced glycolysis and apoptosis regulator

Accumulating studies have shown that ferroptosis plays a role in the genesis of kidney diseases.^35^ Accumulating evidence has shown epigenetic regulation of ferroptosis in kidney diseases, including acute kidney injury (AKI),^484,485^ diabetic nephropathy (DN),^486^ renal fibrosis,^487^ renal IRI (RIRI),^488–492^ sepsis-induced acute kidney injury (SAKI),^493^ toxin-mediated kidney toxicity,^494^ and crystal nephropathies.^495^ Below, we summarize the epigenetic regulation of ferroptosis by phosphorylation, ncRNAs, deacetylation, and ubiquitination in kidney diseases (Table 12 and Fig. 9).

**Table 12:** Epigenetic and posttranslational modification of ferroptosis in kidney diseases

Disease	Modification	Targets	Biological functions	Ref
Acute kidney injury	Phosphorylation	p66Shc	Mitochondrial Translocation of p66Shc Aggravates Cisplatin-induced AKI by Promoting Ferroptosis	^484^
Acute kidney injury	ncRNA	GPX4	MicroRNA-214-3p aggravates cisplatin-induced acute kidney injury through inducing ferroptosis by targeting GPX4.	^485^
Diabetic kidney diseases	ncRNA	GPX4	Downregulated mmu_circRNA_0000309 competitively sponged miR-188-3p, and subsequently promotes GPX4 expression, thereby inactivating ferroptosis-dependent mitochondrial damage and podocyte apoptosis. In addition, GPX4 overexpression neutralized mmu_circRNA_0000309 silence-mediated ferroptosis in germacrone-exposed MPC5 cells.	^486^
Renal fibrosis	Deacetylation	p53	Sirtuin 1-mediated p53 deacetylation alleviates calcium oxalate deposition-induced renal fibrosis through inhibiting ferroptosis in calcium oxalate (CaOx)-induced renal fibrosis.	^487^
Renal IRI	ncRNA	ACSL4	Upregulated miR-20a-5p attenuates IRI and postischemic renal fibrosis through inhibiting dependent ferroptosis via repressing ACSL4.	^488^
Renal IRI	ncRNA	GPX4/SLC7A11	Upregulated miR-182-5p and miR-378a-3p leads to ferroptosis in renal injury through downregulating of GPX4 and SLC7A11, respectively in ischemia/reperfusion-induced rat’s kidney.	^489^
Renal IRI	Ubiquitination		USP14 was upregulated in H/R-induced HK-2 cells and kidney tissues of I/R mice. Inhibition of USP14 suppresses ferroptosis of H/R-induced HK-2 cells.	^490^
Renal IRI	Ubiquitination	GPX4	TRIM21 aggravates ischemia/reperfusion-induced acute kidney injury through promoting ferroptosis via ubiquitylates GPX4.	^491^
Renal IRI	Ubiquitination		USP7 inhibition attenuates I/R-induced renal injury by inhibiting ferroptosis through decreasing ubiquitination of TBK1 and promoting DNMT1-mediated methylation of FMR1.	^492^
Sepsis-induced acute kidney injury	ncRNA	LPCAT3	LPCAT3 by miR-124-3p.1 in acute kidney injury suppresses cell proliferation by disrupting phospholipid metabolism.	^531^
Toxin-mediated kidney toxicity	Phosphorylation	-	Cadmium exposure elevated the level of phosphorylated Smad3 in cadmium-induced HK-2 cell death. Inhibition of ALK4/5 signaling suppresses cadmium-induced cell death in renal proximal tubular epithelial cells via distinct signaling mechanisms via Akt signaling pathways.	^494^
Crystal nephropathies	Phosphorylation	mTOR/S6KP70	TIGAR inhibits adenine-induced ferroptosis in HK-2 cells by activating the mTOR/S6KP70 pathway.	^495^

*ALK* activin receptor-like kinase, CHAC1 Cation transport regulator-like protein 1, *DNMT1* DNA methyltransferase 1, *FMR1* FMRP translational regulator 1, *TBK1* TANK-binding kinase 1, TIGAR TP53-induced glycolysis and apoptosis regulator

#### Acute kidney injury

AKI is caused by sudden loss of excretory kidney function, leading to increased serum creatinine, decreased urine output, or both.^496,497^ The various etiologies of AKI include kidney ischemia, exposure to nephrotoxins, dehydration and sepsis.^498^ AKI develops in ~10–15% of inpatients. Kidney damage or dysfunction can occur over a longer period or follow AKI along a continuum with acute and chronic kidney disease.^496^ Ferroptosis is closely associated with the development of AKI.^44,45,499–501^ Accumulating evidence has revealed epigenetic regulation of ferroptosis by phosphorylation and ncRNAs in AKI. Translocation of P66Shc to mitochondria promotes cisplatin-induced AKI by promoting ferroptosis.^484^ MicroRNA-214-3p aggravates cisplatin-induced AKI through ferroptosis induction by targeting GPX4.^485^

#### Diabetic nephropathy

DN, also called diabetic kidney disease (DKD), results from microvascular damage sustained as a result of diabetes, leading to proximal tubule injury.^502^ DKD is the leading cause of kidney failure worldwide, affecting approximately half of patients with type 2 diabetes and one-third of patients with type 1 diabetes. The characteristics of DKD are the accumulation of ECM, hypertrophy and fibrosis in kidney glomerular and tubular cells.^503^

Increasing evidence has shown that ferroptosis plays a vital role in the genesis of DKD.^504–509^ A recent study also showed the role of epigenetic regulation of ferroptosis by ncRNAs in DKD. Downregulated expression of mmu_circRNA_0000309 results in a decrease in its competitive sponging of miR-188-3p, subsequently promoting GPX4 expression, thereby blocking ferroptosis-dependent mitochondrial damage and podocyte apoptosis. In addition, overexpression of GPX4 counteracts mmu_circRNA_0000309 silencing-mediated ferroptosis in germacrone-exposed MPC5 cells.^486^

#### Renal fibrosis

Renal fibrosis is a common final outcome of a wide variety of chronic kidney disease (CKD) and is characterized by excessive deposition of ECM that leads to tissue scarring.^510^ Evidence has shown that ferroptosis is involved in the pathogenesis of kidney fibrosis.^432,487,511–516^ A recent study also showed the role of epigenetic regulation of ferroptosis by deacetylation in kidney fibrosis. Sirtuin 1-mediated p53 deacetylation was found to attenuate calcium oxalate deposition-induced renal fibrosis by suppressing ferroptosis.^487^

#### Renal ischemia/reperfusion injury

RIRI is the main cause of AKI and contributes to rapid renal dysfunction, morbidity and mortality in patients with a wide range of injuries.^517^ Evidence has demonstrated that ferroptosis is involved in the pathogenesis of RIRI.^518–523^ Increasing evidence shows the roles of epigenetic regulation of ferroptosis by ncRNAs and ubiquitination in RIRI. Upregulated expression of miR-20a-5p attenuates IRI and postischemic renal fibrosis through ferroptosis inhibition by repressing ACSL4.^488^ Upregulated expression of miR-182-5p and miR-378a-3p leads to ferroptosis in the context of renal injury by downregulating GPX4 and SLC7A11, respectively, in the rat kidneys subjected to ischemia/reperfusion.^489^ Increased USP14 expression has been found in H/R-exposed HK-2 cells and in kidney tissues of mice subjected to I/R. Inhibiting USP14 suppresses H/R-induced ferroptosis in HK-2 cells.^490^ TRIM21 aggravates ischemia/reperfusion-induced AKI by promoting ferroptosis via ubiquitination of GPX4.^491^ Inhibiting USP7 attenuates RIRI through ferroptosis inhibition by decreasing the ubiquitination of TBK1 and promoting DNMT1-mediated methylation of FMR1.^492^

#### Sepsis-induced acute kidney injury

SAKI is a common critical illness characterized by rapid sepsis-associated deterioration of renal function; it is one of the leading causes of death worldwide and is a common and life-threatening complication in hospitalized and critically ill patients.^524–527^ SAKI leads to a greatly increased risk of CKD, cardiovascular events and death. Novel findings suggest that ferroptosis plays a pathological role in SAKI.^528–530^ Recent evidence has shown the role of epigenetic regulation of ferroptosis by ncRNAs in SAKI, demonstrating that LPS increases the phosphatidylcholine content and the activity of LPCAT3 and decreases the expression of miR-124-3p.1 in human renal tubular epithelial cells in a SAKI model.^531^ miR-124-3p.1 overexpression in turn suppresses cell injury by inhibiting LPCAT3-related ferroptosis, suggesting that miR-124-3p.1 is a ferroptosis inhibitor.^531^

## Modulation of epigenetic and posttranslational modifications regulating ferroptosis for disease therapy

### Modulation of epigenetic modifications and PTMs regulating ferroptosis for cancer therapy

Epigenetic modifications modulate multiple aspects of cancer biology, and during the last decade, this observation has increased the interest in developing epigenetic strategies to impact tumor growth and drug resistance, thereby combating cancers.^121,122,532^ Unlike genetic mutations, dysregulated epigenetic mechanisms and PTMs can feasibly be targeted by small molecule compounds to treat cancers (Table 13).

**Table 13:** Small molecule compounds target dysregulated epigenetic and posttranslational mechanisms to induce ferroptosis in cancer

Cancer	Compounds	Modification	Targets	Biological functions	Ref
HCC	Tiliroside	Ubiquitination	p62/Keap1/Nrf2	Tiliroside is a potent TBK1 inhibitor and functions as a sensitizer of sorafenib in HCC treatment by targeting TBK1 to induce ferroptosis through decreasing the phosphorylation p62 and the affinity of p62 for Keap1 and promoting Keap1-mediated Nrf2 ubiquitination and degradation	^533^
HCC	Corosolic acid	Ubiquitination	HERPUD1	CA can increase sensitivity to ferroptosis through inhibiting GSH synthesis via HERPUD1, which reduced the ubiquitination of the GSS-associated E3 ubiquitin ligase MDM2, promoting ubiquitination of GSS, thereby inhibiting GSH synthesis to increase ferroptosis susceptibility	^534^
HCC	DHA	Ubiquitination	PEBP1	DHA induced ferroptosis by promoting the formation of PEBP1/15-LO and promoting cell membrane lipid peroxidation. DHA promote PEBP1 protein expression through inhibition of its ubiquitination degradation.	^535^
HCC	Polyphyllin VI	Phosphorylation	STAT3	Polyphyllin VI induces the ferroptosis through inhibiting STAT3 phosphorylation, which inhibits GPX4 expression.	^536^
HCC	Anisomycin	Phosphorylation	p38MAPK	Anisomycin activates p38MAPK to induce ferritinophagy through the phosphorylation of histone H3 on serine 10 (p-H3S10).	^537^
NSCLC	Bufotalin	Ubiquitination	GPX4	Bufotalin induces ferroptosis by facilitating the ubiquitination and degradation of GPX4.	^538^
NSCLC	Sanguinarine	Ubiquitination	GPX4	Sanguinarine triggered ferroptosis through decreasing the protein stability of GPX4 through E3 ligase STUB1-mediated ubiquitination and degradation of endogenous GPX4.	^539^
NSCLC	Selenite	Methylation	p38MAPK	Selenite induces ferroptosis through activating p38-ATF4-DDIT3 axis in the unfolded protein response. Selenite also altered cellular DNA methylation machinery through downregulating DNMT1 and upregulating TET1, though not as a major mechanism of its activity. Low-dose selenite synergized with osimertinib in EGFR-mutant H1975, and with adagrasib in KRAS-mutant H358, with stronger synergism observed in H1975.	^540^
BC	Eupaformosanin	Ubiquitination	p53	Eupaformosanin significantly inhibited the viability of triple-negative breast cancer (TNBC) cells through inducing ferroptosis via ubiquitination of mutant p53.	^541^
BC	DMOCPTL	Ubiquitination	GPX4	DMOCPTL induce ferroptosis through ubiquitination of GPX4.	^542^
BC	NN3	Ubiquitination	p53/SLC7A11	NN3 triggers ubiquitination and proteasome-mediated degradation of PARP1. NN3 exhibited a unique antitumor mechanism in p53-positive breast cancer cells that effectively promoted ferroptosis by downregulating the SLC7A11 pathway.	^543^
BC	Ketamine	Acetylation	KAT5	Ketamine induces ferroptosis through inhibiting the expression of GPX4 by attenuating KAT5 on the promoter region of GPX4, repressing the enrichment of histone H3 lysine 27 acetylation (H3K27ac) and RNA polymerase II (RNA pol II)	^544^
GBM	ALZ003	Ubiquitination	Androgen receptor	ALZ003 induces FBXL2-mediated AR ubiquitination and degradation. ALZ003 significantly inhibited the survival of glioblastomathrough inducing ferroptosis	^545^
Glioma	Paeoniflorin	Ubiquitination	NEDD4L/STAT3	Paeoniflorin might function as an effective drug for glioma by inducing ferroptosis via upregulation of NEDD4L and mediates the ubiquitination of STAT3 repression of Nrf2, GPX4	^546^
GBM	Myrislignan	Phosphorylation	NF-κB	Myrislignan inhibited the activation of NF-κB signaling by blocking the phosphorylation of p65 protein and induced ferroptosis through the Slug-SLC7A11 signaling pathway in GBM cells	^547^
OC	Eriodictyol	Phosphorylation	Nrf2	Eriodictyol induces ferroptosis through downregulating Nrf2 phosphorylation,thereby decreasing protein levels of SLC7A11 and GPX4	^550^
Thyroid cancers	RSL3	Phosphorylation	mTOR	RSL3 activate ferroptosis through suppressing mTOR signaling pathway triggered autophagy. GPX4 genetic knockdown mirrored RSL3 effect on mTOR pathway suppression.	^551^
Pancreatic cancer	QD394	Phosphorylation	STAT3	QD394 induces ferroptosis through inhibiting STAT3 phosphorylation, thereby inhibiting GPX4	^548^
ESCC	Allicin	Phosphorylation	AMPK	Allicin may induce ferritinophagy through increasing AMPK phosphorylation and decreasing mTOR	^549^

*ALZ003* a curcumin analog, *Anisomycin* an agonist of p38 mitogen-activated protein kinase (MAPK), *BC* Breast cancer, *ESCC* esophageal squamous cell carcinoma, *GBM* Glioblastoma, *DHA* Dihydroartemisinin, *DMOCPTL* a derivative of natural product parthenolide, *NN3 PARP1* proteolysis-targeted chimaera (PROTAC), *OC* ovarian cancer, *Polyphyllin* VI STAT3 inhibitor, *QD394* a quinazolinedione reactive oxygen species inducer

#### Hepatocellular carcinoma

Tiliroside, a potent TBK1 inhibitor, increases the sensitivity of HCC to sorafenib by targeting TBK1 to induce ferroptosis by decreasing the phosphorylation of p62 and the affinity of p62 for Keap1 and promoting Keap1-mediated Nrf2 ubiquitination and degradation in HCC.^533^ Corosolic acid can increase the sensitivity of HCC to ferroptosis by inhibiting GSH synthesis via HERPUD1, which decreases MDM2 ubiquitination and promotes the ubiquitination of GSS, thereby inhibiting GSH synthesis to enhance ferroptosis in HCC.^534^ DHA triggers ferroptosis by enhancing the formation of the PEBP1/15-LO complex and promoting cell membrane LPO. DHA promotes PEBP1 protein expression through inhibition of its ubiquitination and degradation.^535^ Polyphyllin VI induces ferroptosis by inhibiting STAT3 phosphorylation, which inhibits GPX4 expression in HCC.^536^ Anisomycin activates p38MAPK to induce ferritinophagy through the phosphorylation of histone H3 on serine 10 (p-H3S10) in HCC.^537^

#### Non-small cell lung carcinoma

Bufotalin induces ferroptosis by facilitating the ubiquitination and degradation of GPX4 in NSCLC.^538^ Sanguinarine triggers ferroptosis by destabilizing GPX4 by promoting its ubiquitination and degradation mediated by its E3 ligase STUB1 in NSCLC.^539^ Selenite induces ferroptosis by activating the p38-ATF4-DDIT3 axis. Selenite also alters the cellular DNA methylation machinery by upregulating TET1 and downregulating DNMT1. Low-dose selenite treatment enhances the antitumor activity of osimertinib in EGFR-mutant H1975 NSCLC cells and of adagrasib in KRAS-mutant H358 NSCLC cells.^540^

#### Breast cancer

Eupaformosanin significantly decreases the viability of TNBC cells by inducing ferroptosis via ubiquitination of mutant p53.^541^ DMOCPTL induces ferroptosis through ubiquitination of GPX4 in breast cancer cells.^542^ NN3 kills p53-positive breast cancer cells by promoting ferroptosis through downregulation of the SLC7A11 pathway by triggering the ubiquitination and proteasome-dependent degradation of PARP1.^543^ Ketamine induces ferroptosis through GPX4 repression by decreasing the occupancy of KAT5 in its promoter region, thus inhibiting the enrichment of H3K27ac and RNA pol II, in breast cancer cells.^544^

#### Glioblastoma/glioma

ALZ003 induces FBXL2-mediated AR ubiquitination and degradation. ALZ003 significantly inhibits glioblastoma cell survival by inducing ferroptosis.^545^ Paeoniflorin inhibits glioma growth by inducing ferroptosis through upregulation of NEDD4L to mediate STAT3 ubiquitination-mediated repression of Nrf2 and GPX4 in glioma.^546^ Myrislignan induces ferroptosis through the Slug-SLC7A11 axis by inactivating NF-κB signaling through blockade of p65 phosphorylation in GBM cells.^547^

#### Pancreatic cancer

QD394 induces ferroptosis by inhibiting STAT3 phosphorylation, thereby inhibiting GPX4 expression in pancreatic cancer.^548^

#### Esophageal squamous cell carcinoma (ESCC)

Allicin may induce ferritinophagy by increasing AMPK phosphorylation and decreasing mTOR in ESCC.^549^

#### Ovarian cancer

Eriodictyol induces ferroptosis by downregulating Nrf2 phosphorylation, thereby decreasing the protein levels of SLC7A11 and GPX4 in ovarian cancer.^550^

#### Thyroid cancer

RSL3 activates ferroptosis by suppressing mTOR-triggered autophagy. Inhibiting GPX4 mirrored the effect of RSL3 on mTOR pathway suppression in thyroid cancer.^551^

### Modulation of epigenetic and posttranslational modifications regulating ferroptosis for CNS disease therapy

A therapeutic regimen for pharmacologically inhibiting ferroptosis to treat disease has been convincingly established in CNS diseases, especially in neurodegenerative diseases and stroke.^19^ Small molecule compounds also feasibly target dysregulated epigenetic mechanisms and PTMs in CNS diseases (Table 14).

**Table 14:** Targeting dysregulated epigenetic and posttranslational modification by small molecule compounds to treat CNS, cardiovascular, liver, lung, and kidney diseases

Diseases	Compounds	Modification	Targets	Biological functions	Ref
ICH	Paeonol	Ubiquitination	ACSL4	Paeonol inhibits ferroptosis of neurons in ICH through downregulating HOTAIR-dependant UPF1-mediated degradation of ACSL4.	^312^
ICH	Isorhynchophylline	ncRNA	p53/SLC7A11	Isorhynchophylline attenuates cell damage through inhibiting ferroptosis via upregulating miR-122-5p and SLC7A11 mRNA, and inhibiting p53 expression in ferric ammonium citrate-treated hippocampal HT-22 cells. The protective effects of IRN against FAC-induced ferroptosis were weakened by miR-122-5p knockdown.	^313^
PD	(-)-Clausenamide	Phosphorylation	ALOX5	Clau directly interactes with the Ser663 of ALOX5, the PKCα-phosphorylation site, and thus prevented the nuclear translocation of ALOX5.	^240^
TBI	Melatonin	ncRNA	5-LOX	Melatonin improves brain function of mice after TBI through inhibiting ferroptosis and endoplasmic reticulum (ER) stress via reducing circPtpn14 (mmu_circ_0000130), which sponge miR-351-5p to downregulate 5-LOX.	^589^
MS	UNC0642	Methylation	GPX4	G9a inhibitor restored anti-ferroptotic gene expression, reduced inflammation-induced neuronal loss, and improved clinical outcome. Similarly, neuronal anti-ferroptotic gene expression was reduced in MS brain tissue and was boosted by G9a inhibition in human neuronal cultures.	^378^
Aortic dissection	BRD4770	Methylation	System Xc^-^-GPX4; FSP1-CoQ10; GCH1-BH4	BRD4770 attenuated aortic dilation through inhibiting the inflammatory response and ferroptosis.	^590^
Aortic dissection	Liproxstatin-1	Phosphorylation	ALOX5	Liproxstatin-1 largely abrogates BAPN-induced AAD in mice. These results suggest that inhibition of METTL3 or ferroptosis is an effective intervention strategy for AD.	^422^
	Fer-1	Methylation		Fer-1 abolishes detrimental role of PRMT4-mediated ferroptosis in DIC.	^411^
DIC	Metformin	phosphorylation	AMPKα2	metformin (MET) treatment could inhibit ferroptosis and improve cardiac function via activating AMPKα2 phosphorylation.	^412^
DIC	Ferrostatin-1	Ubiquitination		Ferrostatin-1 suppresses exacerbation of DOX-induced myocardial damage in MITOL-knockout hearts.	^414^
Cardiac Fibrosis	C646	Acetylation	p53	Inhibition of p53 acetylation by C646 significantly alleviated ferroptosis in H9c2 myofibroblasts.	^416^
Cardiac Fibrosis	Ferrostatin-1	Acetylation	p53	Treatment of SIRT3-cKO mice with ferrostatin-1 led to a significant reduction in ferroptosis and cardiac fibrosis.	^416^
Cardiac Fibrosis	Ferrostatin-1	ncRNA	GPX4	Fer-1 promotes the antioxidant capacity of cardiac fibroblasts, reduced GPX4-mediated ferroptosis and alleviated I/R-induced CF.	^417^
HFD-induced cardiac injury	Celastrol	Phosphorylation	AKT/GSK3β	Celastrol confers ferroptosis resistance via AKT/GSK3β signaling in high-fat diet-induced cardiac injury.	^591^
HFD-induced cardiac injury	Piperlongumine	Phosphorylation	STAT3	Piperlongumine protects cardiomyocytes from ferritinophagy-mediated ferroptosis both in vitro and in vivo through reducing phosphorylated STAT3 levels.	^420^
MIRI	Compound 968	ncRNA	GLS2	Inhibition of miR-190a-5p caused upregulation of GLS2, resulting in decreased ferroptosis, which could be blocked by GLS2 inhibitor compound 968.	^399^
Acute liver injury	Sulforaphane	Phosphorylation	BECN1	Nrf2-dependent autophagy activation by sulforaphane disrupted SLC7A11 binding to S93-phosphorylated BECN1 and increased SLC7A11 membrane transfer to inhibit ferroptosis. Activation of Nrf2 not only upregulates the expression of SLC7A11, GPX-4 and autophagy-related proteins, but also destroys the binding of SLC7A11 and BECN1 by inducing autophagy, thereby promoting SLC7A11 membrane transfer and GSH synthesis, and finally suppressing ferroptosis.	^441^
Liver fibrosis	Berberine	Ubiquitination	ferritin	Berberine alleviates mouse liver fibrosis by inducing ferrous redox to activate ROS-mediated HSC ferroptosis.	^592^
Liver fibrosis	Recombinant FGF21	Ubiquitination		Recombinant FGF21 inhibits liver fibrosis by inhibiting hepatocytes ferroptosis through promotiing HO-1 ubiquitination and degradation and Nrf2 activation.	^593^
Liver fibrosis	Artemether	Phosphorylation	AMPKα2	Artemether alleviates liver fibrosis in vivo and in vitro through inducing HSC ferroptosis via inhibiting the ubiquitination of IRP2, thereby inducing the increase of iron in HSC.	^594^
NAFLD	Urolithin C	Phosphorylation	AMPK	Urolithin C inhibits NAFLD via regulating AMPK-ferroptosis axis, maintaining intestinal mucosal barrier and counteracting gut dysbiosis.	^595^
Toxin -mediated hepatic toxicity	Fer-1 and tBHQ	Phosphorylation	Nrf2	Ethyl carbamate-induced liver dysfunction and inflammation, accompanied with oxidative stress, ferroptosis and downregulated Nrf2 signaling in Balb/c mice, which could be effectively reversed by Fer-1 and tBHQ pretreatment.	^464^
Toxin -mediated hepatic toxicity	Fer-1 or tBHQ	Phosphorylation	Nrf2	Fer-1 or tBHQ inhibit Glyphosate triggered ferroptosis-induced liver damage in a mouse mode	^465^
Acute liver failure	AGK2	Phosphorylation	MFN2/GPX4	AGK2 inhibits thioacetamide-induced acute liver failure via regulating the MFN2-PERK axis and ferroptosis signaling pathway.	^596^
Acute lung injury	MitoQ	Phosphorylation	-	MitoQ pretreatment countered As-induced pulmonary ferroptosis and ALI.	^469^
Acute lung injury	Fer-1	Ubiquitination	-	Arsenic-triggered mitochondria damage and ferroptosis were mitigated in Fer-1 pretreated-MLE-12 cells.	^469^
Acute lung injury	PERK inhibitor	Phosphorylation	PERK	PERK inhibitor and Mfn-2-overexpression all mitigated As-induced ferroptosis in MLE-12 cells.	^469^
Acute lung injury	Ferrostatin-1	-	-	Ferrostatin-1 alleviates lung injury and pulmonary edema.	^470^
Sepsis-Induced Acute Lung Injury	Obacunone	Ubiquitination	Nrf2	Obacunone alleviates lipopolysaccharide-induced acute lung injury through inhibiting ferroptosis via inhibiting Nrf2 ubiquitinated proteasome degradation, thereby upregulating GPX4 and SLC7A11.	^597^
Pulmonary epithelial senescence	Melatonin	Acetylation	SIRT3/p53	SIRT3 activation by melatonin deacetylated P53 at lysines 320 (K320), thus blocking senescence and ferroptosis.	^598^
COPD	rhMFG-E8	Ubiquitination	MFG-E8	rhMFG-E8 ameliorates ferroptosis induced by cigarette smoke extract in BEAS-2B cells and HBE cells.	^483^
Diabetic nephropathy	Germacrone	-	-	Germacrone inhibits DN through inhibiting ferroptosis via mmu_circRNA_0000309 silence or miR-188-3p mimics abrogated the antiapoptosis and anti-injury effects of germacrone through aggravating mitochondria damage, and elevating reactive oxygen species and ferroptosis-related protein levels.	^486^
Renal IRI	IU1	Ubiquitination	USP14	IU1, a small molecule inhibitor of USP14 and NAC effectively alleviated renal injury of I/R mice.	^490^
Renal IRI	Fedratinib	Ubiquitination	GPX4	A JAK2 inhibitor Fedratinib downregulates TRIM21 expression and reduce damage both in vivo and in vitro, which is correlated with the upregulation of GPX4.	^491^
Toxin-mediated kidney toxicity	SB431542 or SB505124	-	ALK4/5	ALK4/5 kinase inhibitors, SB431542 or SB505124, suppressed cadmium-induced HK-2 cell death	^494^
Toxin-mediated kidney toxicity	SIS3	Phosphorylation	TGFβ1	Cadmium-induced cell death was attenuated by treatment with SIS3, a selective inhibitor of TGFβ1-dependent Smad3 phosphorylation.	^494^
Folic Acid-Induced Kidney Injury	Roxadustat (FG-4592)	Phosphorylation	Akt/GSK-3β/Nrf2	Roxadustat (FG-4592) attenuates Folic Acid-Induced Kidney Injury through inhibitng ferroptosis via Akt/GSK-3β-mediated Nrf2 activation.	^599^
Acute kidney injury	Baicalein	Acetylation	p53	Baicalein ameliorates polymyxin B-induced acute renal injury through inhibiting ferroptosis via reducing p53 K382 acetylation via upregulation of SIRT1 expression.	^600^
Acute kidney injury	Dihydromyricetin	-	-	Dihydromyricetin attenuates cisplatin-induced acute kidney injury by inhibiting ferroptosis via	^601^
Acute kidney injury	myo-inositol	Ubiquitination	NOX4	myo-inositol ameliorates cisplatin-induced acute kidney injury through inhibitng ferroptosis via promotes CHIP-mediated ubiquitination of NOX4.	^602^
Diabetic kidney disease	Dapagliflozin	Ubiquitination	SLC40A1	Dapagliflozin ameliorated tubular injury by inhibiting ferroptosis through stabilize SLC40A1 via reduce ubiquitination degradation.	^603^
Diabetic kidney disease	Ginkgolide B	Ubiquitination	GPX4	Ginkgolide B alleviates diabetic kidney disease through inhibiting ferroptosis by inhibiting GPX4 ubiquitination.	^604^
Diabetic kidney disease	Schisandrin A	-	-	Schisandrin A attenuates diabetic kidney disease Ferroptosis and NLRP3 Inflammasome-Mediated Pyroptosis in Diabetic Nephropathy through Mitochondrial Damage by AdipoR1 Ubiquitination	^605^
Adriamycin-Induced Renal Damage	Astragaloside IV	Phosphorylation	PI3K/Akt /Nrf2	Astragaloside IV attenuates Adriamycin-Induced Renal Damage through inhibiting ferroptosis via activations of the PI3K/Akt and Nrf2	^606^

*AGK2* an inhibitor for SIRT2, *BECN1* coiled-coil myosin-like BCL2-interacting protein

#### Intracerebral hemorrhage

Paeonol, a naturally occurring phenolic agent extracted from Cortex Moutan, exerts neuroprotective effects in patients with many CNS diseases.^552–573^ Paeonol inhibits ferroptosis in neurons in ICH by downregulating HOTAIR-dependent UPF1-mediated degradation of ACSL4.^312^ Isorhynchophylline (IRN), a tetracyclic oxindole alkaloid extracted from *Uncaria rhynchophylla*, has diverse biological activities, such as antioxidant, neuroprotective and anti-inflammatory activities.^574–583^ Isorhynchophylline attenuates cell damage by inhibiting ferroptosis via upregulation of miR-122-5p and SLC7A11 mRNA expression and inhibiting p53 expression in ferric ammonium citrate (FAC)-treated hippocampal HT-22 cells. The protective effects of IRN against FAC-induced ferroptosis were weakened by miR-122-5p knockdown.^313^

#### Parkinson’s disease

(-)Clausenamide (Clau), a scavenger of lipid peroxidation products, is an alkaloid that is isolated from the plant *Clausena lansium* (Lour.) and exhibits neuroprotective activities both in vivo and in vitro.^574–583^ Clau prevents the nuclear translocation of ALOX5 by directly interacting with Ser663 of ALOX5, the PKCα phosphorylation site.^240^

#### Traumatic brain injury

Melatonin, an indole hormone that contributes to neuroprotection, exerts a neuroprotective effect by inhibiting ferroptosis.^584–587^ A recent study showed that melatonin ameliorates neurological deficits by inhibiting neuroinflammation and ferroptosis via MT2/IL-33/ferritin H signaling in TBI.^588^ Melatonin improves brain function in mice after TBI by inhibiting ferroptosis and ER stress by reducing the expression of circPtpn14 (mmu_circ_0000130), which sponges miR-351-5p to downregulate 5-LOX.^589^

#### Multiple sclerosis

The G9a inhibitor UNC0642 reduces inflammation-induced neuronal loss and improves clinical outcomes through restoring the expression of antiferroptotic genes. The neuronal expression of antiferroptotic genes was found to be decreased in MS brain tissue but was increased by G9a inhibition in human neuronal cultures.^378^

### Modulation of epigenetic modifications regulating ferroptosis for CVD therapy

Increasing evidence has suggested that ferroptosis may represent a therapeutic approach for CVD. Some small molecule compounds can target dysregulated epigenetic mechanisms in liver diseases (Table 14).

#### Aortic dissection

BRD4770 attenuates aortic dilation by inhibiting the inflammatory response and ferroptosis.^590^ Liproxstatin-1 inhibits BAPN-induced aortic aneurysm and dissection (AAD) in mice. Inhibition of METTL3 expression or ferroptosis is an effective intervention strategy for AD.^422^ Ferrostatin-1 (Fer-1) abolishes the detrimental role of PRMT4-mediated ferroptosis in DIC.^411^

#### Disseminated intravascular coagulation (DIC)

Metformin (MET) improves cardiac function by inhibiting ferroptosis through activation of AMPKα2 phosphorylation.^412^ Ferrostatin-1 suppresses the exacerbation of DOX-induced myocardial damage in MITOL-knockout hearts.^414^

#### Cardiac fibrosis

C646 attenuates ferroptosis by inhibiting p53 acetylation in H9c2 myofibroblasts.^416^ Treatment of SIRT3-cKO mice with ferrostatin-1 was found to lead to significant reductions in ferroptosis and CF.^416^ Ferrostatin-1 increases the antioxidant capacity of cardiac fibroblasts, reduces GPX4-mediated ferroptosis and alleviates I/R-induced CF.^417^

#### HFD-induced cardiac injury

Celastrol induces ferroptosis resistance via AKT/GSK3β signaling in HFD-induced cardiac injury.^591^ Piperlongumine inhibits ferritinophagy-mediated ferroptosis in cardiomyocytes by reducing the phosphorylated STAT3 level.^420^

#### Myocardial ischemia/reperfusion injury

Inhibition of miR-190a-5p upregulates GLS2, resulting in decreased ferroptosis, and this effect could be blocked by the GLS2 inhibitor compound 968.^399^

### Modulation of epigenetic modification and posttranslational modifications regulating ferroptosis for liver disease therapy

Emerging evidence has suggested that ferroptosis may represent a novel therapeutic approach for liver diseases. Dysregulated epigenetic mechanisms and PTMs can also be feasibly targeted by small molecule compounds in liver diseases (Table 14).

#### Liver fibrosis

Berberine attenuates liver fibrosis in mice by inducing ROS-mediated HSC ferroptosis.^592^ Recombinant FGF21 inhibits liver fibrosis by inhibiting hepatocyte ferroptosis through promotion of HO-1 ubiquitination and degradation and Nrf2 activation.^593^ Artemether alleviates liver fibrosis in vivo and in vitro by inducing HSC ferroptosis through inhibition of IRP2 ubiquitination, thereby inducing an increase in the iron content in HSCs.^594^

#### Nonalcoholic fatty liver disease

Urolithin C ameliorates NAFLD by regulating the AMPK-ferroptosis axis, maintaining the intestinal mucosal barrier and counteracting gut dysbiosis.^595^

#### Toxin-mediated hepatic toxicity

Ferrostatin-1 and tert-butylhydroquinone (tBHQ) reverse ethyl carbamate-induced liver dysfunction and inflammation by inhibiting ferroptosis in BALB/c mice.^464^ Ferrostatin-1 and tBHQ were also found to attenuate liver damage resulting from glyphosate-triggered ferroptosis in a mouse model.^465^

#### Acute liver failure

AGK2 alleviates thioacetamide-induced acute liver failure by inhibiting ferroptosis by regulating the MFN2-PERK pathway.^596^

### Modulation of epigenetic and posttranslational modifications regulating ferroptosis for lung disease therapy

Emerging evidence has suggested that targeting ferroptosis may represent a novel therapeutic approach for lung diseases. Dysregulated epigenetic mechanisms and PTMs can also be feasibly targeted by small molecule compounds in lung diseases (Table 14).

#### Acute lung injury

MitoQ pretreatment counteracts arsenic-induced pulmonary ferroptosis and ALI.^469^ Ferrostatin-1 mitigates arsenic-triggered mitochondrial damage and ferroptosis in MLE-12 cells.^469^ PERK inhibitors mitigate arsenic-induced ferroptosis in MLE-12 cells.^469^ Ferrostatin-1 alleviates lung injury and pulmonary edema.^470^

#### Sepsis-induced acute lung injury

Obacunone alleviates LPS-induced acute lung injury by inhibiting ferroptosis through inhibition of the ubiquitin-mediated proteasomal degradation of Nrf2, thereby upregulating GPX4 and SLC7A11.^597^

#### Pulmonary epithelial senescence

SIRT3 activation by melatonin alleviates PM2.5-induced senescence and ferroptosis in mice by deacetylating p53 at lysine 320 (K320).^598^

#### Chronic obstructive pulmonary disease

hMFG-E8 ameliorates ferroptosis induced by cigarette smoke extract in BEAS-2B cells and HBE cells.^483^

### Modulation of epigenetic and posttranslational modifications regulating ferroptosis for kidney disease therapy

An increasing body of evidence indicates that targeting ferroptosis may represent a therapeutic approach for kidney disease. Dysregulated epigenetic mechanisms and PTMs can also be feasibly targeted by small molecule compounds in kidney diseases (Table 14).

#### Diabetic nephropathy

Germacrone ameliorates DN by inhibiting ferroptosis through upregulation of mmu_circRNA_0000309, which sponges miR-188-3p, subsequently upregulating GPX4 expression.^486^

#### Renal IRI

The small molecule USP14 inhibitors IU1 and NAC ameliorate renal injury in I/R mice.^490^ The JAK2 inhibitor fedratinib downregulates TRIM21 expression and reduces damage both in vivo and in vitro, and these effects are correlated with upregulation of GPX4.^491^

#### Toxin-mediated kidney toxicity

SB431542 and SB505124, ALK4/5 kinase inhibitors, suppress cadmium-induced HK-2 cell death.^494^ SIS3, a selective inhibitor of TGFβ1-dependent Smad3 phosphorylation, attenuates cadmium-induced cell death.^494^

#### Folic acid-induced kidney injury

Roxadustat (FG-4592) ameliorates folic acid-induced kidney injury by repressing ferroptosis via Akt/GSK-3β-mediated Nrf2 activation.^599^

#### Acute kidney injury

Baicalein ameliorates polymyxin B-induced acute renal injury by inhibiting ferroptosis through a reduction in p53 K382 acetylation via upregulation of SIRT1 expression.^600^ Dihydromyricetin attenuates cisplatin-induced AKI by inhibiting ferroptosis.^601^ Myo-inositol ameliorates cisplatin-induced AKI by inhibiting ferroptosis through the promotion of CHIP-mediated ubiquitination of NOX4.^602^

#### Diabetic kidney disease

Dapagliflozin ameliorates tubular injury by inhibiting ferroptosis through stabilization of SLC40A1 by reducing its ubiquitin-mediated degradation.^603^ Ginkgolide B alleviates DKD by inhibiting ferroptosis through inhibition of GPX4 ubiquitination.^604^ Schisandrin A attenuates ferroptosis and pyroptosis in DKD through attenuation of mitochondrial damage via AdipoR1 ubiquitination.^605^

#### Adriamycin-induced renal damage

Astragaloside IV attenuates adriamycin-induced renal damage by inhibiting ferroptosis through activation of PI3K/Akt and Nrf2.^606^

## Concluding remarks and future perspectives

Epigenetic modifications and PTMs are essential for the maintenance of physical homeostasis and are implicated in the pathology of a variety of diseases. Accumulating evidence indicates that epigenetic modifications and PTMs play vital roles in regulating ferroptosis by controlling the expression of ferroptosis-associated genes in cancer, NSDs, CVDs, liver diseases, lung diseases, and kidney diseases. The present review highlights the crucial roles of epigenetic modifications and PTMs regulating ferroptosis in tumorigenesis and the genesis of NSDs, CVDs, liver diseases, lung diseases, and kidney diseases and clarified the regulatory interactions between epigenetic modifications and PTMs regulating ferroptosis in the genesis of these diseases. However, epigenetic modification-mediated regulation of ferroptosis in cancers is an emerging field and is still in its infancy. It is crucial to determine whether other epigenetic regulators play a role in regulating ferroptosis in cancers. First, in addition to ubiquitination, phosphorylation, acetylation, SUMOylation, O-GlcNAcylation, methylation, and ncRNA regulation, whether other epigenetic modifications, including ISGylation and lactylation, are involved in regulating ferroptosis remains unclear. Second, specific epigenetic modifications and PTMs regulate ferroptosis in specific cancers, NSDs, CVDs, liver diseases, lung diseases, and kidney diseases, and whether these epigenetic regulatory mechanisms are actually specific in other types of cancers remains an open question for future investigation. Third, epigenetic modifications and PTMs of targets that induce or inhibit ferroptosis have been identified, and dysregulated epigenetic modifications and PTMs can be feasibly targeted by small molecule compounds. Epigenetic drugs have exhibited viable therapeutic potential for NSDs,^607–610^ CVDs,^611–615^ liver diseases,^616,617^ lung diseases,^618–620^ and kidney diseases^621–623^ in preclinical and clinical trials. Epigenetic drugs targeting epigenetic regulators through modulation of epigenetic mechanisms have been applied and clinically translated for the treatment of hematological malignancies, greatly contributing to the development of antitumor drugs.^118,124,624^ Epigenetic drugs were found to exhibit viable therapeutic potential for solid tumors in preclinical and clinical trials.^118,625^ Combinations of epigenetic drugs with other therapies are being tested in preclinical research and clinical trials for the treatment of solid tumors.^626,627^ According to previous studies on clinical epigenetic drugs for hematological malignancies and studies that have revealed key roles of ferroptosis in mediating antitumor activity, we believe that epigenetic drugs targeting epigenetic regulators and ferroptosis could erase the roadmap to cancer. However, much remains to be done before the practical application of these treatment modalities, and inevitable challenges remain and need further clinical investigation. Herein, we highlight in detail the current efforts to translate this knowledge into clinical benefit for patients. Fourth, whether epigenetic modifications affect multiple ferroptosis regulators and how these different epigenetic modifications and PTMs cooperate with diverse signaling pathways to control the susceptibility of cancer cells to ferroptosis remain unclear. The epigenetic modification network of ferroptosis needs extensive investigation. Fifth, although ferroptosis has recently been confirmed to play a vital role in tumorigenesis and in the genesis of NSDs, CVDs, liver diseases, lung diseases, and kidney diseases, the detailed mechanisms related to ferroptosis and the molecular pathways involved remain open topics for future investigation. Moreover, the mechanisms by which epigenetic modifications and PTM events control the expression of ferroptosis-related genes in cancers, NSDs, CVDs, liver diseases, lung diseases, and kidney diseases remain incompletely elucidated. Determining whether mechanisms of novel epigenetic modifications and PTMs are actually specific in different diseases and cell types needs more systematic and comprehensive investigation.

In conclusion, epigenetic modifications and PTMs play fundamental roles in diseases, such as cancers, NSDs, CVDs, liver diseases, lung diseases, and kidney diseases. More studies on the effects of epigenetic modifications and PTMs on ferroptosis will ensure a better understanding of the pathogenesis of these diseases and identify novel paradigms for their treatment.
